# Evaluation on reprogramed biological processes in transgenic maize varieties using transcriptomics and metabolomics

**DOI:** 10.1038/s41598-021-81637-2

**Published:** 2021-01-21

**Authors:** Wei Fu, Pengyu Zhu, Mingnan Qu, Wang Zhi, Yongjiang Zhang, Feiwu Li, Shuifang Zhu

**Affiliations:** 1 Institute of Plant Quarantine, Chinese Academy of Inspection and Quarantine, Beijing, 100176 China; 2 National Key Laboratory of Plant Molecular Genetics, CAS Center for Excellence in Molecular Plant Sciences, Shanghai, 200032 China; 3 Institute of Agricultural Quality Standard and Testing Technology, Jilin Academy of Agricultural Sciences, Changchun, 130033 Jilin China

**Keywords:** Environmental biotechnology, Metabolomics, Plant biotechnology

## Abstract

Genetic engineering (GM) has great potential to improve maize productivity, but rises some concerns on unintended effects, and equivalent as their comparators. There are some limitations through targeted analysis to detect the UE in genetically modified organisms in many previous studies. We here reported a case-study on the effects of introducing herbicides and insect resistance (*HIR*) gene cassette on molecular profiling (transcripts and metabolites) in a popular maize variety Zhengdan958 (ZD958) in China. We found that introducing *HIR* gene cassette bring a limited numbers of differential abundant genes (DAGs) or differential abundant metabolites (DAMs) between transgenic events and non-transgenic control. In contrast, averaged 10 times more DAGs and DAMs were observed when performed comparison under different growing environments in three different ecological regions of China than the numbers induced by gene effects. Major biological pathways relating to stress response or signaling transduction could explain somehow the effects of growing environments. We further compared two transgenic events mediated ZD958 (GM-ZD958) with either transgenic parent GM-Z58, and other genetic background nonGM-Z58, nonGM-ZD958, and Chang7-2. We found that the numbers of DAGs and DAMs between GM-ZD958 and its one parent maize variety, Z58 or GM-Z58 is equivalent, but not Chang7-2. These findings suggest that greater effects due to different genetic background on altered molecular profiling than gene modification itself. This study provides a case evidence indicating marginal effects of gene pleiotropic effects, and environmental effects should be emphasized.

## Introduction

Genetic engineering plays crucial roles in crop productivity improvement; however, it may possess sometimes unintended effects on the crop quality and the public health. It has been extensively applied in the improved resistance to disease, herbicides as well as crops quality, especially in maize, as the most widely grown crop worldwide, and contributes to around 800 million tons production from 2007 to 2008^1^. Budget allocations for major biotechnology funding agencies has been annually increased in several countries. The Indian government allocates an estimated US$15 million annually on plant biotechnology sector, while the private sector contributes about US$10 million^2^. China currently accelerates its investments in agricultural biotechnology research and is focuses further on the commodities that have been mostly ignored in the laboratories of industrialized countries^2^.

However, the derived products from genetically modified crops (GM) always rises controversy, especially in Europe, despite the absence of evidences proving risks of GM crops. The major concern from public community is about whether GM crops are subjected to critical inspection regarding food safety^3^. It has been agreed internationally to ensure a promising equivalence between GM crops and traditional varieties^4^. The biological effects of GM crops on human health, environment, agronomy, and economy were extensively reported, and some researchers found that no obviously adverse-effects attributed to genetic engineering on the human population^5^. Another concern about the unintended effects on the alteration of DNA sequences, proteins or new metabolites or reprogramed metabolites due to random insertion on genomic regions^6,7^, created by *Agrobacterium*-mediated gene transfer and particle bombardment^8–10^. Therefore, it needs to be deeply investigated via a specific powerful and comprehensive analyzing method to compare the GM crops or products to their counterparts from parental or near isogenic lines^11,12^.

Recently, the roles of emerging “omics” technologies in the assessment of unintended gene effects was extensively proposed, the omic analysis has been successfully applied for the reconstruction of genome-scale networks in some commercialized GM crops^13–15^. However, most studies performed the targeted analysis, which may, have its limitations in detecting unintended effects in genetically modified organisms^12^. Consequently, this prompted us to further assess the non-targeted profiling or fingerprinting technologies that could be used as unbiased analytical approaches to characterize the potential occurrence of the unintended effects caused by introducing exogenous gene or overexpressing endogenous gene^13^.

The objective of this investigation is to gain more insights and perform a cutting edge research using high technologies and targeted omics analysis to a deeply understand of the biology of the Zhengdan958 GM maize by molecular profiling (transcriptomes and metabolomics). Herein, we investigated molecular profiling to provide evidences into the extent of variation in the maize transcriptome and metabolome by analyzing a multiple gene cassette underlying both herbicide and insect resistance (named *HIR* cassette) engineered into different maize backgrounds. These included two transgenic modified lines and the respective control line. All are commercial lines and were grown in different locations in china. We report on the application of transcriptome profiling, and capillary liquid chromatographic/mass spectrometric (LC/MS)-based metabolite profiling for the analysis of metabolome. We performed GO and KEGG analyses to identify the potentially altered biological processes and/or metabolic pathways that were mainly responsible for the differences between GM lines and their respective non-GM maize lines. The data presented serve as an exploratory study into the use of 'omics' approaches for a safety and successfully evaluation of GM crops.

## Results

### Generation of HIR cassette transgenic maize lines

**Figure 1:**
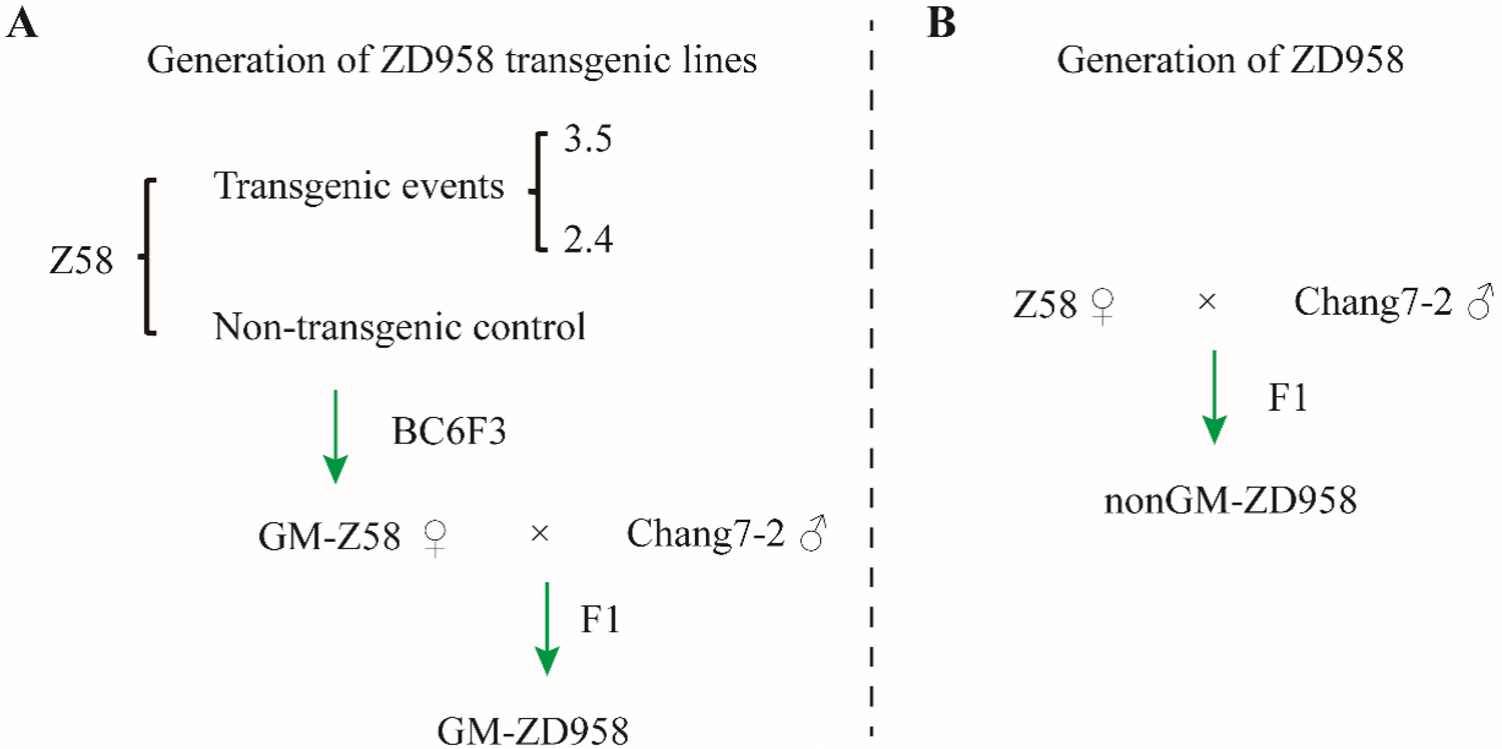
Pipeline of transgenic maize lines in this study. (**A**) The transgenic Zhengdan958 (GM-ZD958) was produced by agrobacteria-mediated transformation into Zheng58 (Z58) and then was used to hybrid with Chang7-2. (**B**) Non-transgenic ZD958 (non-GM ZD958) was used as control produced from Z58 and Chang7-2.

Herbicide and insect resistant are two major concerns in maize breeding. In this study, we transformed a *HIR* gene cassette consisting of *EPSPS* and *Cry1Ab* gene underlying herbicide and insect, respectively, into maize cultivar Zheng58 (Z58), forming a gene modified (GM) line (GM-Z58). Then we created a maize line in BC6F3 generation, GM-ZD958, through crossing GM-Z58 and hybrid non-GM maize variety Chang7-2. In this regard, we performed two independent transgenic events, i.e., event-2.4 and event-3.5 using same vectors harboring same gene, to elucidate the random insertion effects of targeted genomic regions (Fig. 1, Table 1).

**Table 1:** Transgenic and non-transgenic maize lines used in this study.

Type	Material	Transgenic event 3.5	Transgenic event 2.4	Non-GM control	Growth chamber	Field experiments
Inbred line	Zheng 58	√	√	√	√	–
Inbred line	Chang 7-2	–	–	√	√	–
Hybrid variety	Zhengdan 958	√	√	√	√	Beijing, Zhengzhou, Harbin

**Figure 2:**
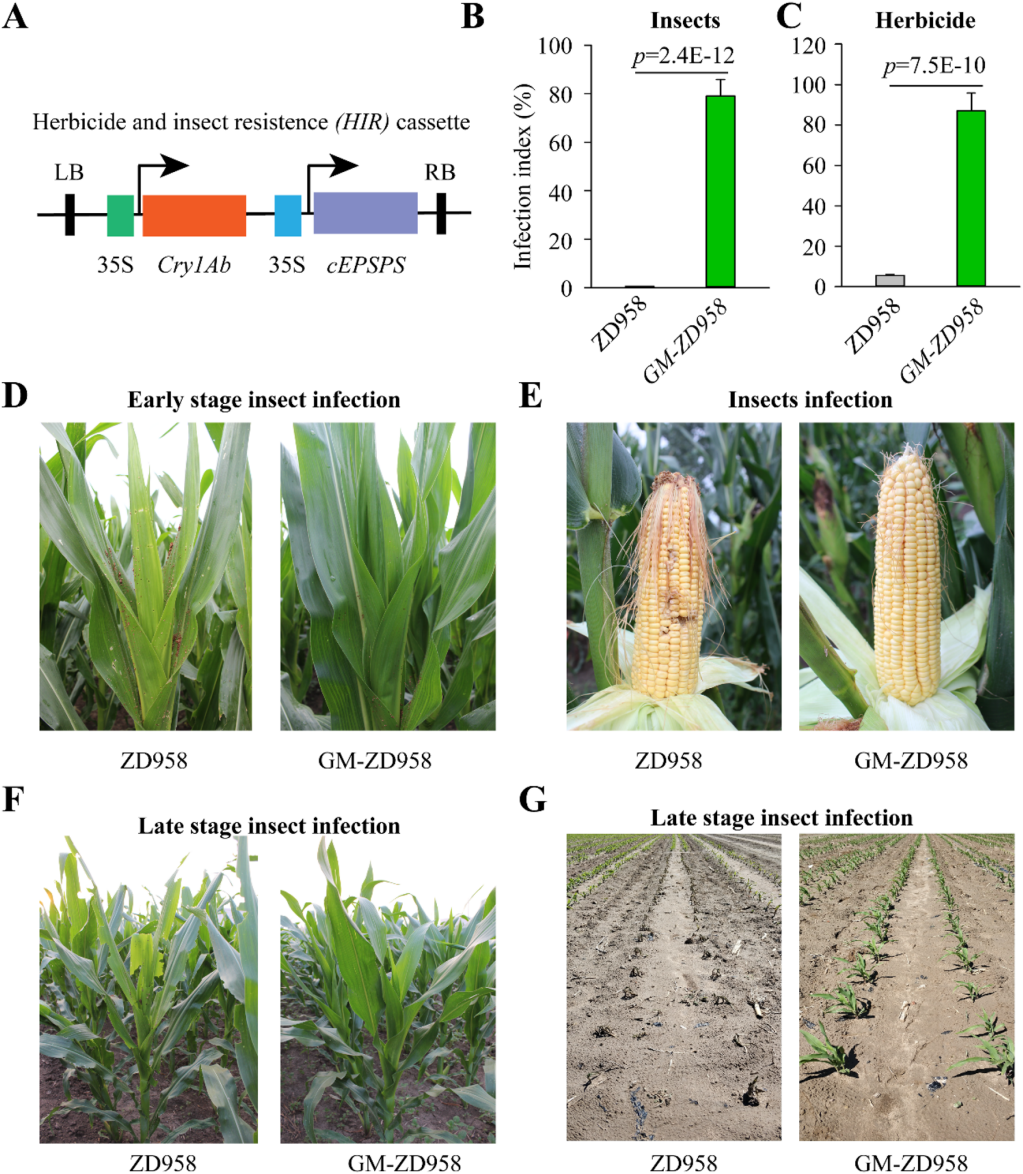
Field performance of herbicides and insect resistance of ZD958 expressing *HIR* gene cassette in gene-modification (GM)-ZD958 maize lines grown in Beijing field (E116.3°, N39.7°), China. (**A**), Schematic map of constructed vector for *HIR* gene cassette. (**B**,**C**), Comparison of infection index to insect (*Bacillus thuringiensis*) and herbicide (glyphosate) resistance in GM-ZD958 from two transgenic events, compared to non-GM ZD958. (**D**,**E**), Field imaging of GM-ZD958 and non-GM ZD958 exposed naturally to insect in canopy in early stage (**D**) and maize seeds during silking stage (**E**). (**F**,**G**), Field imaging of GM-ZD958 and non-GM ZD958 exposed naturally to insect in canopy in late stage (**F**) and performance of non-GM ZD958 and GM-ZD958 subjected to glyphosate treatments.

Transgenic vector harboring *HIR* gene cassette fusion with 35S promoter were constructed (Fig. 2A). The derived maize variety, GM-ZD958 for two transgenic events (event-2.4 and event 3.5) exerted stronger resistance to either herbicide or insect than GM-Z58 and Chang7-2 (Fig. 2B–G). Non-GM ZD958 maize lines were used as control (CK). We first performed a global-mRNA expression analysis on totally 36 datasets of experimental sites, maize genetic background, and transgenic vectors/CK. Our results suggest that there are averaged 47.3 million and 46.2 million for raw and clean reads across 36 datasets, respectively. The account of clean reads over total reads is 97.6% (Supplementary Table S2). Mapped reads and ratio of mapped against raw reads are 42 million and 91.6%, respectively. Among mapped reads, uniquely mapped reads account for 95.5%. In addition, 96% mapped reads were identified on exon of gene body, while only 1.8% and 1.9% were identified in intron and intergenic region of gene body, respectively (Supplementary Table S2).

### Different transgenic events possess similar DAGs and DAMs

**Figure 3:**
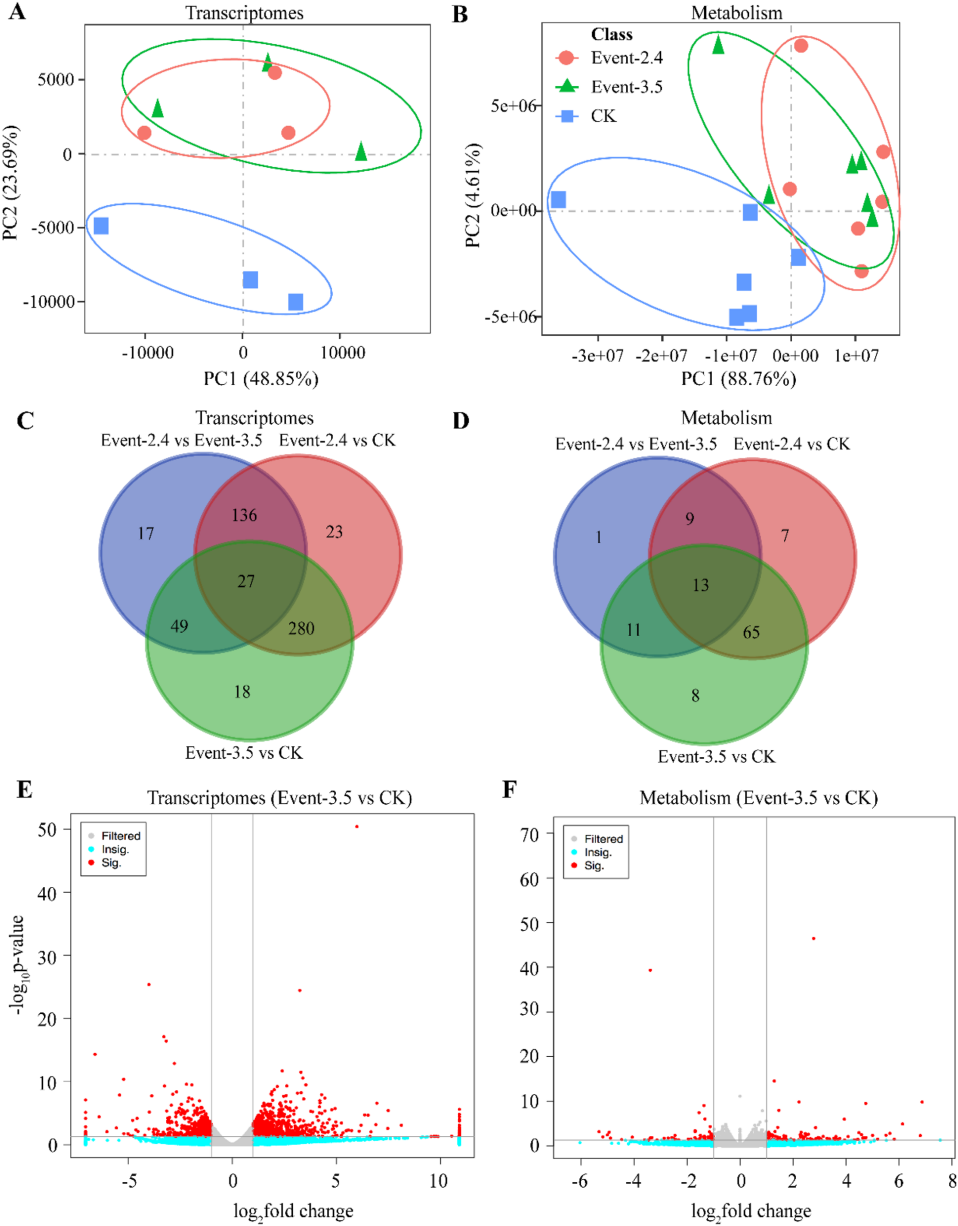
PCA analysis on differently abundant genes and metabolites in ZD958 maize transgenic lines grown in Beijing. (**A**), transcriptomes analysis on different experimental sites across different transgenic lines of transgenic event-2.4 (Event-2.4), transgenic event-3.5 (Event-3^.^5) and CK; n = 3. (**B**), metabolomic analysis on different experimental sites across different transgenic lines of 2.4, 3.5 and ck. *n* = 6. (**C**), overlapping genes between different ZD958 transgenic events and non-GM ZD958 (CK) grown in Beijing. The detailed overlapping genes are referred to Supplementary Table S2. (**D**), overlapping metabolites. The detailed overlapping metabolites are referred to Supplementary Table S3. (**E**,**F**), differential abundant genes and metabolites between event-3.5 and CK. The differential abundant genes (DAGs) and metabolites (DAMs) were indicated in red scatters.

We have grown the transgenic lines against ZD958 in Beijing using two transgenic events and non-GMZD958 (CK) (Fig. 3). Our results based on principal components analysis (PCA) suggest that two transgenic events show similar clusters of differently abundant genes (DAGs) and differently abundant metabolites (DAMs), while non-GM ZD958 were separated apart from the two transgenic events (Fig. 3A,B). In addition, we found that there are 0.5%, 1.1% and 1.0% DAGs out of 39,180 total annotated genes in event-2.4 vs 3.5, event-2.4 vs CK and event-3.5vs CK, respectively (Fig. 3C). Among these DAGs, only 17 genes were uniquely performed in event-2.4 vs 3.5, in which 71% and 33% DAGs are either overlapped with event-2.4 vs CK, or event-3.5 vs CK (Fig. 3C, Supplementary Table S3). We also observed that the numbers of overlapping DAGs were more than that unique DAGs occurred in combination of either transgenic events or transgenic event vs CK (Fig. 3C). These results suggest similar effects between two independent transgenic events on DAGs in ZD958 grown in Beijing, indicating, in fact, that greater effect between two transgenic events and CK was observed than that effect between two transgenic events each other.

Consisted with results of transcriptomes, those of metabolic reprogramming analysis show only 34 DAMs out of 2214 total identified metabolites in event-2.4 vs 3^.^5, while 94 and 97 DAMs were identified in event-2.4 vs CK and event-3.5 vs CK, respectively. In addition, 97% DAMs in event-2.4 vs 3.5 were overlapped with either event-2.4 vs CK or event-3.5 vs CK (Fig. 3D; Supplementary Table S4). These evidences propose that the marginal effects of introducing *HIR* gene cassette on the unfavorable biological process or metabolic pathways into ZD958.

To uncover the regulation of *HIR* gene cassette effects on global mRNA expression and metabolites, we compared the transcriptomes and metabolism between event 3.5 and CK. Our results show that among 374 DAGs, 179 and 195 were down- and up-regulated, respectively (Fig. 3E). We also found that 47 and 50 DAMs were down- and up-regulated, respectively (Fig. 3F). Therefore, introducing *HIR* gene cassette in transgenic event 3.5 leads to even numbers for both down- and up-regulation genes (Fig. 3E). The case is also true for the numbers of DAMs (Fig. 3F).

**Figure 4:**
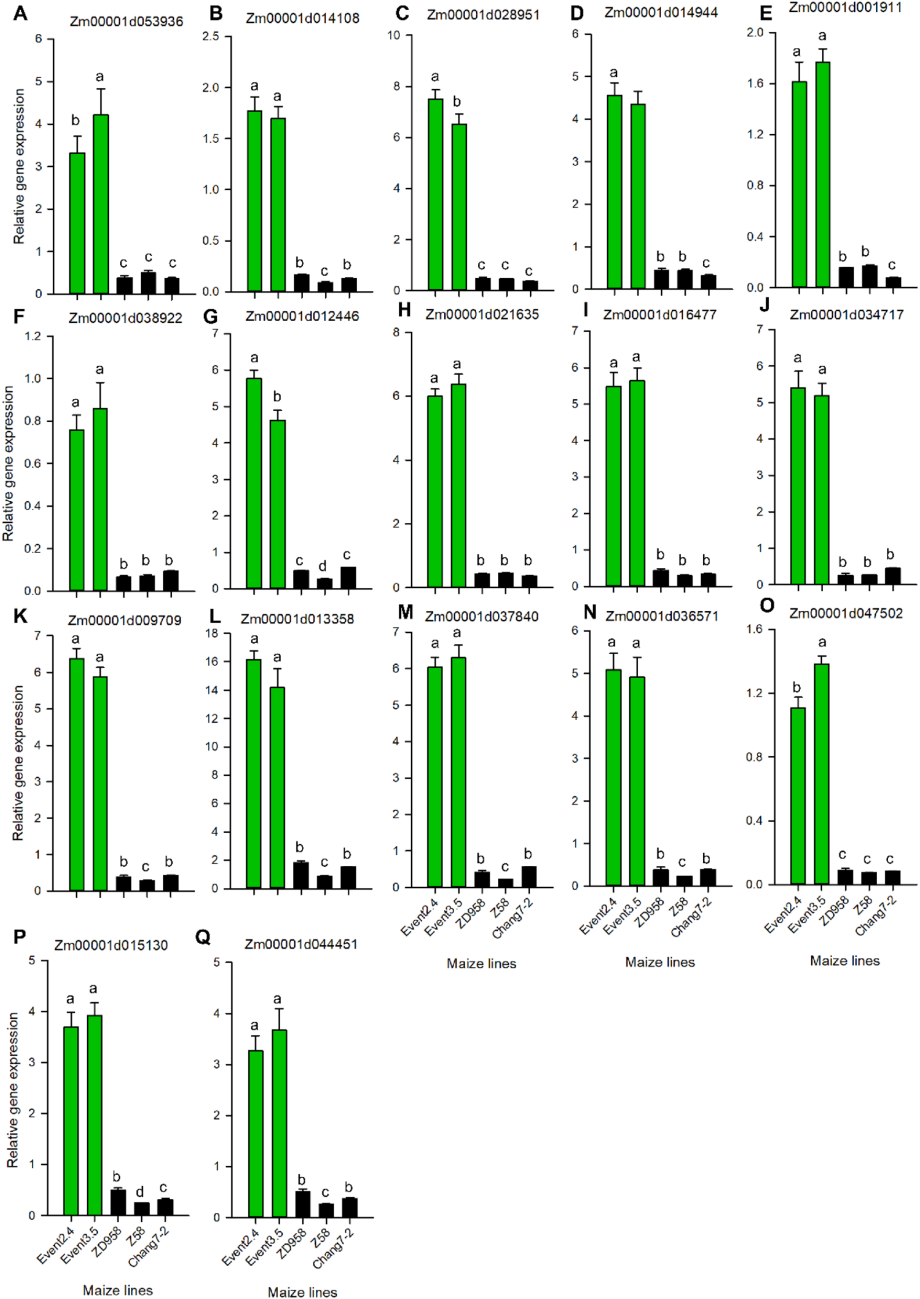
qPCR testing the relative gene expression with top 5% differential expressed genes between two transgenic event (3.5 and 2.4) and other genetic background (Chang7-2, Z58) and non-GM counterpart (ZD958) (**A**–**Q**). Different alphabet letters represent significant differences at *P* < 0.05 based on one-way *ANOVA*. *n* = 6.

**Figure 5:**
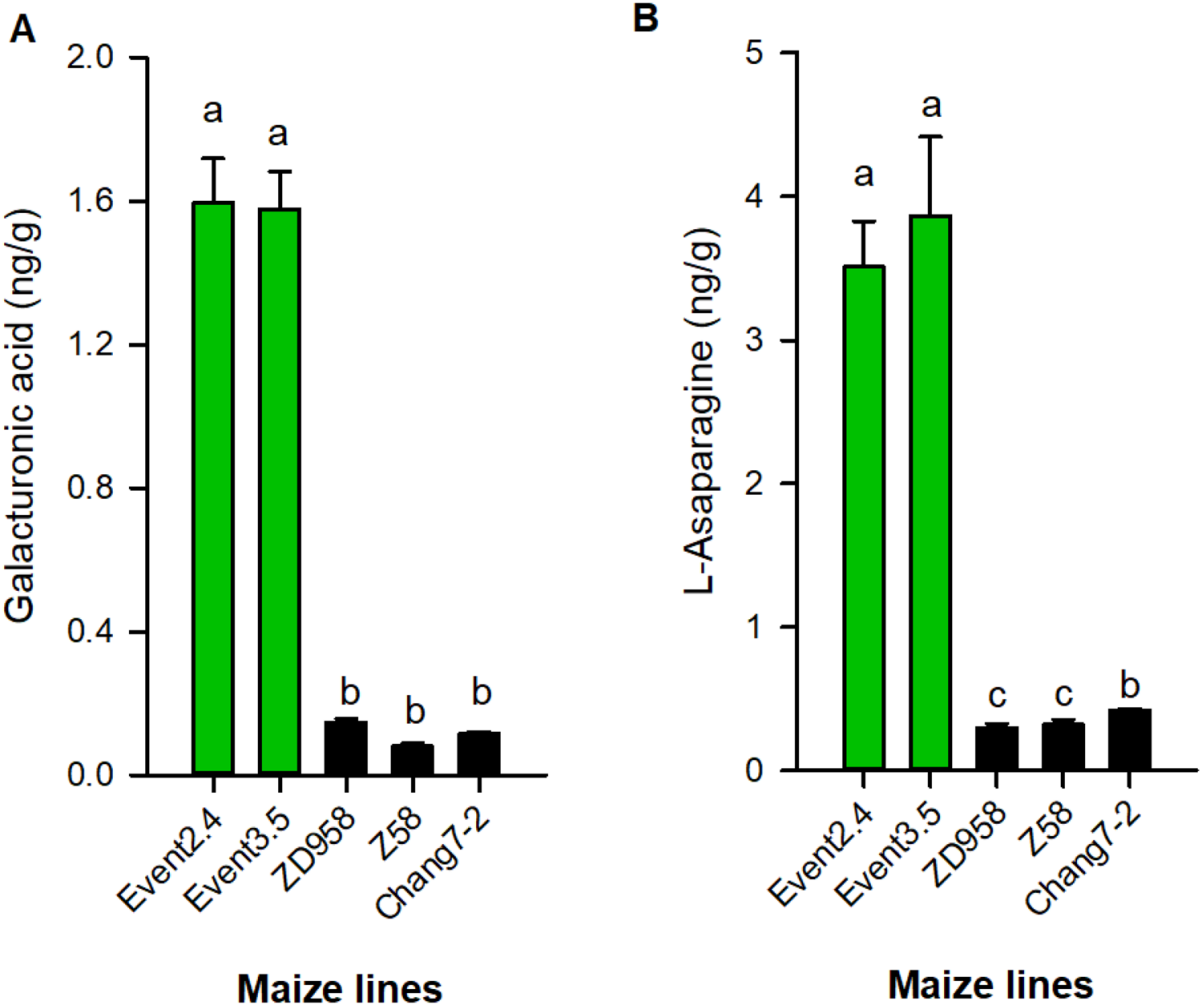
Targeted metabolites measurement to confirm the contents changes induced by *HIR* gene cassette effects using LC–MS/MS with standard curve (a-b). Different alphabet letters represent significant differences at *P* < 0.05 based on one-way *ANOVA*. *n* = 6.

To further confirm the gene expression levels and metabolite contents regulated by the *HIR* gene cassette, we tested top 5% up-regulated genes among 307 DAGs using qPCR and 78 DAMs using targeted metabolites techniques (LC–MS/MS) as listed in Tables 2 and 3. The results confirmed that 17 genes covering different biological pathways were remarkably up-regulated by introducing *HIR* gene cassette, and its expression levels in two transgenic events (events 3.5 and 2.4) for GM-ZD958 were around 3 ~ 20 times higher than its non-GM parents, including Chang 7–2 and Z58 (Fig. 4; Supplementary Datasets 1–6). We also found two overlapped DAMs in the two events, including l-Asparagine and Galacturonic acid (Fig. 5; Table 3). Using targeted-metabolic analysis, we quantitatively determined the amount of these two metabolites and confirmed their high abundance in transgenic lines than in their corresponding non-GM parent maize lines.

**Table 2:** Fold change of top 5% differentially abundant genes (DAGs) between two transgenic events of GM-ZD958 relative to non-GM ZD958 (CK).

Ensembl gene id	GM-ZD958-2.4	GM-ZD958-3.5	*p*-value	Gene description
Zm00001d053936	3.21	2.78	8.84E−06	Polyketide cyclase/dehydrase and lipid transport superfamily protein
Zm00001d014108	3.17	3.27	8.62E−06	Ubiquitin-conjugating enzyme E2 4
Zm00001d028951	3.78	3.65	3.33E−07	Small basic membrane intrinsic protein2a
Zm00001d014944	6.96	2.28	4.72E−06	Lactate dehydrogenase1
Zm00001d001911	5.20	5.37	6.20E−07	ATP-dependent RNA helicase
Zm00001d038922	2.23	2.32	2.98E−06	CRS1/YhbY (CRM) domain-containing protein
Zm00001d012446	5.13	4.78	5.45E−06	V-type proton ATPase subunit a1
Zm00001d021635	6.16	6.25	1.85E−05	Condensin-2 complex subunit H2
Zm00001d016477	7.61	7.83	1.90E−06	Eukaryotic aspartyl protease family protein
Zm00001d034717	6.93	6.59	2.88E−06	*S*-Adenosyl-l-methionine-dependent methyltransferases superfamily protein
Zm00001d009709	6.06	6.63	1.33E−06	Loricrin-related
Zm00001d013358	2.58	2.91	8.79E−06	DEAD-box ATP-dependent RNA helicase 16
Zm00001d037840	5.03	5.87	6.09E−06	Putative leucine-rich repeat receptor-like protein kinase family protein
Zm00001d036571	2.22	2.57	4.22E−06	Heat shock 70 kDa protein 16
Zm00001d047502	3.96	4.22	2.92E−06	DEAD-box ATP-dependent RNA helicase 35
Zm00001d015130	3.54	3.59	1.22E−05	SBP (*S*-ribonuclease binding protein) family protein
Zm00001d044451	4.07	4.14	2.91E−06	RING/FYVE/PHD zinc finger superfamily protein

**Table 3:** Fold change for top 5% DAM in *HIR* gene cassette GM-ZD958 transgenic lines and its nonGM parents (Z58 and Chang7-2), relative to nonGM-ZD958 determined in growth chamber.

Metabolites	Molecular weight	Retention time (min)	GM-ZD958-2.4	GM-ZD958-3.5	Z58	Chang7-2	*p*-value
Galacturonic acid	236.465	8.3	20.727	22.860	1.036	1.095	2.30E−05
l-Asaparagine	228.842	11.678	18.316	19.997	1.039	0.992	2.8E−07

*P*-value represent the significant levels performed by one-way *ANOVA* among different maize lines.

### Experimental effect as a major driving force for both DAGs and DAMs

**Figure 6:**
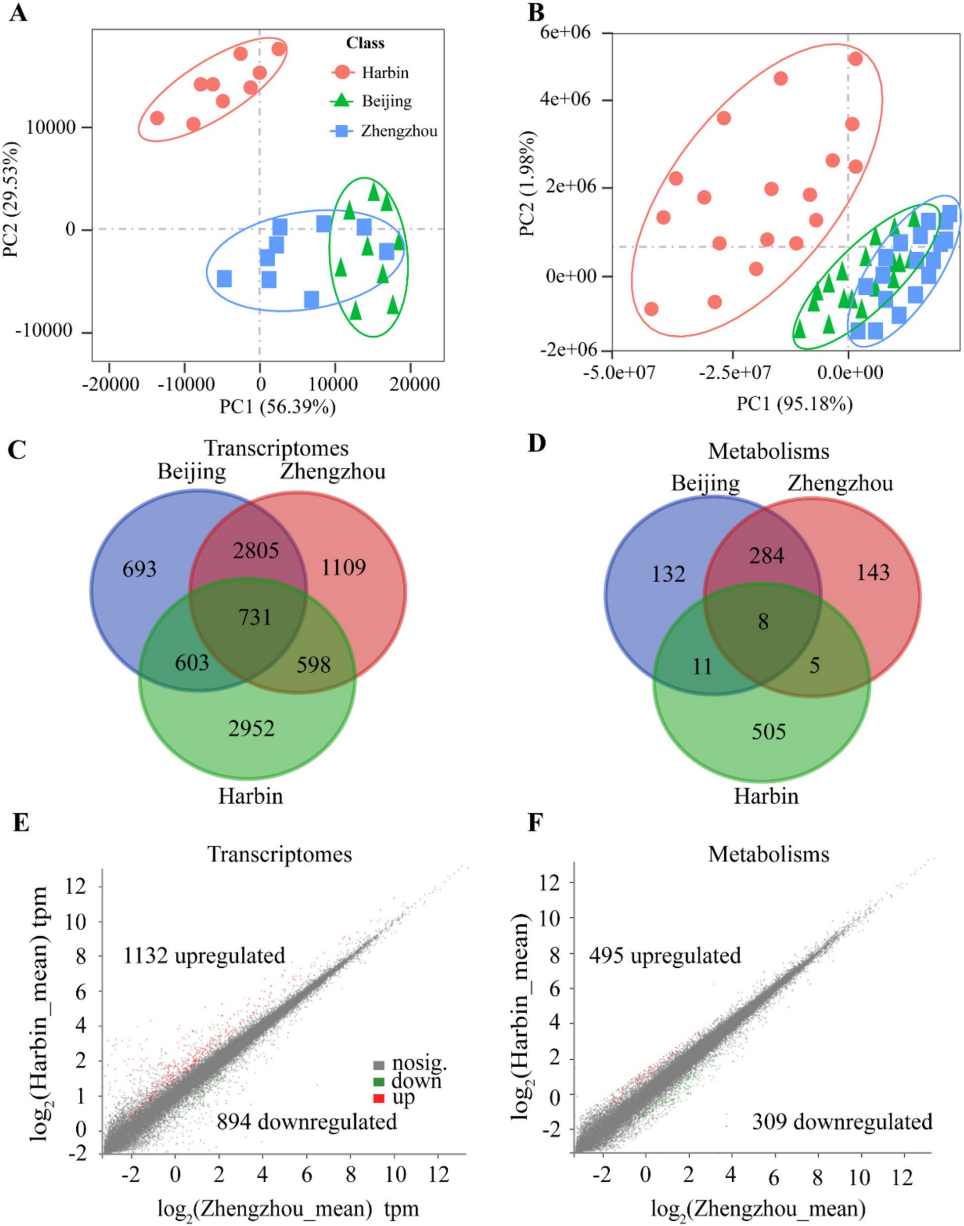
Principal component analysis on different experimental sites for GM-ZD958 across two different transgenic event lines (event-2.4 and 3.5) and non-GM ZD958 (CK). (**A**) Transcriptomes analysis on different experimental sites, i.e., Beijing (E116.3°, N39.7°), Harbin (E125.4°, N44.1°), and Zhengzhou (E113.6°, N34.7°) of China, across different transgenic events and CK; *n* = 9. (**B**) Metabolomics analysis on different experimental sites across different transgenic events and CK. *n* = 18. (**C**,**D**), Effects of different growing environments on differentially abundant genes or metabolites. (**E**,**F**), Comparison on down-/up-regulation of genes or metabolites between Zhengzhou and Harbin.

To test whether different experiments sites have robust effects on DAGs and DAMs, we compared global mRNA expression and the relative abundance of metabolites between GM-ZD958 and non-GM ZD958 (CK) maize lines in three experimental sites of China, i.e., Beijing, Harbin, and Zhengzhou (Fig. 6). Our results show as well that clear separated clusters were observed among the three experimental sites regarding either different transcripts or metabolites, especially between Harbin and Zhengzhou (Fig. 6A,B). We found that 2952 DAGs between the two transgenic events (3.5 and 2.4) and CK in Harbin were not overlapped with either Beijing or Zhengzhou. Similarly, this is true for 505 metabolites measured form Harbin site (Fig. 6C,D; Supplementary Tables S4, S5). The numbers for DAGs induced by environments, for example in Harbin was 8 times higher than that induced solely by the effects of transgenic events compared to CK under growth chamber conditions (Figs. 3C and 6C; Supplementary Table S5), and similar effects were also recorded for DAMs (Figs. 3D and 6D; Supplementary Table S6). Therefore, the above findings suggest that environment has greater effects on the numbers of global molecular profiling than the gene additional effects.

**Figure 7:**
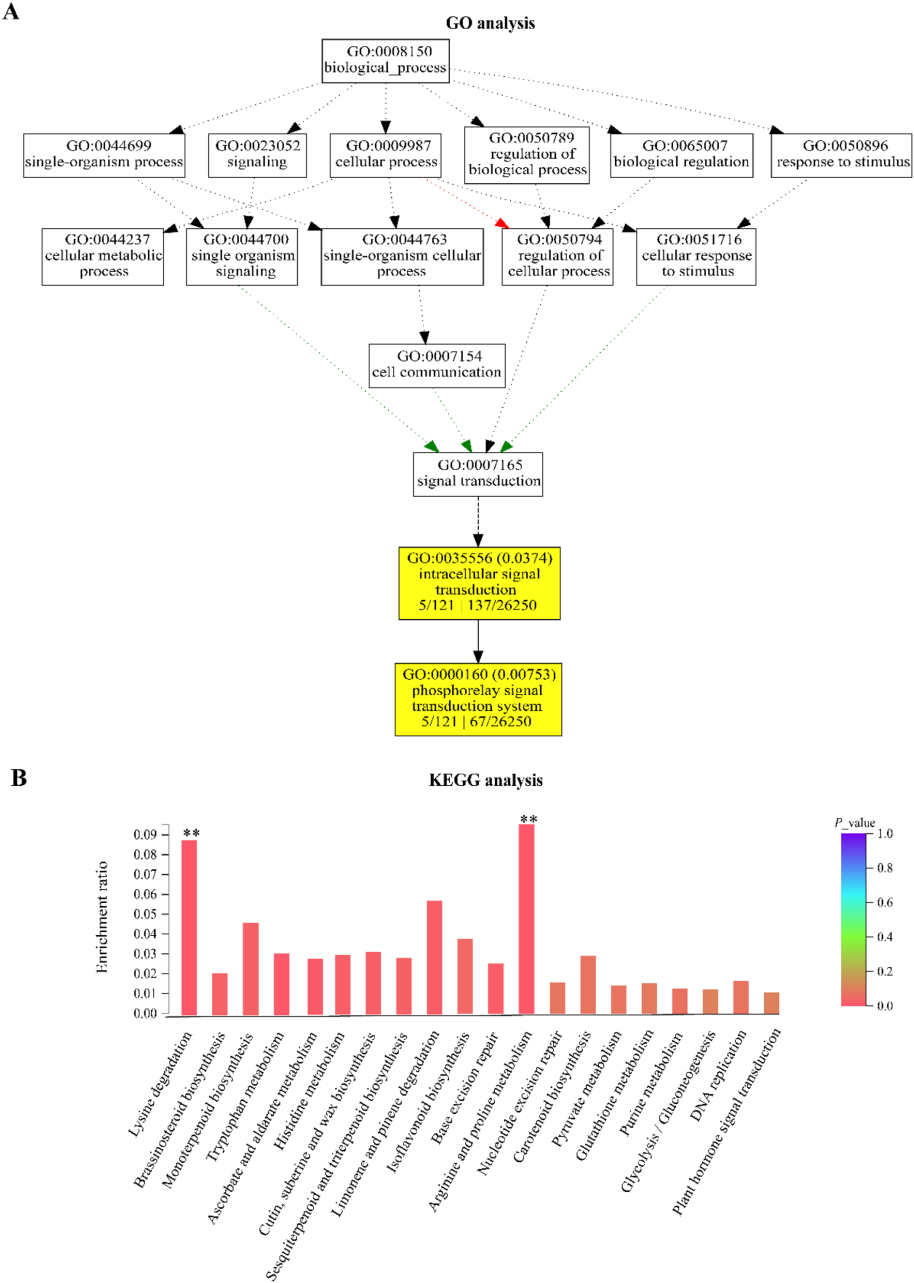
Bioinformatic analysis on different abundant genes in maize grown in Harbin compared to Zhengzhou. The abundance of genes derived from the average across CK and two transgenic events in GM-ZD958 maize lines. (**A**) Gene Ontology (GO) analysis on different abundant genes (DAGs) in maize grown in Harbin compared to Zhengzhou. Enriched biological pathway were highlighted in yellow. (**B**) Kyoto Encyclopedia of Genes and Genomes (KEGG) analysis on DAGs in maize grown in Harbin compared to Zhengzhou. Significantly enriched metabolic pathways were shown symbol “*” and “**”, as indicated *P* value < 0.05 and 0.01, respectively.

We further found that 1132 and 894 genes were down- and up-regulated in Zhengzhou over Harbin, respectively (Fig. 6E). In the same regards, we recorded 495 and 309 down- and up-regulated metabolites in Harbin over Zhengzhou, respectively (Fig. 6F). In addition, we performed GO and KEGG analysis on the DAGs among three experimental sites based on one-way *ANOVA* (*p* < 0.05). Results show that biological pathways in the list of catabolic process, organic substance catabolic process, alpha-amino acid metabolic process, intracellular signal transduction and phophorelay signal transduction system were significantly enriched (Fig. 7A). In addition, many metabolic pathways were enriched such as sesquiterpenoid and triterpenoid biosynthesis, arginine and limonene and pinene degradation, brassinosteriod biosynthesis (Fig. 7B). We further picked up top 5% of DAGs according to *p*-value among three experimental sites based on one-way *ANOVA*, and the list of these DAGs were shown in Table 2. We found most of genes related to abiotic stress inducible proteins, such as *HtfB-2b*, *DnaJ* and *PPR* gene family, and some transcription factors, such as ZIPs, WEB, DNA-binding-TFs. This suggest that environmental effects induce multiple alteration of biological pathways, and the alteration is above the expectation on herbicide and disease response.

### Marginal effects of transgenic HIR gene cassette on biological process

**Figure 8:**
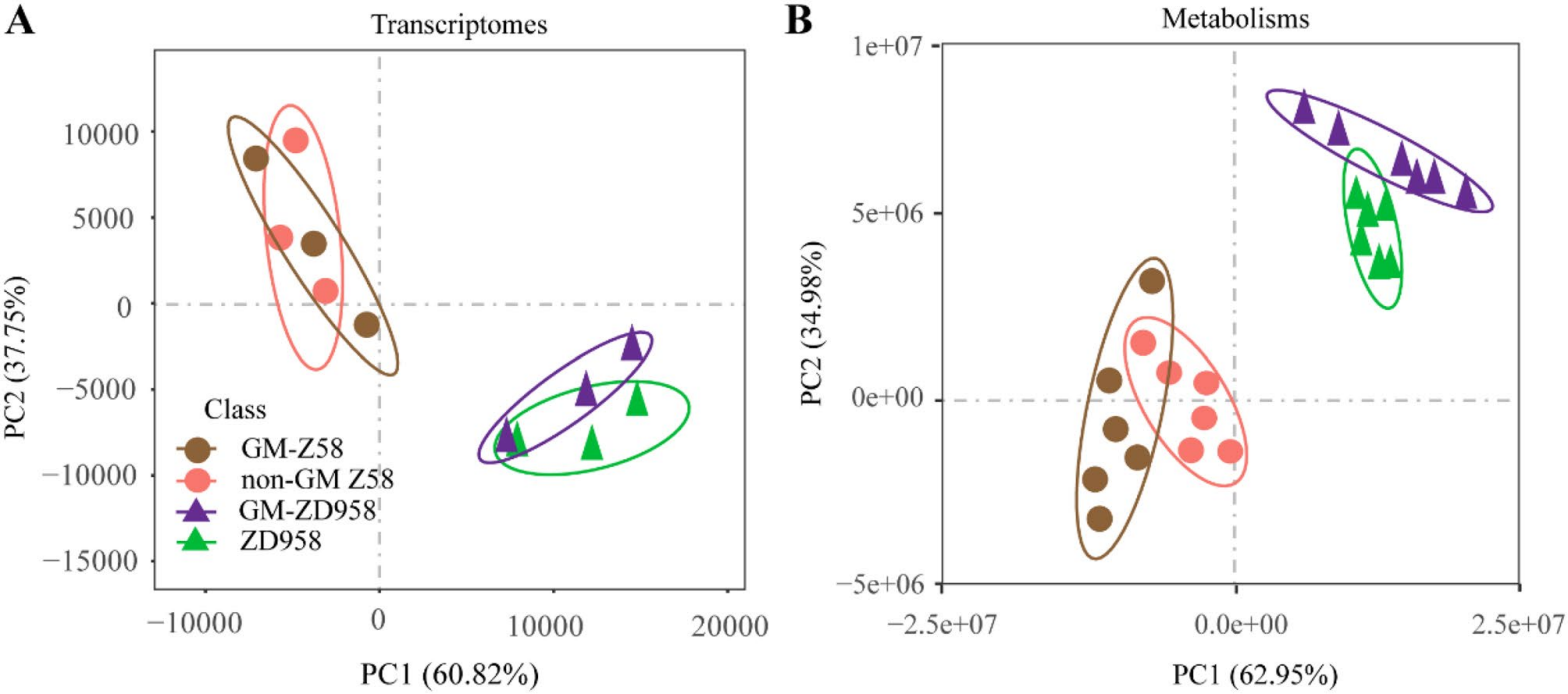
Principal component analysis on GM-ZD958 maize transgenic lines across two transgenic events against different genetic background GM-Z58, Z58, and non-GM ZD958 grown under growth chamber across two transgenic events. (**A**) transcriptomes data; (**B**) metabolic data.

In order to illustrate the effects of transgenic backgrounds on biological gene expression and metabolites, we performed an integrative analysis on transcriptomes and metabolism in GM-Z58 across two transgenic events (event-2.4 and 3.5), non-GM Z58, GM-ZD958 across two transgenic events and non-GM ZD958 (Fig. 8). Results from PCA on relative expression of gene and metabolites, show that the high similarity was observed between transgenic lines harboring *HIR* gene cassette and original transgenic background for both Z58 and ZD958 regardless of transcriptomes or metabolisms (Fig. 8A,B).

## Discussion

With the developments of GM, a variety of transgenic maize plants have been produced with different characteristics including insect resistant-maize and herbicide-tolerant maize. In this study, we introduced a *HIR* gene cassette, which confers herbicide and insect resistance, into a commercial maize variety, Zhengdan958 (ZD958). We presented the first multi-omic analysis of GM-ZD958 maize compared to a non-GM ZD958 maize line. Our analysis at a detailed, in-depth molecular profiling levels shows that marginal effects on the alteration of differential abundant genes and metabolites in GM-ZD958, compared to its non-GM ZD958 maize line.

### Case-study on transgenic events effects on molecular profiling

Unintended effects of GM crops are the main concern of administrators and citizens after their intended effects have been thoroughly investigated. This unintended effects majorly derived from random integration on undesirable genomic-regions during transgenic process such as agrobacteria-mediated transformation^9^. We present the first time transgenic events effects on molecular profiling. Two-independent transgenic events (event-2.4, and event-3.5) were applied, through using same gene constructs to elucidate differential abundant transcripts and metabolites due to transgenic effects and off-target effects (Fig. 3). We identified only 0.5% DAGs that observed between event-2.4 and event-3.5 (Fig. 3C). Interestingly, the numbers of DAGs induced by *HIR* gene cassette effects compared to non-GM ZD958 (CK) account 5% for total annotated genes. In addition, only 17 genes showing no significant difference between two transgenic events but showing significant difference compared to CK (Table 2). However, no significant biological pathways or categories were enriched. We found that around 4% differentially abundant metabolites (DAMs) account for total identified metabolites due to *HIR* gene cassette effects irrespective of off-target effects, which is in line with similar numbers of metabolites as observed in NK603 maize lines^16^. These findings suggest that the off-target and gene effects are equivalent in the derived maize lines from two transgenic integration events *HIR* gene cassette effects.

### Reprogrammed molecular profiling induced by environmental effects

We looked at the combined effects of genetic modification and growing environments by planting two GM-ZD958 across two transgenic event (event 2.4 and 3.5) and non-GM ZD958) in three different ecological regions of China, i.e., Harbin, Zhengzhou, and Beijing. A distinct separation between Harbin with other two ecological regions was observed, however partial overlapping was observed between Zhengzhou and Beijing for both transcriptomics and metabolic analysis. This is very like due to the difference of average surface air temperature during growth season (May to Oct) between three sites, Harbin (17 °C), Beijing (28 °C), and Zhengzhou (30 °C). This geo-climate distribution reprograms maize transcriptome, proteome, and metabolome and even by activating cell death mechanisms leading to organ abortion or entire plant death^17,18^. Indeed, we found 894 up-regulated genes due to relatively low temperature in Harbin compared to normal temperature recorded in Zhengzhou. Among these genes, we found they are significantly enriched in the pathway relating to plants response to abiotic tress, including phosphorelay signal transduction system and Arginine/proline metabolism according to GO and KEGG analysis (Fig. 7A,B). Under low temperature environments, integration of cellular membrane was injured, which leads to overproduction of ROS^19^. As protective mechanism, proline, a major type of antioxidant is accumulated, to remove ROS under low temperature condition^20^. Meanwhile, plants evolve to “switch on” the adaptive response through using this phosphorelay signaling machineries, which enables plants to confers tolerance toward abiotic stress^21^. These findings suggest that the environment was even shown to play a stronger effect in gene expression, and metabolite levels of the GM samples than gene modification by previous publications, which showed similar results for transcriptomics in maize and other species, such as rice, wheat, soybean, and potato^13,22–26^.

### Greater genetic background effect on molecular profiling than gene effects

Many efforts have been made to test if GM crops are substantially equivalent as safe non-GM comparators, following a large body of high-quality compositional data that need to be determined according to principles outlined in the Organization for Economic Cooperation and Development^27^. Our study design further highlights the importance of restricting comparison to the two GM maize (GM-ZD958 and GM-Z58) and two non-GM maize lines (nonGM-ZD958 and nonGM-Z58) and cultivation of the two at the same location and growing environments, when the objective is to evaluate the effect of the GM transformation process. A closed cluster regarding transcriptomes and metabolism in ZD958 maize varieties harboring *HIR* gene cassette and its non-GM ZD958 was observed, while it apart from another background nonGM-Z58 (Fig. 8A,B). This suggests that the molecular variability such as gene expression and metabolites levels in maize varieties harboring *HIR* gene cassette are relatively closed, but yielding different with non-GM comparators. As mentioned above, the gene expression and metabolites were largely altered by growing environments (Fig. 6A,B). We extracted the list of genes and metabolites with no significant differences among three growing environments for each GM/or non-GM maize variety, to minimize the environmental effects on the following analysis.

## Materials and methods

### Plant material and plant growth

In this study, Zhengdan58 (ZD958) is used as a reference hybrid line, representing maize lines in F1 generation from its parent inbred lines, i.e., Zheng58 and Chang7-2. The two parent inbred lines are gifts from Beijing Dabeinong Biotechnology Co., Ltd.. Meanwhile, the GM-ZD958 maize cultivar used in this study were derived from a non-GM inbred line Change 7–2 and a transgenic receptor, Zheng58, by expressing a *HIR* gene cassette suite which consists of two genes, i.e., *EPSPS* and *Cry1Ab*, responsible for the transgenic herbicide (glyphosate) and insect (*Bacillus thuringiensis*) resistance genes^28,29^, respectively. For transgenic ZD958 hybrid line, we conducted two independent transgenic events (3.5 and 2.4) from the same construct harboring the *HIR* gene cassette, to eliminate the random insertion effects on undesirable genomic regions. The plants were grown in the field in three different ecological regions of China, namely, Beijing, Zhengzhou, and Harbin, as well as indoor chambers in Beijing as shown in Table 1.

### RNA extraction and transcriptomes analysis

Total RNA was extracted from the leaves of 40 days old maize lines using TRIzol Reagent according to manufacturer’s instructions (Invitrogen, Ohio, USA)^30^. Genomic DNA was removed using DNase I kit (TaKara, Tokyo, Japan). Quality and purity of RNA were determined by 1% of agarose gels and nano-drop (IMPLEN, California, USA), respectively. RNA-seq transcriptome library was constructed using around 5 μg of total RNA based on NEBNext Ultra RNA Kit (Illumina, San Diego, CA, USA)^31^. Briefly, mRNA was firstly isolated through oligo (dT) beads, and crushed by fragmentation buffer, and then double-stranded cDNA was synthesized using a SuperScript double-stranded cDNA synthesis kit (Invitrogen, CA, USA). Then the synthesized cDNA was subjected to end-repair, phosphorylation and ‘A’ base addition according to Illumina’s library construction protocol. Libraries were size selected for cDNA target fragments of 200–300 bp on 2% Low Range Ultra Agarose followed by PCR amplified using Phusion DNA polymerase (NEB) for 15 PCR cycles. After quantification, paired-end RNA-seq sequencing library was sequenced with the Illumina HiSeq 4000 (2 × 150 bp read length)^31^.

The resulting paired-end reads were trimmed and merged with SeqPrep (https://github.com/jstjohn/SeqPrep). The trimming Illumina paired end reads were quality controlled by Sickle (https://github.com/najoshi/sickle). Then clean reads were separately aligned to reference genome with orientation mode using TopHat (http://tophat.cbcb.umd.edu/, version 2.0.0) software^32^. The mapping criteria of bowtie was as follows: sequencing reads should be uniquely matched to the genome allowing up to 2 mismatches, without insertions or deletions. Then, the regions of gene were expanded following depths of sites and the operon was obtained. In addition, the whole genome was split into multiple 15,000 bp windows that share 5000 bp. Therefore, the new transcribed regions were defined as more than 2 consecutive windows without overlapped region of gene, where at least 2 reads mapped per window in the same orientation.

To identify differentially abundant genes (DAGs) between two different samples, the expression level of each transcript was calculated according to Transcript per million (TPM) method, which considers the gene length for normalization, and is suitable for sequencing protocols where reads sequencing depends on gene length^33^. RSEM (http://deweylab.biostat.wisc.edu/rsem/) was used to quantify gene abundance^34^. R statistical package software EdgeR (Empirical analysis of Digital Gene Expression in R, http://www.bioconductor.org/ packages/2.12/bioc/html/edgeR.html) was utilized for differential expression analysis^35^.

### GO and KEGG analysis

An in-house Perl script was used to perform gene ontologies (GO) annotation based on UniProtKB GOA file (ftp.ebi.ac.uk/pub/databases/GO/goa/UNIPROT/gene_association.goa_uniprot.gz). KOBAS (KEGG Orthology Based Annotation System, v2.0) was used to identify biochemical pathways and to calculate the statistical significance for each pathway^36^.

### Quantitative real time PCR

Real-time PCR (qPCR) was used to validate the differentially abundant genes in different background of maize varieties. Total RNA from maize leaves was isolated using Ultra-Pure RNA Kit (cwbiotech), and complementary DNA was synthesized with ExScript RT Kit (Takara). The amplification reaction conditions were as follows: 95 °C for 3 min followed by 40 cycles of 95 °C for 10 s and 60 °C for 30 s. The primer is listed in Table S1. Actin gene was used as internal control. The relative gene expression level was calculated based on 2^−ΔΔCT^ as described previously^37^. Three biological replicates were performed.

### LC–MS/MS based metabolism and identification

Non-targeted metabolic profiling in different transgenic maize lines were determined based on LC–MS/MS (Q Exactive, Thermo Scientific). ~ 2.5 mg leaves of 30 days-old maize were sampled in 2 mL Eppendorf tube filled with pre-cooled metal beads, and immediately stored in liquid nitrogen. The samples were first extracted with ball mill at 30 Hz for 5 min, and the extracted powder was then dissolved with 1.5 mL methanol/chloroform, and incubated at − 20 °C for 5 h. The mixture was centrifuged at 2000*g* at 4 °C for 10 min, and then was filtered with 0.43 μm organic phase medium (GE Healthcare, 6789-0404). The metabolomics profiling analysis was performed by Metabolon software (Durham, NC, USA). For two identified metabolites, i.e., galacturonic acid and l-asaparagine, the contents of two metabolites were further determined by LC–MS/MS (AB Sciex Qtrap 6,500; SCIEX) with standard curve as described previously^38^. Metabolites were identified by analyzing retention times and mass spectra with those for reference compounds as well as the entries of the mass spectra libraries NIST02 and Golm metabolome database (http://csbdb.mpimp-golm.mpg.de/csbdb/gmd/gmd.html).

### Data analysis

Principal component analysis (PCA) was performed using SYSTAT 11 (Systat Software Inc., CA, USA)^39^. One-way *ANOVA* was performed in combination with modified Tukey’s HSD test to identify the differences in the relative abundance of a given transcript, and the metabolites. Differences at the level *P* < 0.01 were considered statistically significant.

## Supplementary Information

Supplementary Information.

## Data Availability

All data is available in the manuscript or the supplementary materials.
