# Supplementary material for: Evaluation on reprogramed biological processes in transgenic maize varieties using transcriptomics and metabolomics
Source: Sci Rep. 2021 Jan 21;11:2050. doi: 10.1038/s41598-021-81637-2 (PMC7820507; doi:10.1038/s41598-021-81637-2)
Supplement: Supplementary file 1 — Supplementary Information. [file 41598_2021_81637_MOESM1_ESM.docx]

**Supplementary dataset**

**Evaluation on reprogramed biological processes in transgenic maize varieties using transcriptomics and metabolomics**

Wei Fu^1^, Pengyu Zhu^1^, Mingnan Qu^2^, Wang Zhi^1^, Yongjiang Zhang^1^, Feiwu Li^3,*^, Shuifang Zhu^1,*^

^1^Institute of Plant Quarantine, Chinese Academy of Inspection and Quarantine, Beijing 100176, China

^2^National Key Laboratory of Plant Molecular Genetics, CAS Center for Excellence in Molecular Plant Sciences, Shanghai, 200032, China

^3^Institute of Agricultural Quality Standard and Testing Technology, Jilin Academy of Agricultural Sciences, Changchun, Jilin, 130033, China

***Corresponding author**: Feiwu Li & Shuifang Zhu ([lifeiwu3394@sina.com](mailto:lifeiwu3394@sina.com), zhushf0421@163.com)

**Supplementary Tables**

**Table S1.** Primer used in this study for qPCR experiments.

**Table S2.** Quality control of RNA sequencing in combinations of experimental sites, genetic backgrounds, transgenic vectors.

**Table S3.** Overlapping genes between different transgenic events of ZD958 and non-GM ZD958 maize lines grown in Beijing, China.

**Table S4.** Overlapping metabolites between different transgenic events of ZD958 and non-GM ZD958 maize lines grown in Beijing, China.

**Table S5.** Overlapping genes between GM-ZD958 and its parent line as well as non-GM Z58 in growth chamber.

**Table S6.** Overlapping metabolites between GM-ZD958 and its parent line as well as non-GM Z58 in growth chamber.

**Supporting dataset 1.** qRT-PCR cycle number (Ct values) for the differentially expressed genes in different maize lines. Data were used to produce Figure 4.

**Supporting dataset 2.** qRT-PCR cycle number (Ct values) for the differentially expressed genes in different maize lines (continue 1). Data were used to produce Figure 4.

**Supporting dataset 3.** qRT-PCR cycle number (Ct values) for the differentially expressed genes in different maize lines (continue 2). Data were used to produce Figure 4.

**Supporting dataset 4.** qRT-PCR cycle number (Ct values) for the differentially expressed genes in different maize lines (continue 3). Data were used to produce Figure 4.

**Supporting dataset 5.** qRT-PCR cycle number (Ct values) for the differentially expressed genes in different maize lines (continue 4). Data were used to produce Figure 4.

**Supporting dataset 6.** qRT-PCR cycle number (Ct values) for the differentially expressed genes in different maize lines (continue 5). Data were used to produce Figure 4.

**Table S1.** Primer used in this study for qPCR experiments.

| **Maize gene ID** | **Forward primer** | **Reverse primer** | **Product size** |
| --- | --- | --- | --- |
| Zm00001d053936 | GGGTATACCATGCTCGTCCA | GTGGAACAAGAGCACCTCAC | 139 |
| Zm00001d014108 | GAGCGACTACAAGGTGGAGA | AGGGTATGCATCAGGCAGTT | 130 |
| Zm00001d028951 | CCTCCTACAATCCGCTCACC | CGGGCTCCTTTACCCACATT | 166 |
| Zm00001d014944 | TTGCCCATTGCCATTCCTTC | TGGGATGGGATTACGTGTGT | 132 |
| Zm00001d001911 | GAAGGGAGGTTCGTGTTCGG | CGTCTTGAGGTGCTTCGTCT | 174 |
| Zm00001d038922 | AAACCCGACACGCCTTCTC | GGACGAGGAAGAGGAAGAGG | 186 |
| Zm00001d012446 | TCGGCCTACTCCAGTTCAAG | AGTGCAGGTCGAACAGAAGA | 160 |
| Zm00001d021635 | GTGCTTCACGCGTTAGAGTT | CCGGTTTCAGTCCCACATTC | 138 |
| Zm00001d016477 | CATCACCTTTGGGTGTGGTG | GTGTCCAAGCAGTGAGCAAA | 159 |
| Zm00001d034717 | AGAAGAATCGGCAAGGGTCA | CCGTCTGACTTCGTTTCCAC | 140 |
| Zm00001d009709 | GGCTTAGAGACAGTGGCAGA | ACACGTCTGTTGTTGAAGGC | 175 |
| Zm00001d013358 | AGGAAGAAGTGGAGGTGAGC | ACCACATCCTTCCCTTCCAG | 139 |
| Zm00001d037840 | ACCAGCGTCGAATTGAACAG | ACAACAGCTGTCATCTGGGA | 139 |
| Zm00001d036571 | GATGGCAAGTACTGCACGTT | ACACACACGAGACCTCACAT | 149 |
| Zm00001d047502 | CTGGTGGCTACTGATGTTGC | TTGCTATGCCAGTCTTCCCA | 142 |
| Zm00001d015130 | CGGTGAACAGGAAGAGCAAG | TTCACGACCGACTGGTTGTA | 103 |
| Zm00001d044451 | TCACACTGCACAACATGCAA | TATAGCTGCGATGGTGGCTT | 147 |

**Table S2.** Quality control of RNA sequencing in combinations of experimental sites, genetic backgrounds, transgenic vectors.

| **Sample** | **Raw Reads** | **Clean Reads** | **Clean Reads Rate (%)** | **Mapped Reads** | **Mapped Rate (%)** | **Uniquely Mapped Reads** | **Uniquely Mapped Rate (%)** | **Exon** | **Intron** | **Intergenic** |
| --- | --- | --- | --- | --- | --- | --- | --- | --- | --- | --- |
| DT_D_2.4_1 | 45,162,026 | 44,368,328 | 98.24 | 40863700 | 92.10 | 38946853 | 95.31 | 96.93% | 1.42% | 1.63% |
| DT_D_2.4_2 | 49,284,512 | 47,988,680 | 97.37 | 44721762 | 93.19 | 42876730 | 95.87 | 96.10% | 2.00% | 1.89% |
| DT_D_2.4_3 | 46,932,842 | 45,735,862 | 97.45 | 41343632 | 90.40 | 39161043 | 94.72 | 95.97% | 1.99% | 2.03% |
| DT_D_3.5_1 | 47,233,176 | 46,357,682 | 98.15 | 42675427 | 92.06 | 40647202 | 95.25 | 96.78% | 1.56% | 1.65% |
| DT_D_3.5_2 | 45,612,124 | 44,677,962 | 97.95 | 41109030 | 92.01 | 39140261 | 95.21 | 96.93% | 1.44% | 1.62% |
| DT_D_3.5_3 | 47,301,282 | 46,396,662 | 98.09 | 42652023 | 91.93 | 40698330 | 95.42 | 96.06% | 2.03% | 1.90% |
| DT_D_ZD958-1 | 49,003,870 | 47,617,920 | 97.17 | 43657634 | 91.68 | 41782765 | 95.71 | 95.61% | 2.37% | 2.00% |
| DT_D_ZD958-2 | 49,624,812 | 47,911,496 | 96.55 | 43937795 | 91.71 | 42036395 | 95.67 | 96.06% | 1.94% | 1.99% |
| DT_D_ZD958-3 | 47,160,058 | 46,154,884 | 97.87 | 42395380 | 91.85 | 40531998 | 95.60 | 96.48% | 1.72% | 1.79% |
| HB_D_2.4_1 | 49,240,978 | 47,949,442 | 97.38 | 44127482 | 92.03 | 42362511 | 96.00 | 96.85% | 1.38% | 1.75% |
| HB_D_2.4_2 | 47,859,140 | 46,388,310 | 96.93 | 42448667 | 91.51 | 40342080 | 95.04 | 97.32% | 0.93% | 1.74% |
| HB_D_2.4_3 | 46,724,266 | 45,478,454 | 97.33 | 41719908 | 91.74 | 40007132 | 95.89 | 95.92% | 2.18% | 1.89% |
| HB_D_3.5_1 | 45,483,952 | 44,789,726 | 98.47 | 40726138 | 90.93 | 39003123 | 95.77 | 95.84% | 2.02% | 2.13% |
| HB_D_3.5_2 | 46,203,684 | 44,865,000 | 97.10 | 41018543 | 91.43 | 39325699 | 95.87 | 96.33% | 1.84% | 1.82% |
| HB_D_3.5_3 | 47,067,982 | 45,609,746 | 96.90 | 41852170 | 91.76 | 39985295 | 95.54 | 96.46% | 1.67% | 1.86% |
| HB_D_ZD958_1 | 47,776,912 | 45,924,064 | 96.12 | 41931175 | 91.31 | 40029657 | 95.47 | 96.01% | 1.94% | 2.03% |
| HB_D_ZD958_2 | 47,779,836 | 46,808,964 | 97.97 | 43232281 | 92.36 | 41385168 | 95.73 | 96.38% | 1.80% | 1.80% |
| HB_D_ZD958_3 | 47,356,242 | 46,346,258 | 97.87 | 42637601 | 92.00 | 40848004 | 95.80 | 96.28% | 1.82% | 1.88% |
| WS_D_2.4_1 | 45,705,176 | 44,566,680 | 97.51 | 40789918 | 91.53 | 39059803 | 95.76 | 96.48% | 1.63% | 1.87% |
| WS_D_2.4_2 | 46,806,340 | 45,669,934 | 97.57 | 41916938 | 91.78 | 40043330 | 95.53 | 96.62% | 1.72% | 1.64% |
| WS_D_2.4_3 | 47,414,458 | 45,978,464 | 96.97 | 42200357 | 91.78 | 40155238 | 95.15 | 96.53% | 1.70% | 1.75% |
| WS_D_3.5_1 | 45,110,840 | 44,186,262 | 97.95 | 40469617 | 91.59 | 38659174 | 95.53 | 96.12% | 1.94% | 1.92% |
| WS_D_3.5_2 | 46,518,128 | 45,378,922 | 97.55 | 41601809 | 91.68 | 39539513 | 95.04 | 96.06% | 1.93% | 1.99% |
| WS_D_3.5_3 | 48,771,092 | 47,849,394 | 98.11 | 43614052 | 91.15 | 41700465 | 95.61 | 95.40% | 2.33% | 2.25% |
| WS_D_ZD958_1 | 46,747,484 | 45,329,604 | 96.97 | 41448806 | 91.44 | 39660451 | 95.69 | 95.47% | 2.44% | 2.08% |
| WS_D_ZD958_2 | 45,841,222 | 45,040,980 | 98.25 | 41438398 | 92.00 | 39436818 | 95.17 | 96.76% | 1.60% | 1.62% |
| WS_D_ZD958_3 | 46,613,540 | 45,292,150 | 97.17 | 41377218 | 91.36 | 39473525 | 95.40 | 95.80% | 2.10% | 2.09% |
| XZ_D_2.4_1 | 48,045,596 | 47,235,540 | 98.31 | 43249010 | 91.56 | 41156191 | 95.16 | 95.68% | 2.23% | 2.07% |
| XZ_D_2.4_2 | 47,980,542 | 47,256,534 | 98.49 | 42862729 | 90.70 | 41059385 | 95.79 | 95.91% | 1.87% | 2.21% |
| XZ_D_2.4_3 | 48,148,580 | 47,291,830 | 98.22 | 43301875 | 91.56 | 41545383 | 95.94 | 95.57% | 2.30% | 2.11% |
| XZ_D_3.5_1 | 49,143,066 | 46,995,758 | 95.63 | 42775985 | 91.02 | 40850346 | 95.50 | 95.99% | 1.72% | 2.27% |
| XZ_D_3.5_2 | 47,504,732 | 46,579,202 | 98.05 | 42832003 | 91.96 | 41146099 | 96.06 | 96.27% | 1.88% | 1.83% |
| XZ_D_3.5_3 | 46,677,870 | 45,658,102 | 97.81 | 41671747 | 91.27 | 39757330 | 95.41 | 95.99% | 1.86% | 2.14% |
| XZ_D_ZD958_1 | 48,077,996 | 47,079,590 | 97.92 | 42898626 | 91.12 | 40961609 | 95.48 | 96.22% | 1.74% | 2.02% |
| XZ_D_ZD958_2 | 48,637,802 | 47,827,484 | 98.33 | 43960392 | 91.91 | 41954486 | 95.44 | 96.32% | 1.91% | 1.76% |
| XZ_D_ZD958_3 | 47,568,458 | 46,401,068 | 97.55 | 42484419 | 91.56 | 40461288 | 95.24 | 96.72% | 1.41% | 1.86% |
| WS_Z58_3.5_1 | 47,805,977 | 46,549,020 | 94.45 | 43380109.1 | 90.39 | 41590428.1 | 93.00 | 93.22% | 1.94% | 1.83% |
| WS_Z58_3.5_2 | 45,816,181 | 44,966,952 | 95.21 | 41395164.2 | 89.30 | 39427785.9 | 92.39 | 93.88% | 1.51% | 1.60% |
| WS_Z58_3.5_3 | 47,533,754 | 46,189,382 | 94.25 | 42347905 | 88.93 | 40529282.1 | 92.83 | 92.74% | 2.30% | 1.94% |
| WS_Z58_ck_1 | 46,423,366 | 44,996,661 | 94.02 | 41175207 | 88.76 | 39131817.6 | 92.19 | 94.40% | 0.90% | 1.69% |
| WS_Z58_ck_2 | 44,817,573 | 43,519,050 | 94.19 | 39787986.7 | 88.69 | 38145928 | 93.00 | 93.44% | 1.78% | 1.77% |
| WS_Z58_ck_3 | 46,343,605 | 44,546,342 | 93.24 | 40673239.8 | 88.57 | 38828767.3 | 92.60 | 93.13% | 1.88% | 1.97% |
| WS_Chang7-2_3.5_1 | 44,334,021 | 43,229,680 | 94.58 | 39566220.5 | 88.78 | 37888008.9 | 92.89 | 93.59% | 1.58% | 1.81% |
| WS_Chang7-2_3.5_2 | 45,992,024 | 44,599,110 | 94.06 | 40934346.3 | 89.03 | 38950580.9 | 92.30 | 93.63% | 1.65% | 1.70% |
| WS_Chang7-2_3.5_3 | 45,122,584 | 44,017,554 | 94.62 | 40353754.7 | 88.93 | 38353327.6 | 92.19 | 93.18% | 1.87% | 1.93% |
| WS_Chang7-2_ck_1 | 45,345,059 | 43,969,716 | 94.06 | 40205341.8 | 88.70 | 38470637.5 | 92.81 | 92.61% | 2.37% | 2.02% |
| WS_Chang7-2_ck_2 | 45,215,134 | 43,933,386 | 94.25 | 40135901.5 | 88.62 | 38289319.3 | 92.54 | 92.93% | 2.04% | 2.03% |
| WS_Chang7-2_ck_3 | 46,704,123 | 45,873,075 | 95.27 | 42002818.8 | 88.81 | 40299021.5 | 93.07 | 92.70% | 2.23% | 2.05% |
| Mean | 46,991,125 | 45,820,351 | 97 | 41,997,963 | 91 | 40,117,408 | 94.80 | 95.49% | 1.84% | 1.90% |

**Note:** DT_D: represents field experiments in Beijing; 2.4 and 3.5: two transgenic events; HB: Harbin experimental site; WS: growth chamber experiments; XZ: Zhengzhou experimental site.

**Table S3.** Overlapping genes between different transgenic events of ZD958 and non-GM ZD958 maize lines grown in Beijing, China.

| **Names** | **Total** | **Elements** |
| --- | --- | --- |
| Event24-35 Event24 vs mock Event35 vs mock | 27 | Zm00001d037060 Zm00001d049244 Zm00001d018516 Zm00001d020133 Zm00001d043625 Zm00001d010343 Zm00001d029059 Zm00001d001982 Zm00001d016255 Zm00001d037205 Zm00001d044975 Zm00001d004557 Zm00001d037158 Zm00001d014752 Zm00001d048064 Zm00001d040681 Zm00001d008764 Zm00001d047720 Zm00001d033827 Zm00001d009034 Zm00001d041191 Zm00001d026094 Zm00001d005754 Zm00001d009826 Zm00001d036551 Zm00001d018613 Zm00001d040276 |
| Event24-35 Event24 vs mock | 136 | Zm00001d014146 Zm00001d045459 Zm00001d037118 Zm00001d023347 Zm00001d006402 Zm00001d018393 Zm00001d012478 Zm00001d026187 Zm00001d034543 Zm00001d049768 Zm00001d039513 Zm00001d015204 Zm00001d020670 Zm00001d047519 Zm00001d007827 Zm00001d027399 Zm00001d009394 Zm00001d039715 Zm00001d049201 Zm00001d024734 Zm00001d004439 Zm00001d048208 Zm00001d014952 Zm00001d042108 Zm00001d016269 Zm00001d022252 Zm00001d012500 Zm00001d037471 Zm00001d006756 Zm00001d015206 Zm00001d002172 Zm00001d029764 Zm00001d017534 Zm00001d002616 Zm00001d008850 Zm00001d016698 Zm00001d009582 Zm00001d047854 Zm00001d021122 Zm00001d030038 Zm00001d018414 Zm00001d011919 Zm00001d015325 Zm00001d027957 Zm00001d041481 Zm00001d025319 Zm00001d020042 Zm00001d026282 Zm00001d011710 Zm00001d021942 Zm00001d017671 Zm00001d053815 Zm00001d045353 Zm00001d005039 Zm00001d017331 Zm00001d005779 Zm00001d037607 Zm00001d003668 Zm00001d039468 Zm00001d002630 Zm00001d012383 Zm00001d007909 Zm00001d011058 Zm00001d046940 Zm00001d045404 Zm00001d003969 Zm00001d013082 Zm00001d037708 Zm00001d037288 Zm00001d046827 Zm00001d029723 Zm00001d046629 Zm00001d008903 Zm00001d026191 Zm00001d036545 Zm00001d001857 Zm00001d038372 Zm00001d053569 Zm00001d042049 Zm00001d007027 Zm00001d008495 Zm00001d024890 Zm00001d017817 Zm00001d031258 Zm00001d031484 Zm00001d027684 Zm00001d041762 Zm00001d046391 Zm00001d021946 Zm00001d052340 Zm00001d030105 Zm00001d013858 Zm00001d023396 Zm00001d040638 Zm00001d028423 Zm00001d044250 Zm00001d039902 Zm00001d005894 Zm00001d014680 Zm00001d034031 Zm00001d026061 Zm00001d029763 Zm00001d034444 Zm00001d033507 Zm00001d032459 Zm00001d033730 Zm00001d011561 Zm00001d005978 Zm00001d042911 Zm00001d047392 Zm00001d015475 Zm00001d019311 Zm00001d039140 Zm00001d038923 Zm00001d026597 Zm00001d017927 Zm00001d013419 Zm00001d024442 Zm00001d022396 Zm00001d015434 Zm00001d031580 Zm00001d012824 Zm00001d032973 Zm00001d027345 Zm00001d002006 Zm00001d045314 Zm00001d018261 Zm00001d030945 Zm00001d018342 Zm00001d029107 Zm00001d018122 Zm00001d029468 Zm00001d039514 Zm00001d043849 Zm00001d038486 Zm00001d027483 |
| Event24-35 Event35 vs mock | 49 | Zm00001d019750 Zm00001d049195 Zm00001d015845 Zm00001d018730 Zm00001d020640 Zm00001d049176 Zm00001d047265 Zm00001d003765 Zm00001d033738 Zm00001d020679 Zm00001d028975 Zm00001d001818 Zm00001d022211 Zm00001d019980 Zm00001d030392 Zm00001d019788 Zm00001d042258 Zm00001d036666 Zm00001d038924 Zm00001d035642 Zm00001d030982 Zm00001d048108 Zm00001d044899 Zm00001d004747 Zm00001d017124 Zm00001d014763 Zm00001d024770 Zm00001d017911 Zm00001d003297 Zm00001d036706 Zm00001d004190 Zm00001d040091 Zm00001d003755 Zm00001d019993 Zm00001d004138 Zm00001d043854 Zm00001d038267 Zm00001d048212 Zm00001d033339 Zm00001d032263 Zm00001d045553 Zm00001d038317 Zm00001d032261 Zm00001d030017 Zm00001d048453 Zm00001d042972 Zm00001d018894 Zm00001d048666 Zm00001d033794 |
| Event24 vs mock Event35 vs mock | 280 | Zm00001d007445 Zm00001d019660 Zm00001d012819 Zm00001d006880 Zm00001d013812 Zm00001d034249 Zm00001d013872 Zm00001d017711 Zm00001d006179 Zm00001d015581 id Zm00001d013290 Zm00001d044436 Zm00001d052667 Zm00001d023880 Zm00001d013721 Zm00001d053753 Zm00001d021906 Zm00001d041067 Zm00001d034022 Zm00001d042090 Zm00001d012083 Zm00001d011334 Zm00001d009707 Zm00001d016721 Zm00001d049361 Zm00001d027674 Zm00001d035447 Zm00001d050747 Zm00001d039300 Zm00001d014919 Zm00001d030262 Zm00001d030859 Zm00001d041174 Zm00001d024630 Zm00001d029594 Zm00001d039116 Zm00001d021444 Zm00001d026051 Zm00001d015181 Zm00001d033180 Zm00001d007070 Zm00001d042837 Zm00001d043782 Zm00001d013045 Zm00001d016798 Zm00001d017514 Zm00001d052519 Zm00001d009843 Zm00001d021672 Zm00001d011735 Zm00001d052845 Zm00001d004894 Zm00001d006255 Zm00001d025950 Zm00001d042665 Zm00001d034145 Zm00001d023339 Zm00001d023677 Zm00001d014863 Zm00001d021494 Zm00001d050168 Zm00001d018839 Zm00001d035212 Zm00001d008808 Zm00001d007025 Zm00001d042314 Zm00001d032480 Zm00001d047013 Zm00001d038840 Zm00001d051362 Zm00001d044539 Zm00001d025770 Zm00001d036877 Zm00001d046937 Zm00001d015202 Zm00001d034745 Zm00001d020851 Zm00001d021967 Zm00001d043513 Zm00001d007857 Zm00001d027611 Zm00001d009148 Zm00001d018157 Zm00001d018529 Zm00001d027937 Zm00001d035064 Zm00001d024094 Zm00001d045356 Zm00001d017417 Zm00001d048495 Zm00001d045567 Zm00001d007753 Zm00001d038684 Zm00001d008222 Zm00001d028625 Zm00001d013819 Zm00001d043458 Zm00001d038761 Zm00001d033296 Zm00001d018576 Zm00001d040589 Zm00001d027499 Zm00001d010465 Zm00001d020013 Zm00001d038487 Zm00001d021249 Zm00001d033275 Zm00001d007231 Zm00001d051511 Zm00001d002673 Zm00001d002972 Zm00001d017069 Zm00001d031736 Zm00001d053266 Zm00001d034601 Zm00001d024597 Zm00001d045494 Zm00001d027436 Zm00001d002028 Zm00001d028273 Zm00001d010321 Zm00001d046591 Zm00001d003003 Zm00001d009716 Zm00001d040002 Zm00001d037736 Zm00001d042767 Zm00001d033307 Zm00001d034900 Zm00001d002814 Zm00001d036804 Zm00001d027838 Zm00001d044054 Zm00001d040269 Zm00001d049753 Zm00001d034241 Zm00001d022439 Zm00001d030772 Zm00001d049186 Zm00001d046510 Zm00001d039697 Zm00001d017880 Zm00001d033402 Zm00001d039658 Zm00001d009556 Zm00001d018412 Zm00001d023267 Zm00001d007491 Zm00001d019147 Zm00001d031292 Zm00001d038274 Zm00001d005161 Zm00001d017291 Zm00001d026606 Zm00001d044918 Zm00001d052595 Zm00001d021173 Zm00001d004331 Zm00001d022469 Zm00001d019825 Zm00001d048736 Zm00001d034412 Zm00001d027967 Zm00001d028591 Zm00001d033292 Zm00001d052184 Zm00001d030910 Zm00001d040659 Zm00001d033337 Zm00001d002708 Zm00001d025347 Zm00001d051959 Zm00001d002799 Zm00001d018229 Zm00001d028548 Zm00001d033315 Zm00001d046753 Zm00001d020586 Zm00001d040235 Zm00001d002285 Zm00001d034670 Zm00001d033300 Zm00001d026532 Zm00001d004413 Zm00001d043735 Zm00001d026559 Zm00001d046054 Zm00001d048324 Zm00001d002597 Zm00001d008812 Zm00001d007294 Zm00001d043218 Zm00001d023437 Zm00001d002423 Zm00001d032746 Zm00001d027440 Zm00001d043298 Zm00001d040084 Zm00001d024927 Zm00001d006483 Zm00001d034082 Zm00001d015720 Zm00001d030995 Zm00001d003358 Zm00001d043047 Zm00001d027405 Zm00001d014632 Zm00001d023322 Zm00001d007473 Zm00001d048263 Zm00001d010159 Zm00001d043361 Zm00001d048593 Zm00001d008733 Zm00001d054066 Zm00001d028245 Zm00001d021892 Zm00001d028240 Zm00001d002038 Zm00001d037208 Zm00001d033323 Zm00001d025834 Zm00001d025423 Zm00001d036489 Zm00001d051520 Zm00001d029645 Zm00001d033039 Zm00001d018810 Zm00001d013941 Zm00001d018857 Zm00001d032728 Zm00001d029141 Zm00001d035881 Zm00001d009057 Zm00001d040517 Zm00001d013259 Zm00001d042453 Zm00001d052089 Zm00001d012417 Zm00001d015638 Zm00001d028471 Zm00001d020629 Zm00001d050121 Zm00001d045340 Zm00001d029919 Zm00001d009591 Zm00001d012770 Zm00001d017438 Zm00001d023646 Zm00001d050961 Zm00001d023781 Zm00001d049113 Zm00001d048494 Zm00001d028365 Zm00001d023573 Zm00001d045366 Zm00001d025707 Zm00001d033340 Zm00001d040450 Zm00001d017281 Zm00001d015377 Zm00001d040878 Zm00001d005297 Zm00001d015385 Zm00001d006302 Zm00001d028806 Zm00001d020434 Zm00001d038176 Zm00001d019852 Zm00001d049715 Zm00001d039119 Zm00001d048035 Zm00001d011214 Zm00001d017888 Zm00001d013554 Zm00001d049789 Zm00001d028296 Zm00001d018111 |
| Event 24 vs Event 35 | 17 | Zm00001d029304 Zm00001d028455 Zm00001d006499 Zm00001d037748 Zm00001d013101 Zm00001d026528 Zm00001d043036 Zm00001d013816 Zm00001d024768 Zm00001d037757 Zm00001d014518 Zm00001d038484 Zm00001d045839 Zm00001d029749 Zm00001d050866 Zm00001d011157 Zm00001d041583 |
| Event24 vs mock | 23 | Zm00001d019387 Zm00001d013384 Zm00001d020384 Zm00001d042753 Zm00001d004664 Zm00001d040693 Zm00001d021372 Zm00001d052123 Zm00001d009799 Zm00001d029745 Zm00001d042328 Zm00001d020002 Zm00001d045080 Zm00001d020741 Zm00001d042107 Zm00001d025044 Zm00001d050642 Zm00001d024231 Zm00001d018069 Zm00001d053295 Zm00001d033256 Zm00001d028171 Zm00001d032175 |
| Event35 vs mock | 18 | Zm00001d036616 Zm00001d038306 Zm00001d006204 Zm00001d044898 Zm00001d008582 Zm00001d002966 Zm00001d040656 Zm00001d007569 Zm00001d027388 Zm00001d015404 Zm00001d033471 Zm00001d016732 Zm00001d052157 Zm00001d048567 Zm00001d027766 Zm00001d009810 Zm00001d030985 Zm00001d019643 |

**Table S4.** Overlapping metabolites between different transgenic events of ZD958 and non-GM ZD958 maize lines grown in Beijing, China.

| **Names** | **Total** | **Elements** |
| --- | --- | --- |
| Event24-35 Event24 vs mock Event35 vs mock | 13 | Hippuric acid D-Erythrose 4-phosphate D-gluconate 3.alpha.-Mannobiose Diosmetin sn-Glycerol 1-phosphate Metaraminol DL-2-Aminooctanoic acid 1-Palmitoyl-2-oleoyl-phosphatidylglycerol D-Aspartic acid 4-acetamidobutanoate D-Pipecolinic acid D-Mannose 1-phosphate |
| Event24-35 Event24 vs mock | 9 | Uridine diphosphate glucose(UDP-D-Glucose) DL-3-Hydroxybutyric acid 4-Aminobutyric acid (S)-2-Hydroxyglutarate D-Ribose 5-phosphate N-Acetyl-D-Glucosamine 6-Phosphate Nicotinate L-Aspartate N-Acetyl-L-glutamic acid |
| Event24-35 Event35 vs mock | 11 | N-Acetyl-D-lactosamine Thymidine 3-Aminobenzoic acid Arg-Ala Trehalose Uridine Cyanidin 3-glucoside cation Hypoxanthine Val-Ile Choline Eicosapentaenoic acid |
| Event24 vs mock Event35 vs mock | 65 | Maltitol (3-Carboxypropyl)trimethylammonium cation Kaempferol 5(S)-HETE 1-Palmitoyl-2-linoleoyl-sn-glycero-3-phosphate Pectin (Galacturonic acid) Glycerol 3-phosphate Xylitol S-Methyl-5'-thioadenosine Ala-Gly Dimethylglycine Phthalic acid Mono-2-ethylhexyl Ester Acetylcarnitine Acetylvalerenolic acid (4Z,7Z,10Z,13Z,16Z,19Z)-4,7,10,13,1 6,19-Docosahexaenoic acid Lys-Pro Guanosine Sucrose D-Quinovose Pargyline Phosphorylcholine Fludrocortisone acetate D-Tagatose L-Serine Diosmin Jasmine lactone Adenosine gamma-L-Glutamyl-L-glutamic acid Perillyl alcohol Adenine L-Lysine 1,2-dioleoyl-sn-glycero-3-phosphatidylcholine Nomilin 2-Oxoadipic acid 3',5'-Cyclic guanosine monophosphate D-Galactarate D-Lyxose 2-Hydroxyadenine N-epsilon,N-epsilon,N-epsilon-Trimethyllysine 13(S)-HOTrE Arbutin 1-Palmitoyl-sn-glycero-3-phosphocholine Quinic acid L-Threonate Kynurenic acid Larixinic Acid Fluconazole Hieracin Sunitinib 1-Naphthol 2,4-Dinitrophenol L-Alanine 5-Methylcytosine Urea L-Glutamine Methyl linolenate m-Chlorohippuric acid Eicosapentaenoic Acid ethyl ester L-Tryptophan Isomaltose Glycerol 5-L-Glutamyl-L-alanine PC(20:5(5Z,8Z,11Z,14Z,17Z)/20:5(5Z,8Z,11Z,14Z,17Z)) Galactinol Pantothenate |
|  | 1 | Stachyose |
| Event24 vs mock | 7 | Phosphoenolpyruvate trans-cinnamate Echinacoside D-Mannitol 1-phosphate Sedoheptulose Vitexin UDP-N-acetylglucosamine |
| Event35 vs mock | 8 | Pro-Asp Leu-Leu Hesperetin Scytalone (R)-3-Hydroxybutyric acid Citramalic acid Deoxyadenosine Adenosine 3',5'-cyclic phosphate (cAMP) |

**Table S5.** Overlapping genes between GM-ZD958 and its parent line as well as non-GM Z58 in growth chamber.

| **Names** | **Total** | **Elements** |
| --- | --- | --- |
| Beijing Zhengzhou Harbin | 731 | Zm00001d040304 Zm00001d002690 Zm00001d049855 Zm00001d012597 Zm00001d020344 Zm00001d038181 Zm00001d046552 Zm00001d009502 Zm00001d045305 Zm00001d012313 Zm00001d011285 Zm00001d026628 Zm00001d016572 Zm00001d044548 Zm00001d044048 Zm00001d004119 Zm00001d032807 Zm00001d051429 Zm00001d045660 Zm00001d052700 Zm00001d043724 Zm00001d031942 Zm00001d034452 Zm00001d023990 Zm00001d046916 Zm00001d034740 Zm00001d016910 Zm00001d039499 Zm00001d023738 Zm00001d003373 Zm00001d011510 Zm00001d010416 Zm00001d001858 Zm00001d018820 Zm00001d023873 Zm00001d028514 Zm00001d035649 Zm00001d028793 Zm00001d051851 Zm00001d031530 Zm00001d019251 Zm00001d026543 Zm00001d029142 Zm00001d039079 Zm00001d002402 Zm00001d005276 Zm00001d019980 Zm00001d045065 Zm00001d034663 Zm00001d039879 Zm00001d045085 Zm00001d053561 Zm00001d002277 Zm00001d022143 Zm00001d032188 Zm00001d015788 Zm00001d047581 Zm00001d008323 Zm00001d042022 Zm00001d021486 Zm00001d041277 Zm00001d022602 Zm00001d019084 Zm00001d052361 Zm00001d050139 Zm00001d044297 Zm00001d005094 Zm00001d032509 Zm00001d037182 Zm00001d006752 Zm00001d016860 Zm00001d008264 Zm00001d027456 Zm00001d023830 Zm00001d020051 Zm00001d049179 Zm00001d031398 Zm00001d031655 Zm00001d027728 Zm00001d044514 Zm00001d013033 Zm00001d036535 Zm00001d004956 Zm00001d012966 Zm00001d053713 Zm00001d028974 Zm00001d045646 Zm00001d019045 Zm00001d017091 Zm00001d030062 Zm00001d044318 Zm00001d019692 Zm00001d007383 Zm00001d018517 Zm00001d032428 Zm00001d034074 Zm00001d028965 Zm00001d045839 Zm00001d014719 Zm00001d016371 Zm00001d016006 Zm00001d032442 Zm00001d045050 Zm00001d031377 Zm00001d048410 Zm00001d017151 Zm00001d003086 Zm00001d050191 Zm00001d001917 Zm00001d018907 Zm00001d001935 Zm00001d021506 Zm00001d030797 Zm00001d031705 Zm00001d002857 Zm00001d050409 Zm00001d017646 Zm00001d047920 Zm00001d031104 Zm00001d038088 Zm00001d042471 Zm00001d006884 Zm00001d026267 Zm00001d005027 Zm00001d031013 Zm00001d039400 Zm00001d051129 Zm00001d052137 Zm00001d011504 Zm00001d040515 Zm00001d004930 Zm00001d037962 Zm00001d024510 Zm00001d008679 Zm00001d052855 Zm00001d041763 Zm00001d005030 Zm00001d030256 Zm00001d051561 Zm00001d040731 Zm00001d039653 Zm00001d011363 Zm00001d038646 Zm00001d047797 Zm00001d022159 Zm00001d012812 Zm00001d006578 Zm00001d006251 Zm00001d003059 Zm00001d038306 Zm00001d045911 Zm00001d022229 Zm00001d011519 Zm00001d025203 Zm00001d048829 Zm00001d040519 Zm00001d018061 Zm00001d011650 Zm00001d004723 Zm00001d044961 Zm00001d036233 Zm00001d037141 Zm00001d024333 Zm00001d002616 Zm00001d015127 Zm00001d027367 Zm00001d026396 Zm00001d012708 Zm00001d036406 Zm00001d035772 Zm00001d048985 Zm00001d022316 Zm00001d010824 Zm00001d026608 Zm00001d049800 Zm00001d042233 Zm00001d035409 Zm00001d034779 Zm00001d021936 Zm00001d042371 Zm00001d007436 Zm00001d048201 Zm00001d043137 Zm00001d015376 Zm00001d031403 Zm00001d014994 Zm00001d020438 Zm00001d025118 Zm00001d001984 Zm00001d025412 Zm00001d012196 Zm00001d046263 Zm00001d004380 Zm00001d009517 Zm00001d016653 Zm00001d033866 Zm00001d037495 Zm00001d007031 Zm00001d042826 Zm00001d004633 Zm00001d041776 Zm00001d018230 Zm00001d004510 Zm00001d005410 Zm00001d049621 Zm00001d048592 Zm00001d018501 Zm00001d013139 Zm00001d019473 Zm00001d018418 Zm00001d006918 Zm00001d021123 Zm00001d032402 Zm00001d023603 Zm00001d018305 Zm00001d048602 Zm00001d049867 Zm00001d013716 Zm00001d026211 Zm00001d003015 Zm00001d018957 Zm00001d034538 Zm00001d010282 Zm00001d037425 Zm00001d047034 Zm00001d006085 Zm00001d012238 Zm00001d015569 Zm00001d012665 Zm00001d031545 Zm00001d015520 Zm00001d027542 Zm00001d002263 Zm00001d020610 Zm00001d018867 Zm00001d028827 Zm00001d006929 Zm00001d036480 Zm00001d028207 Zm00001d048215 Zm00001d038944 Zm00001d050893 Zm00001d037108 Zm00001d006453 Zm00001d017872 Zm00001d005343 Zm00001d009967 Zm00001d002392 Zm00001d044666 Zm00001d032384 Zm00001d048154 Zm00001d049598 Zm00001d037604 Zm00001d017418 Zm00001d014026 Zm00001d018535 Zm00001d037096 Zm00001d016559 Zm00001d042504 Zm00001d007352 Zm00001d035570 Zm00001d021755 Zm00001d054039 Zm00001d032810 Zm00001d036513 Zm00001d047970 Zm00001d004595 Zm00001d033537 Zm00001d008610 Zm00001d046549 Zm00001d001800 Zm00001d003306 Zm00001d004753 Zm00001d037398 Zm00001d008477 Zm00001d034039 Zm00001d021931 Zm00001d022420 Zm00001d035308 Zm00001d028529 Zm00001d014753 Zm00001d018870 Zm00001d011580 Zm00001d014732 Zm00001d049759 Zm00001d006639 Zm00001d041392 Zm00001d015084 Zm00001d007496 Zm00001d049610 Zm00001d018011 Zm00001d019439 Zm00001d019328 Zm00001d002610 Zm00001d011192 Zm00001d036386 Zm00001d006267 Zm00001d048171 Zm00001d017751 Zm00001d003116 Zm00001d013809 Zm00001d046676 Zm00001d037899 Zm00001d049054 Zm00001d032359 Zm00001d043829 Zm00001d016908 Zm00001d044217 Zm00001d002035 Zm00001d041627 Zm00001d043992 Zm00001d026070 Zm00001d036001 Zm00001d029663 Zm00001d017793 Zm00001d041415 Zm00001d050130 Zm00001d033283 Zm00001d019493 Zm00001d042506 Zm00001d022433 Zm00001d020957 Zm00001d035595 Zm00001d011876 Zm00001d033510 Zm00001d043771 Zm00001d039316 Zm00001d035915 Zm00001d044412 Zm00001d025419 Zm00001d044504 Zm00001d046300 Zm00001d046449 Zm00001d034208 Zm00001d050716 Zm00001d035662 Zm00001d037971 Zm00001d036978 Zm00001d025008 Zm00001d015984 Zm00001d045169 Zm00001d039004 Zm00001d018884 Zm00001d011073 Zm00001d043194 Zm00001d038376 Zm00001d038039 Zm00001d016999 Zm00001d044042 Zm00001d018573 Zm00001d046396 Zm00001d040667 Zm00001d004526 Zm00001d017642 Zm00001d050615 Zm00001d036510 Zm00001d008281 Zm00001d029652 Zm00001d012827 Zm00001d002391 Zm00001d035875 Zm00001d025951 Zm00001d035710 Zm00001d047607 Zm00001d035336 Zm00001d018693 Zm00001d026559 Zm00001d047574 Zm00001d007328 Zm00001d052938 Zm00001d041973 Zm00001d035934 Zm00001d012852 Zm00001d049975 Zm00001d034833 Zm00001d012223 Zm00001d011100 Zm00001d053762 Zm00001d018130 Zm00001d045887 Zm00001d032781 Zm00001d050851 Zm00001d022371 Zm00001d052889 Zm00001d045218 Zm00001d035208 Zm00001d024908 Zm00001d051951 Zm00001d038688 Zm00001d039881 Zm00001d025107 Zm00001d049691 Zm00001d054089 Zm00001d047461 Zm00001d022618 Zm00001d020178 Zm00001d020693 Zm00001d031928 Zm00001d048136 Zm00001d025445 Zm00001d036108 Zm00001d038506 Zm00001d017822 Zm00001d040462 Zm00001d008485 Zm00001d012211 Zm00001d049490 Zm00001d010852 Zm00001d015599 Zm00001d039284 Zm00001d008215 Zm00001d032532 Zm00001d019334 Zm00001d051438 Zm00001d016570 Zm00001d051441 Zm00001d014614 Zm00001d011943 Zm00001d044068 Zm00001d005231 Zm00001d049189 Zm00001d043428 Zm00001d031799 Zm00001d020506 Zm00001d043439 Zm00001d014954 Zm00001d012735 Zm00001d038527 Zm00001d012177 Zm00001d009587 Zm00001d044480 Zm00001d044902 Zm00001d033529 Zm00001d043370 Zm00001d002258 Zm00001d012451 Zm00001d022112 Zm00001d034005 Zm00001d032284 Zm00001d036319 Zm00001d044788 Zm00001d003261 Zm00001d032209 Zm00001d009029 Zm00001d026477 Zm00001d037056 Zm00001d016552 Zm00001d031769 Zm00001d031570 Zm00001d053327 Zm00001d019172 Zm00001d012757 Zm00001d021147 Zm00001d052525 Zm00001d044575 Zm00001d013361 Zm00001d002775 Zm00001d028593 Zm00001d007851 Zm00001d033148 Zm00001d029898 Zm00001d025801 Zm00001d019220 Zm00001d012421 Zm00001d036285 Zm00001d020003 Zm00001d021332 Zm00001d026695 Zm00001d036904 Zm00001d002673 Zm00001d042968 Zm00001d050921 Zm00001d009010 Zm00001d021306 Zm00001d052420 Zm00001d010443 Zm00001d050440 Zm00001d027624 Zm00001d019985 Zm00001d034447 Zm00001d052766 Zm00001d025185 Zm00001d014742 Zm00001d043727 Zm00001d016722 Zm00001d043768 Zm00001d022060 Zm00001d043229 Zm00001d003781 Zm00001d010808 Zm00001d052266 Zm00001d013742 Zm00001d043922 Zm00001d007058 Zm00001d002168 Zm00001d002708 Zm00001d027454 Zm00001d046323 Zm00001d042044 Zm00001d012593 Zm00001d051484 Zm00001d024588 Zm00001d048253 Zm00001d012900 Zm00001d048613 Zm00001d044683 Zm00001d007442 Zm00001d037992 Zm00001d038368 Zm00001d043298 Zm00001d023429 Zm00001d031323 Zm00001d011174 Zm00001d054034 Zm00001d048028 Zm00001d018368 Zm00001d023237 Zm00001d032528 Zm00001d033211 Zm00001d039712 Zm00001d013077 Zm00001d018187 Zm00001d041601 Zm00001d032100 Zm00001d051214 Zm00001d043984 Zm00001d006088 Zm00001d008388 Zm00001d023650 Zm00001d041793 Zm00001d037770 Zm00001d036568 Zm00001d014585 Zm00001d034421 Zm00001d020658 Zm00001d006763 Zm00001d048531 Zm00001d053957 Zm00001d033110 Zm00001d040158 Zm00001d048957 Zm00001d035087 Zm00001d012017 Zm00001d030080 Zm00001d024141 Zm00001d043043 Zm00001d020234 Zm00001d006235 Zm00001d034771 Zm00001d012682 Zm00001d009177 Zm00001d011963 Zm00001d017676 Zm00001d024675 Zm00001d032300 Zm00001d008314 Zm00001d018961 Zm00001d031627 Zm00001d041871 Zm00001d042092 Zm00001d029855 Zm00001d027445 Zm00001d052263 Zm00001d017907 Zm00001d004689 Zm00001d031090 Zm00001d046996 Zm00001d013252 Zm00001d042180 Zm00001d043149 Zm00001d018652 Zm00001d016518 Zm00001d028464 Zm00001d022324 Zm00001d004821 Zm00001d025517 Zm00001d041216 Zm00001d025747 Zm00001d029099 Zm00001d048794 Zm00001d011596 Zm00001d015400 Zm00001d053375 Zm00001d044396 Zm00001d026599 Zm00001d007815 Zm00001d030888 Zm00001d004676 Zm00001d003468 Zm00001d014030 Zm00001d046207 Zm00001d016225 Zm00001d018479 Zm00001d001789 Zm00001d030989 Zm00001d018796 Zm00001d014795 Zm00001d053765 Zm00001d010287 Zm00001d008238 Zm00001d030846 Zm00001d036481 Zm00001d035715 Zm00001d023576 Zm00001d027385 Zm00001d043295 Zm00001d014292 Zm00001d027361 Zm00001d022209 Zm00001d026370 Zm00001d017053 Zm00001d042796 Zm00001d042761 Zm00001d037961 Zm00001d022077 Zm00001d005485 Zm00001d004358 Zm00001d025271 Zm00001d001814 Zm00001d034711 Zm00001d041036 Zm00001d045025 Zm00001d027544 Zm00001d031179 Zm00001d011880 Zm00001d013667 Zm00001d002426 Zm00001d036448 Zm00001d033554 Zm00001d038558 Zm00001d051842 Zm00001d053175 Zm00001d011891 Zm00001d029973 Zm00001d034679 Zm00001d032508 Zm00001d035512 Zm00001d018604 Zm00001d041824 Zm00001d011696 Zm00001d012806 Zm00001d026200 Zm00001d050225 Zm00001d052136 Zm00001d047045 Zm00001d031063 Zm00001d038991 Zm00001d049709 Zm00001d053513 Zm00001d038460 Zm00001d041049 Zm00001d025178 Zm00001d021598 Zm00001d051016 Zm00001d035812 Zm00001d020219 Zm00001d026363 Zm00001d033422 Zm00001d024602 Zm00001d015704 Zm00001d037473 Zm00001d045844 Zm00001d014337 Zm00001d035561 Zm00001d032187 Zm00001d012197 Zm00001d037693 Zm00001d014055 Zm00001d052471 Zm00001d018349 Zm00001d006853 Zm00001d045756 Zm00001d034871 Zm00001d017187 Zm00001d020643 Zm00001d043609 Zm00001d049507 Zm00001d003925 Zm00001d029515 Zm00001d011188 Zm00001d007517 Zm00001d002873 Zm00001d039043 Zm00001d011425 Zm00001d048687 Zm00001d042746 Zm00001d010862 Zm00001d049496 Zm00001d053572 Zm00001d045206 Zm00001d047915 Zm00001d019111 Zm00001d052339 Zm00001d033128 Zm00001d049330 Zm00001d024540 Zm00001d017778 Zm00001d042569 Zm00001d043853 Zm00001d036680 Zm00001d040656 Zm00001d034000 Zm00001d014848 Zm00001d011266 Zm00001d039683 Zm00001d017905 Zm00001d034460 Zm00001d048149 Zm00001d050484 Zm00001d044528 Zm00001d020589 Zm00001d040117 Zm00001d006613 Zm00001d044399 Zm00001d048040 Zm00001d040340 Zm00001d036543 Zm00001d011125 Zm00001d013156 Zm00001d052655 Zm00001d038222 Zm00001d025593 Zm00001d029853 Zm00001d045555 Zm00001d041582 Zm00001d035604 Zm00001d017584 Zm00001d013892 Zm00001d041267 Zm00001d033751 Zm00001d046682 Zm00001d053786 Zm00001d009089 Zm00001d011782 Zm00001d035032 Zm00001d053722 Zm00001d043387 Zm00001d030152 Zm00001d040534 Zm00001d049100 Zm00001d039911 Zm00001d052389 Zm00001d036092 Zm00001d053432 Zm00001d009127 Zm00001d031807 Zm00001d031430 Zm00001d053913 Zm00001d008904 Zm00001d006046 Zm00001d051823 Zm00001d009948 Zm00001d011668 Zm00001d051554 Zm00001d020556 Zm00001d048479 Zm00001d048256 Zm00001d020399 Zm00001d050403 Zm00001d002455 Zm00001d016318 Zm00001d013435 Zm00001d020867 Zm00001d033994 Zm00001d006065 Zm00001d038361 Zm00001d006860 Zm00001d048802 Zm00001d052215 Zm00001d043253 Zm00001d023808 Zm00001d019542 Zm00001d026600 Zm00001d028098 Zm00001d024105 Zm00001d042777 Zm00001d039856 Zm00001d031738 Zm00001d031211 Zm00001d012518 Zm00001d019547 Zm00001d016687 Zm00001d021336 Zm00001d033337 Zm00001d053885 Zm00001d048100 Zm00001d034529 Zm00001d039918 Zm00001d021574 Zm00001d004021 Zm00001d025326 Zm00001d039182 Zm00001d032229 Zm00001d016188 Zm00001d017119 Zm00001d010970 Zm00001d012465 Zm00001d041965 Zm00001d012854 Zm00001d013779 Zm00001d006494 Zm00001d031303 Zm00001d017714 Zm00001d021721 Zm00001d027479 Zm00001d033167 Zm00001d035876 Zm00001d051388 Zm00001d013469 Zm00001d041148 Zm00001d017456 Zm00001d042737 Zm00001d025431 Zm00001d008839 Zm00001d050365 Zm00001d006470 Zm00001d020205 Zm00001d042939 Zm00001d026542 Zm00001d018342 Zm00001d005303 Zm00001d017334 Zm00001d002694 Zm00001d050248 Zm00001d050380 Zm00001d037449 Zm00001d018149 Zm00001d038224 Zm00001d028575 Zm00001d018099 Zm00001d022631 Zm00001d005599 Zm00001d051161 Zm00001d052010 Zm00001d013831 Zm00001d045188 Zm00001d038242 Zm00001d014726 Zm00001d010971 Zm00001d053919 Zm00001d037596 Zm00001d039453 Zm00001d012544 Zm00001d025023 Zm00001d010607 Zm00001d009653 Zm00001d029675 Zm00001d036410 Zm00001d002284 Zm00001d037727 Zm00001d018065 Zm00001d048539 Zm00001d034427 Zm00001d036417 Zm00001d049597 Zm00001d012393 Zm00001d033132 Zm00001d028463 Zm00001d034865 Zm00001d037687 Zm00001d019706 Zm00001d041927 Zm00001d043380 Zm00001d035802 Zm00001d047829 Zm00001d039580 Zm00001d028643 Zm00001d026212 Zm00001d028625 Zm00001d013100 Zm00001d049365 Zm00001d022277 Zm00001d017708 Zm00001d034320 Zm00001d021595 Zm00001d020170 Zm00001d053223 Zm00001d011648 Zm00001d009606 Zm00001d031809 Zm00001d006793 Zm00001d034608 Zm00001d043056 Zm00001d027276 Zm00001d047965 Zm00001d053687 Zm00001d022366 Zm00001d027675 Zm00001d011831 Zm00001d037288 Zm00001d051178 Zm00001d042302 Zm00001d017110 Zm00001d053273 Zm00001d036894 Zm00001d032609 Zm00001d004875 Zm00001d037551 Zm00001d018366 Zm00001d014436 Zm00001d029743 Zm00001d018081 Zm00001d020444 Zm00001d049020 Zm00001d045049 Zm00001d048545 Zm00001d038147 Zm00001d003683 Zm00001d029084 Zm00001d006463 Zm00001d013415 Zm00001d003369 Zm00001d017957 Zm00001d009401 Zm00001d010538 Zm00001d010026 Zm00001d046927 Zm00001d010816 Zm00001d015505 Zm00001d035659 Zm00001d011471 Zm00001d040883 Zm00001d014953 Zm00001d047423 Zm00001d019721 Zm00001d032459 Zm00001d037784 Zm00001d009552 Zm00001d052951 Zm00001d012339 Zm00001d044402 Zm00001d043083 Zm00001d008620 Zm00001d033652 Zm00001d043991 Zm00001d034244 Zm00001d029595 Zm00001d044043 Zm00001d005482 Zm00001d049370 Zm00001d024635 Zm00001d049815 Zm00001d040191 Zm00001d043814 Zm00001d043528 Zm00001d036454 Zm00001d007290 Zm00001d028995 Zm00001d028867 Zm00001d047345 Zm00001d023839 Zm00001d031634 Zm00001d012953 Zm00001d021891 Zm00001d029047 Zm00001d046508 Zm00001d028558 Zm00001d039624 Zm00001d024534 Zm00001d027375 Zm00001d040545 Zm00001d013294 Zm00001d043113 Zm00001d025079 Zm00001d051307 Zm00001d005638 Zm00001d045787 Zm00001d035401 Zm00001d002475 Zm00001d041352 Zm00001d007441 Zm00001d043625 Zm00001d016254 Zm00001d038546 Zm00001d052675 Zm00001d014449 Zm00001d021203 Zm00001d048954 Zm00001d037590 Zm00001d037274 Zm00001d036655 Zm00001d018758 Zm00001d009610 Zm00001d027756 Zm00001d033986 Zm00001d043726 Zm00001d028823 Zm00001d050324 Zm00001d016446 Zm00001d020366 Zm00001d031671 Zm00001d007900 Zm00001d005765 Zm00001d026214 Zm00001d008457 Zm00001d047796 Zm00001d016551 Zm00001d019191 Zm00001d029249 Zm00001d006479 Zm00001d031028 Zm00001d042512 Zm00001d033044 Zm00001d051234 Zm00001d020134 Zm00001d027326 Zm00001d030892 Zm00001d006221 Zm00001d013105 Zm00001d017659 Zm00001d049579 Zm00001d013614 Zm00001d049305 Zm00001d004819 Zm00001d036547 Zm00001d051241 Zm00001d043249 Zm00001d027314 Zm00001d036540 Zm00001d032736 Zm00001d006823 Zm00001d028451 Zm00001d037246 Zm00001d031688 Zm00001d035600 Zm00001d013411 Zm00001d026254 Zm00001d022603 Zm00001d006461 Zm00001d009902 Zm00001d035770 Zm00001d041698 Zm00001d007009 Zm00001d044288 Zm00001d002917 Zm00001d010519 Zm00001d034919 Zm00001d033830 Zm00001d025570 Zm00001d029502 Zm00001d013568 Zm00001d021946 Zm00001d047856 Zm00001d020783 Zm00001d026476 Zm00001d011197 Zm00001d044172 Zm00001d048766 Zm00001d025777 Zm00001d038136 Zm00001d006211 Zm00001d034399 Zm00001d037485 Zm00001d029725 Zm00001d003386 Zm00001d040084 Zm00001d040107 Zm00001d005137 Zm00001d020902 Zm00001d013606 Zm00001d010733 Zm00001d006939 Zm00001d034205 Zm00001d034977 Zm00001d029717 Zm00001d051779 Zm00001d028704 Zm00001d048006 Zm00001d032230 Zm00001d046242 Zm00001d003249 Zm00001d047534 Zm00001d049242 Zm00001d008287 Zm00001d033684 Zm00001d040743 Zm00001d020521 Zm00001d026290 Zm00001d044243 Zm00001d012930 Zm00001d033873 Zm00001d048372 Zm00001d046592 Zm00001d053899 Zm00001d003800 Zm00001d044307 Zm00001d012420 Zm00001d042059 Zm00001d011848 Zm00001d035168 Zm00001d029454 |
| Beijing Harbin | 603 | Zm00001d005149 Zm00001d050872 Zm00001d039012 Zm00001d012743 Zm00001d029478 Zm00001d033287 Zm00001d018343 Zm00001d032197 Zm00001d043734 Zm00001d039492 Zm00001d021906 Zm00001d019241 Zm00001d007757 Zm00001d052895 Zm00001d011636 Zm00001d002288 Zm00001d014456 Zm00001d012438 Zm00001d036784 Zm00001d004916 Zm00001d044618 Zm00001d012340 Zm00001d023803 Zm00001d030370 Zm00001d039483 Zm00001d042463 Zm00001d048763 Zm00001d008805 Zm00001d013126 Zm00001d037553 Zm00001d039914 Zm00001d042257 Zm00001d037228 Zm00001d036245 Zm00001d036615 Zm00001d045123 Zm00001d034739 Zm00001d013210 Zm00001d052656 Zm00001d049343 Zm00001d015060 Zm00001d033168 Zm00001d047707 Zm00001d031800 Zm00001d053409 Zm00001d008211 Zm00001d017069 Zm00001d033396 Zm00001d013612 Zm00001d024825 Zm00001d040644 Zm00001d013295 Zm00001d006446 Zm00001d017837 Zm00001d049657 Zm00001d010662 Zm00001d021719 Zm00001d033139 Zm00001d030074 Zm00001d042093 Zm00001d049581 Zm00001d034772 Zm00001d033787 Zm00001d008764 Zm00001d021509 Zm00001d016649 Zm00001d030372 Zm00001d031875 Zm00001d008595 Zm00001d049911 Zm00001d006856 Zm00001d022469 Zm00001d035002 Zm00001d009676 Zm00001d008727 Zm00001d014060 Zm00001d031484 Zm00001d009339 Zm00001d038228 Zm00001d015327 Zm00001d004587 Zm00001d012600 Zm00001d038490 Zm00001d029703 Zm00001d052684 Zm00001d034041 Zm00001d052264 Zm00001d043465 Zm00001d034082 Zm00001d003510 Zm00001d050368 Zm00001d022240 Zm00001d002283 Zm00001d053926 Zm00001d047616 Zm00001d047293 Zm00001d042910 Zm00001d029772 Zm00001d011446 Zm00001d026598 Zm00001d015164 Zm00001d005017 Zm00001d043414 Zm00001d045740 Zm00001d046299 Zm00001d016796 Zm00001d007929 Zm00001d037969 Zm00001d003525 Zm00001d017146 Zm00001d031217 Zm00001d043555 Zm00001d012287 Zm00001d051924 Zm00001d022391 Zm00001d034350 Zm00001d036827 Zm00001d044833 Zm00001d005881 Zm00001d031862 Zm00001d021337 Zm00001d005092 Zm00001d018090 Zm00001d001841 Zm00001d016896 Zm00001d015101 Zm00001d043965 Zm00001d033289 Zm00001d004817 Zm00001d025453 Zm00001d039887 Zm00001d037167 Zm00001d003114 Zm00001d004137 Zm00001d033225 Zm00001d051093 Zm00001d017178 Zm00001d038409 Zm00001d024612 Zm00001d042583 Zm00001d015200 Zm00001d002252 Zm00001d018597 Zm00001d051568 Zm00001d016744 Zm00001d024058 Zm00001d022275 Zm00001d050982 Zm00001d042035 Zm00001d038676 Zm00001d027729 Zm00001d047696 Zm00001d008499 Zm00001d044977 Zm00001d032921 Zm00001d017536 Zm00001d009814 Zm00001d045330 Zm00001d034796 Zm00001d048578 Zm00001d012780 Zm00001d005059 Zm00001d034555 Zm00001d026000 Zm00001d008228 Zm00001d031726 Zm00001d021873 Zm00001d038155 Zm00001d015031 Zm00001d034139 Zm00001d037689 Zm00001d007103 Zm00001d021058 Zm00001d017019 Zm00001d031278 Zm00001d041681 Zm00001d018364 Zm00001d028732 Zm00001d026439 Zm00001d041618 Zm00001d049525 Zm00001d022310 Zm00001d020383 Zm00001d034617 Zm00001d017669 Zm00001d018911 Zm00001d020686 Zm00001d042027 Zm00001d046317 Zm00001d003148 Zm00001d038392 Zm00001d022354 Zm00001d015737 Zm00001d046792 Zm00001d026222 Zm00001d031682 Zm00001d042166 Zm00001d037611 Zm00001d013031 Zm00001d017137 Zm00001d042008 Zm00001d036162 Zm00001d025644 Zm00001d015320 Zm00001d045563 Zm00001d012611 Zm00001d013289 Zm00001d018388 Zm00001d013589 Zm00001d049173 Zm00001d020445 Zm00001d028679 Zm00001d009960 Zm00001d037476 Zm00001d037774 Zm00001d008199 Zm00001d013943 Zm00001d002012 Zm00001d018530 Zm00001d038430 Zm00001d032375 Zm00001d011474 Zm00001d028394 Zm00001d001797 Zm00001d051194 Zm00001d005579 Zm00001d047738 Zm00001d044267 Zm00001d032900 Zm00001d045145 Zm00001d042843 Zm00001d018097 Zm00001d013817 Zm00001d029060 Zm00001d047476 Zm00001d049894 Zm00001d033079 Zm00001d014191 Zm00001d018614 Zm00001d004196 Zm00001d015927 Zm00001d044898 Zm00001d015204 Zm00001d012488 Zm00001d007886 Zm00001d012205 Zm00001d029126 Zm00001d053925 Zm00001d051342 Zm00001d040109 Zm00001d041778 Zm00001d004340 Zm00001d034759 Zm00001d052240 Zm00001d035343 Zm00001d036135 Zm00001d038842 Zm00001d043997 Zm00001d038300 Zm00001d008177 Zm00001d034558 Zm00001d031992 Zm00001d027463 Zm00001d048621 Zm00001d007633 Zm00001d014863 Zm00001d029378 Zm00001d008466 Zm00001d038161 Zm00001d037417 Zm00001d036771 Zm00001d019181 Zm00001d047789 Zm00001d031364 Zm00001d038555 Zm00001d032055 Zm00001d034283 Zm00001d007034 Zm00001d017409 Zm00001d021135 Zm00001d048397 Zm00001d012353 Zm00001d020524 Zm00001d029333 Zm00001d036404 Zm00001d053548 Zm00001d036788 Zm00001d047958 Zm00001d042104 Zm00001d018331 Zm00001d036825 Zm00001d045327 Zm00001d008435 Zm00001d039848 Zm00001d046591 Zm00001d021334 Zm00001d041690 Zm00001d049080 Zm00001d028984 Zm00001d045339 Zm00001d032918 Zm00001d022244 Zm00001d034346 Zm00001d039658 Zm00001d044045 Zm00001d007491 Zm00001d034852 Zm00001d044857 Zm00001d006714 Zm00001d014789 Zm00001d047935 Zm00001d015025 Zm00001d021435 Zm00001d037811 Zm00001d007018 Zm00001d003670 Zm00001d031666 Zm00001d013008 Zm00001d040623 Zm00001d031248 Zm00001d016484 Zm00001d001994 Zm00001d014084 Zm00001d010410 Zm00001d028822 Zm00001d012646 Zm00001d022044 Zm00001d053394 Zm00001d017077 Zm00001d002411 Zm00001d036660 Zm00001d045517 Zm00001d012254 Zm00001d025406 Zm00001d006230 Zm00001d003589 Zm00001d002940 Zm00001d025998 Zm00001d031220 Zm00001d050754 Zm00001d007908 Zm00001d042780 Zm00001d041456 Zm00001d032420 Zm00001d019040 Zm00001d024467 Zm00001d026442 Zm00001d016122 Zm00001d013256 Zm00001d034112 Zm00001d027601 Zm00001d013166 Zm00001d048453 Zm00001d041536 Zm00001d045575 Zm00001d012922 Zm00001d039643 Zm00001d046679 Zm00001d030267 Zm00001d042290 Zm00001d008585 Zm00001d026194 Zm00001d042692 Zm00001d012460 Zm00001d037550 Zm00001d008396 Zm00001d009276 Zm00001d008181 Zm00001d020290 Zm00001d008852 Zm00001d033874 Zm00001d035162 Zm00001d025474 Zm00001d036440 Zm00001d050033 Zm00001d034732 Zm00001d015504 Zm00001d054080 Zm00001d002576 Zm00001d009774 Zm00001d025861 Zm00001d037653 Zm00001d026286 Zm00001d023915 Zm00001d040108 Zm00001d024843 Zm00001d005081 Zm00001d040725 Zm00001d007484 Zm00001d017958 Zm00001d047855 Zm00001d027341 Zm00001d052622 Zm00001d014652 Zm00001d050168 Zm00001d006130 Zm00001d032925 Zm00001d002003 Zm00001d041982 Zm00001d048060 Zm00001d014013 Zm00001d012911 Zm00001d030392 Zm00001d031216 Zm00001d047942 Zm00001d029718 Zm00001d003088 Zm00001d009148 Zm00001d004918 Zm00001d014063 Zm00001d025362 Zm00001d052847 Zm00001d018341 Zm00001d024274 Zm00001d048493 Zm00001d016892 Zm00001d049501 Zm00001d022166 Zm00001d014606 Zm00001d018150 Zm00001d042767 Zm00001d025252 Zm00001d014494 Zm00001d030612 Zm00001d026291 Zm00001d024854 Zm00001d003848 Zm00001d008842 Zm00001d006683 Zm00001d048032 Zm00001d027874 Zm00001d042536 Zm00001d015007 Zm00001d037354 Zm00001d017099 Zm00001d042594 Zm00001d048775 Zm00001d014518 Zm00001d053928 Zm00001d002053 Zm00001d040331 Zm00001d012213 Zm00001d053569 Zm00001d004338 Zm00001d046980 Zm00001d037275 Zm00001d047640 Zm00001d020401 Zm00001d035206 Zm00001d018218 Zm00001d020355 Zm00001d004560 Zm00001d029620 Zm00001d018941 Zm00001d049380 Zm00001d008628 Zm00001d047174 Zm00001d020023 Zm00001d039422 Zm00001d032269 Zm00001d040777 Zm00001d025539 Zm00001d051912 Zm00001d040554 Zm00001d007839 Zm00001d002456 Zm00001d047742 Zm00001d012670 Zm00001d013346 Zm00001d022006 Zm00001d032830 Zm00001d044434 Zm00001d006625 Zm00001d032671 Zm00001d011931 Zm00001d008957 Zm00001d035608 Zm00001d028522 Zm00001d026560 Zm00001d011351 Zm00001d032694 Zm00001d051662 Zm00001d018965 Zm00001d040920 Zm00001d034880 Zm00001d042005 Zm00001d008516 Zm00001d028696 Zm00001d032999 Zm00001d034533 Zm00001d043858 Zm00001d037779 Zm00001d049766 Zm00001d052060 Zm00001d023669 Zm00001d039104 Zm00001d032328 Zm00001d029745 Zm00001d035312 Zm00001d021259 Zm00001d005323 Zm00001d029451 Zm00001d020599 Zm00001d028533 Zm00001d038491 Zm00001d028980 Zm00001d022445 Zm00001d047452 Zm00001d050196 Zm00001d036839 Zm00001d031349 Zm00001d034319 Zm00001d018337 Zm00001d048026 Zm00001d040570 Zm00001d010201 Zm00001d028601 Zm00001d006286 Zm00001d004586 Zm00001d034088 Zm00001d029274 Zm00001d036663 Zm00001d007903 Zm00001d047766 Zm00001d017282 Zm00001d018297 Zm00001d041649 Zm00001d015777 Zm00001d004768 Zm00001d004837 Zm00001d013439 Zm00001d042503 Zm00001d007349 Zm00001d048495 Zm00001d003850 Zm00001d047930 Zm00001d029693 Zm00001d008187 Zm00001d046739 Zm00001d053727 Zm00001d035766 Zm00001d030849 Zm00001d011211 Zm00001d034095 Zm00001d024598 Zm00001d005458 Zm00001d040216 Zm00001d018727 Zm00001d027763 Zm00001d024471 Zm00001d022619 Zm00001d027296 Zm00001d030767 Zm00001d052297 Zm00001d004209 Zm00001d029707 Zm00001d031850 Zm00001d039468 Zm00001d015088 Zm00001d013063 Zm00001d030159 Zm00001d029903 Zm00001d002859 Zm00001d032040 Zm00001d002595 Zm00001d027488 Zm00001d024242 Zm00001d035140 Zm00001d028676 Zm00001d001850 Zm00001d003228 Zm00001d037745 Zm00001d026391 Zm00001d044728 Zm00001d036545 Zm00001d013288 Zm00001d002346 Zm00001d017895 Zm00001d007401 Zm00001d037383 Zm00001d049162 Zm00001d034604 Zm00001d042464 Zm00001d044808 Zm00001d016841 Zm00001d014603 Zm00001d053965 Zm00001d045208 Zm00001d051499 Zm00001d017279 Zm00001d012983 Zm00001d035392 Zm00001d004332 Zm00001d043144 Zm00001d046416 Zm00001d052586 Zm00001d034383 Zm00001d014925 Zm00001d030795 Zm00001d031159 Zm00001d041959 Zm00001d039140 Zm00001d018927 Zm00001d045729 Zm00001d018374 Zm00001d013080 Zm00001d039197 Zm00001d037732 Zm00001d036668 Zm00001d039710 Zm00001d020448 Zm00001d015090 Zm00001d012485 Zm00001d043459 Zm00001d022374 Zm00001d032742 Zm00001d002125 Zm00001d039193 Zm00001d013886 Zm00001d008645 Zm00001d048409 Zm00001d032747 Zm00001d026488 Zm00001d028665 Zm00001d016987 Zm00001d031329 Zm00001d012761 Zm00001d042182 Zm00001d006159 Zm00001d003140 Zm00001d036214 Zm00001d006194 Zm00001d035184 Zm00001d012781 Zm00001d020770 Zm00001d003614 Zm00001d008569 Zm00001d048709 Zm00001d013092 Zm00001d049590 Zm00001d029885 Zm00001d037790 Zm00001d005325 Zm00001d026124 Zm00001d039176 Zm00001d037836 Zm00001d013610 Zm00001d039789 Zm00001d013045 Zm00001d015579 Zm00001d035139 Zm00001d042533 Zm00001d046480 Zm00001d026414 Zm00001d016797 Zm00001d001866 Zm00001d001861 Zm00001d045528 Zm00001d032499 Zm00001d003872 Zm00001d046318 Zm00001d029415 Zm00001d027887 Zm00001d015519 Zm00001d051450 Zm00001d052478 Zm00001d013643 Zm00001d053412 Zm00001d044492 Zm00001d037547 Zm00001d038206 Zm00001d012794 Zm00001d046716 Zm00001d045573 Zm00001d014885 Zm00001d028576 Zm00001d047157 Zm00001d049661 Zm00001d027752 Zm00001d047472 Zm00001d053709 Zm00001d007038 Zm00001d006589 Zm00001d045076 Zm00001d001771 Zm00001d041103 Zm00001d042476 Zm00001d051448 Zm00001d024861 Zm00001d003045 Zm00001d046949 Zm00001d028630 Zm00001d038049 Zm00001d049954 Zm00001d014811 Zm00001d041697 Zm00001d044537 Zm00001d049088 Zm00001d047469 Zm00001d004473 Zm00001d051490 Zm00001d044467 Zm00001d049009 Zm00001d014284 Zm00001d011886 Zm00001d038251 Zm00001d046725 Zm00001d046765 Zm00001d018611 Zm00001d002377 Zm00001d029801 Zm00001d011461 Zm00001d031412 Zm00001d032458 Zm00001d033039 Zm00001d007912 Zm00001d031471 Zm00001d009617 Zm00001d046454 Zm00001d007157 Zm00001d015743 Zm00001d008869 Zm00001d017276 Zm00001d036898 Zm00001d022517 Zm00001d048856 Zm00001d019513 Zm00001d041389 Zm00001d003575 Zm00001d045487 Zm00001d014734 Zm00001d036929 Zm00001d047913 Zm00001d014356 Zm00001d009913 Zm00001d043089 Zm00001d052507 Zm00001d006563 Zm00001d005015 Zm00001d018894 Zm00001d006321 Zm00001d003841 Zm00001d012170 Zm00001d030040 Zm00001d040463 Zm00001d019409 Zm00001d003304 Zm00001d020280 Zm00001d004513 Zm00001d002410 Zm00001d052123 Zm00001d008700 Zm00001d012599 Zm00001d052480 Zm00001d052673 Zm00001d044821 Zm00001d038130 Zm00001d034851 Zm00001d024713 Zm00001d029653 Zm00001d008230 Zm00001d025171 Zm00001d017821 Zm00001d037605 Zm00001d017249 Zm00001d027480 Zm00001d053724 Zm00001d018500 Zm00001d013543 Zm00001d019100 Zm00001d024788 Zm00001d043299 Zm00001d029325 Zm00001d034020 Zm00001d038712 Zm00001d035456 Zm00001d003855 Zm00001d020679 Zm00001d023430 Zm00001d002167 Zm00001d011123 Zm00001d037493 Zm00001d049313 Zm00001d006107 Zm00001d010756 Zm00001d014082 Zm00001d044431 Zm00001d017389 Zm00001d047504 Zm00001d019376 Zm00001d027743 Zm00001d013076 Zm00001d016553 Zm00001d030542 Zm00001d033090 Zm00001d025855 Zm00001d034534 Zm00001d041334 Zm00001d044922 Zm00001d051634 Zm00001d027525 Zm00001d047250 Zm00001d018586 Zm00001d042192 Zm00001d021784 Zm00001d016474 Zm00001d031549 Zm00001d047912 Zm00001d032831 Zm00001d007697 Zm00001d046226 Zm00001d021948 Zm00001d036361 Zm00001d014658 Zm00001d036575 Zm00001d044985 Zm00001d027824 Zm00001d018259 Zm00001d043191 Zm00001d007934 Zm00001d045144 Zm00001d016768 Zm00001d002034 Zm00001d029144 Zm00001d038057 Zm00001d024519 Zm00001d039510 Zm00001d051567 Zm00001d015497 Zm00001d027911 Zm00001d023223 Zm00001d034189 Zm00001d045042 Zm00001d021739 Zm00001d048113 Zm00001d051860 Zm00001d031265 Zm00001d042400 Zm00001d050371 Zm00001d015623 Zm00001d025000 Zm00001d031379 Zm00001d045436 Zm00001d007166 Zm00001d039546 Zm00001d031168 Zm00001d013360 Zm00001d017780 Zm00001d021713 Zm00001d043280 Zm00001d026522 Zm00001d047934 Zm00001d039434 Zm00001d037050 Zm00001d005971 Zm00001d040244 Zm00001d003154 Zm00001d037532 Zm00001d008997 Zm00001d018610 Zm00001d022258 Zm00001d052933 Zm00001d047526 Zm00001d050043 Zm00001d013787 Zm00001d006316 Zm00001d006571 Zm00001d007843 Zm00001d053923 Zm00001d046981 Zm00001d039732 Zm00001d016068 Zm00001d025845 Zm00001d018356 Zm00001d046202 Zm00001d003553 Zm00001d003332 Zm00001d021244 Zm00001d038019 Zm00001d003120 Zm00001d029705 Zm00001d048356 Zm00001d010240 Zm00001d043228 Zm00001d032298 Zm00001d052679 Zm00001d040375 Zm00001d020497 Zm00001d052003 Zm00001d049641 Zm00001d007059 Zm00001d030045 Zm00001d008255 Zm00001d006684 Zm00001d018839 Zm00001d015578 Zm00001d034894 Zm00001d012677 Zm00001d026665 Zm00001d047347 Zm00001d044555 Zm00001d024413 Zm00001d008781 Zm00001d030090 Zm00001d047743 Zm00001d027523 Zm00001d034532 Zm00001d011527 Zm00001d053208 Zm00001d021515 Zm00001d017060 Zm00001d053084 Zm00001d008176 Zm00001d042693 Zm00001d019006 Zm00001d023904 Zm00001d021718 Zm00001d047138 Zm00001d019550 Zm00001d039713 Zm00001d047403 Zm00001d017024 Zm00001d034067 Zm00001d028988 Zm00001d047671 Zm00001d013569 Zm00001d047864 Zm00001d042210 Zm00001d048514 Zm00001d025342 Zm00001d031416 Zm00001d012632 Zm00001d040628 Zm00001d045519 Zm00001d028591 Zm00001d044040 Zm00001d019356 Zm00001d012090 Zm00001d017208 Zm00001d045193 Zm00001d012206 Zm00001d043218 Zm00001d018009 Zm00001d022394 Zm00001d030559 Zm00001d028585 Zm00001d005375 Zm00001d033704 Zm00001d011269 Zm00001d003152 Zm00001d020353 Zm00001d043512 Zm00001d042215 Zm00001d038045 Zm00001d032036 Zm00001d026421 Zm00001d042555 Zm00001d008592 Zm00001d039581 Zm00001d025509 Zm00001d048737 Zm00001d012882 Zm00001d052584 Zm00001d028810 Zm00001d026206 Zm00001d044127 Zm00001d039727 Zm00001d052488 Zm00001d032750 Zm00001d038999 Zm00001d012532 Zm00001d039363 Zm00001d024683 Zm00001d018544 Zm00001d013185 Zm00001d031263 Zm00001d039768 Zm00001d032138 |
| Beijing Zhengzhou | 2805 | Zm00001d002425 Zm00001d025661 Zm00001d039711 Zm00001d021346 Zm00001d013993 Zm00001d040322 Zm00001d042966 Zm00001d020002 Zm00001d007163 Zm00001d037018 Zm00001d011548 Zm00001d022541 Zm00001d018987 Zm00001d009837 Zm00001d003922 Zm00001d043505 Zm00001d035579 Zm00001d017773 Zm00001d049960 Zm00001d045354 Zm00001d049361 Zm00001d045028 Zm00001d021599 Zm00001d004442 Zm00001d030691 Zm00001d008992 Zm00001d033305 Zm00001d015817 Zm00001d002847 Zm00001d026248 Zm00001d013026 Zm00001d048065 Zm00001d035663 Zm00001d009387 Zm00001d045159 Zm00001d029504 Zm00001d031969 Zm00001d019262 Zm00001d052444 Zm00001d026014 Zm00001d021070 Zm00001d013135 Zm00001d031902 Zm00001d036524 Zm00001d009700 Zm00001d018946 Zm00001d042421 Zm00001d012198 Zm00001d018016 Zm00001d041052 Zm00001d029138 Zm00001d051112 Zm00001d041710 Zm00001d015300 Zm00001d028104 Zm00001d028408 Zm00001d037200 Zm00001d019358 Zm00001d034255 Zm00001d037602 Zm00001d018348 Zm00001d034231 Zm00001d024301 Zm00001d032911 Zm00001d014129 Zm00001d011741 Zm00001d044757 Zm00001d035016 Zm00001d030554 Zm00001d030832 Zm00001d014926 Zm00001d036145 Zm00001d053671 Zm00001d034404 Zm00001d009667 Zm00001d049694 Zm00001d002659 Zm00001d045128 Zm00001d020707 Zm00001d051870 Zm00001d012160 Zm00001d017850 Zm00001d029409 Zm00001d044869 Zm00001d033526 Zm00001d028116 Zm00001d027688 Zm00001d016674 Zm00001d009496 Zm00001d048857 Zm00001d021582 Zm00001d031654 Zm00001d030549 Zm00001d004344 Zm00001d044667 Zm00001d002292 Zm00001d054065 Zm00001d041243 Zm00001d046593 Zm00001d049886 Zm00001d012832 Zm00001d001909 Zm00001d023759 Zm00001d038105 Zm00001d003680 Zm00001d041684 Zm00001d002823 Zm00001d009978 Zm00001d033066 Zm00001d031024 Zm00001d042835 Zm00001d033714 Zm00001d043497 Zm00001d045294 Zm00001d008196 Zm00001d032496 Zm00001d047107 Zm00001d008245 Zm00001d002941 Zm00001d005102 Zm00001d031292 Zm00001d052062 Zm00001d031178 Zm00001d048163 Zm00001d046402 Zm00001d047149 Zm00001d046369 Zm00001d048469 Zm00001d018309 Zm00001d044954 Zm00001d033046 Zm00001d014958 Zm00001d021006 Zm00001d036213 Zm00001d005920 Zm00001d010030 Zm00001d049239 Zm00001d040235 Zm00001d019253 Zm00001d008752 Zm00001d031037 Zm00001d047699 Zm00001d053830 Zm00001d012263 Zm00001d032072 Zm00001d004413 Zm00001d027335 Zm00001d042325 Zm00001d024463 Zm00001d052797 Zm00001d034509 Zm00001d039167 Zm00001d048739 Zm00001d007183 Zm00001d028680 Zm00001d007147 Zm00001d042025 Zm00001d047477 Zm00001d036641 Zm00001d032292 Zm00001d025229 Zm00001d034515 Zm00001d016378 Zm00001d013012 Zm00001d014749 Zm00001d012003 Zm00001d016358 Zm00001d046434 Zm00001d025057 Zm00001d003125 Zm00001d028325 Zm00001d047296 Zm00001d031044 Zm00001d039971 Zm00001d003344 Zm00001d009797 Zm00001d018085 Zm00001d041192 Zm00001d013572 Zm00001d044393 Zm00001d049732 Zm00001d043261 Zm00001d037616 Zm00001d016238 Zm00001d013548 Zm00001d034263 Zm00001d044340 Zm00001d048159 Zm00001d053872 Zm00001d019219 Zm00001d021418 Zm00001d010204 Zm00001d027738 Zm00001d007039 Zm00001d035136 Zm00001d037098 Zm00001d021834 Zm00001d008244 Zm00001d011500 Zm00001d042184 Zm00001d020825 Zm00001d037859 Zm00001d040986 Zm00001d024878 Zm00001d045589 Zm00001d038547 Zm00001d039497 Zm00001d012861 Zm00001d044106 Zm00001d011245 Zm00001d045000 Zm00001d033858 Zm00001d017205 Zm00001d045320 Zm00001d011632 Zm00001d009868 Zm00001d026397 Zm00001d048291 Zm00001d050810 Zm00001d011165 Zm00001d032158 Zm00001d010504 Zm00001d047387 Zm00001d022089 Zm00001d013184 Zm00001d019048 Zm00001d045450 Zm00001d044517 Zm00001d034855 Zm00001d007366 Zm00001d031259 Zm00001d012897 Zm00001d051589 Zm00001d018485 Zm00001d005453 Zm00001d012441 Zm00001d012549 Zm00001d040205 Zm00001d049902 Zm00001d044417 Zm00001d034528 Zm00001d011845 Zm00001d002019 Zm00001d016408 Zm00001d018629 Zm00001d037816 Zm00001d002759 Zm00001d007971 Zm00001d039452 Zm00001d007123 Zm00001d033879 Zm00001d005170 Zm00001d016435 Zm00001d034929 Zm00001d013449 Zm00001d038840 Zm00001d021842 Zm00001d016691 Zm00001d030300 Zm00001d009870 Zm00001d034143 Zm00001d045938 Zm00001d018080 Zm00001d016458 Zm00001d012265 Zm00001d027369 Zm00001d022628 Zm00001d005558 Zm00001d019656 Zm00001d028231 Zm00001d013015 Zm00001d005727 Zm00001d034380 Zm00001d009178 Zm00001d001838 Zm00001d016720 Zm00001d009494 Zm00001d047783 Zm00001d012964 Zm00001d011364 Zm00001d043621 Zm00001d048091 Zm00001d019087 Zm00001d051916 Zm00001d014355 Zm00001d040807 Zm00001d017320 Zm00001d045494 Zm00001d052474 Zm00001d003066 Zm00001d048366 Zm00001d049204 Zm00001d010615 Zm00001d041290 Zm00001d007573 Zm00001d027516 Zm00001d006315 Zm00001d048799 Zm00001d004804 Zm00001d006624 Zm00001d034461 Zm00001d012322 Zm00001d052682 Zm00001d045836 Zm00001d018763 Zm00001d033780 Zm00001d032661 Zm00001d025914 Zm00001d034192 Zm00001d013434 Zm00001d015865 Zm00001d013626 Zm00001d028112 Zm00001d002358 Zm00001d003319 Zm00001d051929 Zm00001d043411 Zm00001d003676 Zm00001d018967 Zm00001d041682 Zm00001d024676 Zm00001d047448 Zm00001d017303 Zm00001d032790 Zm00001d021759 Zm00001d030167 Zm00001d014155 Zm00001d047788 Zm00001d046714 Zm00001d013257 Zm00001d015135 Zm00001d035481 Zm00001d039665 Zm00001d021413 Zm00001d023654 Zm00001d005006 Zm00001d008725 Zm00001d043854 Zm00001d019481 Zm00001d031527 Zm00001d044358 Zm00001d034609 Zm00001d004357 Zm00001d016075 Zm00001d037052 Zm00001d034172 Zm00001d001837 Zm00001d021368 Zm00001d053348 Zm00001d046788 Zm00001d026205 Zm00001d012477 Zm00001d052779 Zm00001d007688 Zm00001d047781 Zm00001d027520 Zm00001d033840 Zm00001d046352 Zm00001d038378 Zm00001d029020 Zm00001d033077 Zm00001d047303 Zm00001d007185 Zm00001d014794 Zm00001d024294 Zm00001d045544 Zm00001d048563 Zm00001d020020 Zm00001d034885 Zm00001d004990 Zm00001d014435 Zm00001d013873 Zm00001d038537 Zm00001d039874 Zm00001d028550 Zm00001d027386 Zm00001d012237 Zm00001d042847 Zm00001d037521 Zm00001d050285 Zm00001d021450 Zm00001d013706 Zm00001d035157 Zm00001d037079 Zm00001d047025 Zm00001d025940 Zm00001d006866 Zm00001d053674 Zm00001d011167 Zm00001d019000 Zm00001d053425 Zm00001d003309 Zm00001d048023 Zm00001d046490 Zm00001d026521 Zm00001d036499 Zm00001d042278 Zm00001d049724 Zm00001d027580 Zm00001d046499 Zm00001d021639 Zm00001d050103 Zm00001d051504 Zm00001d014919 Zm00001d049042 Zm00001d014073 Zm00001d003603 Zm00001d045194 Zm00001d007716 Zm00001d046357 Zm00001d039146 Zm00001d036148 Zm00001d040253 Zm00001d007822 Zm00001d018135 Zm00001d040360 Zm00001d002131 Zm00001d017617 Zm00001d002953 Zm00001d044429 Zm00001d028153 Zm00001d045216 Zm00001d039685 Zm00001d036918 Zm00001d042344 Zm00001d026630 Zm00001d033799 Zm00001d020975 Zm00001d051055 Zm00001d034218 Zm00001d021623 Zm00001d048077 Zm00001d053092 Zm00001d014991 Zm00001d025734 Zm00001d028954 Zm00001d032153 Zm00001d004095 Zm00001d048600 Zm00001d004739 Zm00001d045174 Zm00001d007531 Zm00001d009579 Zm00001d023265 Zm00001d016755 Zm00001d021231 Zm00001d013517 Zm00001d046121 Zm00001d011534 Zm00001d049952 Zm00001d026451 Zm00001d018628 Zm00001d043653 Zm00001d039221 Zm00001d012564 Zm00001d026402 Zm00001d049240 Zm00001d005584 Zm00001d038805 Zm00001d017070 Zm00001d002066 Zm00001d034523 Zm00001d021752 Zm00001d038977 Zm00001d027733 Zm00001d031870 Zm00001d044086 Zm00001d029968 Zm00001d052565 Zm00001d024941 Zm00001d045230 Zm00001d046200 Zm00001d002549 Zm00001d053807 Zm00001d024718 Zm00001d020348 Zm00001d003155 Zm00001d028214 Zm00001d032338 Zm00001d035616 Zm00001d010039 Zm00001d037277 Zm00001d025794 Zm00001d027732 Zm00001d032370 Zm00001d047990 Zm00001d006548 Zm00001d039420 Zm00001d002562 Zm00001d031747 Zm00001d026569 Zm00001d002446 Zm00001d051103 Zm00001d049666 Zm00001d018399 Zm00001d005023 Zm00001d015870 Zm00001d022236 Zm00001d044939 Zm00001d012243 Zm00001d027434 Zm00001d014880 Zm00001d046629 Zm00001d006246 Zm00001d053024 Zm00001d026685 Zm00001d002454 Zm00001d022340 Zm00001d027403 Zm00001d006193 Zm00001d028341 Zm00001d038724 Zm00001d045386 Zm00001d019354 Zm00001d039568 Zm00001d010007 Zm00001d029908 Zm00001d040848 Zm00001d018290 Zm00001d002232 Zm00001d001834 Zm00001d015194 Zm00001d031637 Zm00001d030944 Zm00001d021192 Zm00001d017553 Zm00001d015061 Zm00001d017047 Zm00001d025819 Zm00001d033473 Zm00001d040544 Zm00001d033549 Zm00001d029674 Zm00001d006539 Zm00001d032562 Zm00001d045043 Zm00001d003663 Zm00001d040720 Zm00001d022505 Zm00001d041119 Zm00001d042993 Zm00001d030149 Zm00001d043446 Zm00001d009890 Zm00001d037797 Zm00001d007830 Zm00001d023652 Zm00001d040238 Zm00001d025846 Zm00001d039527 Zm00001d006667 Zm00001d025310 Zm00001d011649 Zm00001d030282 Zm00001d019125 Zm00001d009196 Zm00001d004716 Zm00001d019546 Zm00001d020647 Zm00001d032897 Zm00001d025952 Zm00001d021445 Zm00001d022188 Zm00001d007193 Zm00001d025480 Zm00001d011780 Zm00001d023601 Zm00001d041443 Zm00001d053624 Zm00001d029736 Zm00001d019515 Zm00001d013079 Zm00001d049933 Zm00001d018469 Zm00001d050961 Zm00001d047422 Zm00001d014969 Zm00001d027429 Zm00001d003618 Zm00001d012047 Zm00001d044422 Zm00001d040689 Zm00001d051617 Zm00001d018185 Zm00001d035494 Zm00001d008782 Zm00001d047713 Zm00001d017920 Zm00001d011418 Zm00001d046959 Zm00001d033933 Zm00001d045266 Zm00001d045845 Zm00001d034859 Zm00001d053974 Zm00001d043026 Zm00001d026176 Zm00001d015242 Zm00001d040172 Zm00001d002678 Zm00001d013592 Zm00001d028105 Zm00001d015785 Zm00001d032222 Zm00001d051804 Zm00001d018659 Zm00001d003438 Zm00001d030146 Zm00001d026064 Zm00001d010152 Zm00001d005003 Zm00001d046500 Zm00001d022427 Zm00001d051790 Zm00001d044367 Zm00001d040201 Zm00001d022466 Zm00001d014703 Zm00001d013752 Zm00001d009383 Zm00001d037734 Zm00001d041751 Zm00001d046169 Zm00001d035658 Zm00001d021421 Zm00001d046803 Zm00001d043086 Zm00001d016305 Zm00001d018998 Zm00001d018004 Zm00001d003584 Zm00001d004361 Zm00001d045069 Zm00001d046372 Zm00001d025166 Zm00001d039102 Zm00001d012308 Zm00001d009580 Zm00001d038151 Zm00001d016740 Zm00001d047787 Zm00001d032909 Zm00001d004839 Zm00001d036123 Zm00001d028373 Zm00001d016826 Zm00001d033990 Zm00001d034428 Zm00001d009908 Zm00001d051453 Zm00001d033480 Zm00001d006904 Zm00001d020639 Zm00001d037813 Zm00001d003500 Zm00001d047760 Zm00001d014126 Zm00001d008405 Zm00001d047966 Zm00001d027896 Zm00001d038508 Zm00001d008613 Zm00001d008545 Zm00001d013066 Zm00001d027267 Zm00001d038970 Zm00001d013933 Zm00001d028586 Zm00001d040291 Zm00001d018157 Zm00001d011825 Zm00001d047271 Zm00001d041575 Zm00001d042245 Zm00001d017150 Zm00001d021061 Zm00001d027841 Zm00001d030593 Zm00001d044008 Zm00001d038562 Zm00001d006440 Zm00001d022604 Zm00001d039446 Zm00001d018074 Zm00001d025221 Zm00001d013493 Zm00001d044464 Zm00001d008310 Zm00001d027242 Zm00001d043407 Zm00001d044527 Zm00001d023470 Zm00001d025011 Zm00001d035990 Zm00001d047697 Zm00001d004997 Zm00001d053863 Zm00001d028742 Zm00001d046510 Zm00001d005456 Zm00001d017079 Zm00001d032280 Zm00001d005840 Zm00001d021839 Zm00001d051254 Zm00001d028971 Zm00001d005313 Zm00001d045668 Zm00001d044203 Zm00001d040827 Zm00001d022160 Zm00001d020695 Zm00001d004591 Zm00001d006945 Zm00001d045819 Zm00001d008374 Zm00001d022096 Zm00001d015210 Zm00001d012779 Zm00001d019404 Zm00001d038374 Zm00001d053374 Zm00001d044677 Zm00001d032560 Zm00001d022045 Zm00001d003672 Zm00001d039454 Zm00001d041719 Zm00001d024421 Zm00001d052011 Zm00001d021704 Zm00001d034439 Zm00001d038909 Zm00001d014868 Zm00001d043696 Zm00001d052937 Zm00001d009792 Zm00001d052921 Zm00001d023217 Zm00001d052551 Zm00001d018511 Zm00001d043432 Zm00001d010466 Zm00001d008357 Zm00001d011967 Zm00001d012862 Zm00001d002296 Zm00001d035180 Zm00001d009640 Zm00001d013656 Zm00001d046898 Zm00001d038366 Zm00001d049006 Zm00001d028754 Zm00001d022595 Zm00001d040760 Zm00001d039787 Zm00001d005114 Zm00001d020563 Zm00001d012937 Zm00001d019754 Zm00001d034627 Zm00001d045202 Zm00001d047666 Zm00001d020325 Zm00001d032819 Zm00001d049367 Zm00001d053613 Zm00001d028471 Zm00001d026609 Zm00001d009271 Zm00001d042663 Zm00001d028352 Zm00001d028432 Zm00001d009465 Zm00001d039576 Zm00001d031269 Zm00001d049348 Zm00001d031453 Zm00001d021545 Zm00001d039611 Zm00001d035054 Zm00001d052031 Zm00001d044513 Zm00001d011005 Zm00001d026404 Zm00001d008225 Zm00001d034561 Zm00001d003954 Zm00001d039669 Zm00001d021536 Zm00001d019775 Zm00001d026650 Zm00001d040341 Zm00001d026536 Zm00001d041969 Zm00001d021465 Zm00001d022313 Zm00001d022242 Zm00001d020471 Zm00001d006673 Zm00001d023655 Zm00001d029234 Zm00001d041994 Zm00001d029592 Zm00001d004551 Zm00001d050411 Zm00001d046656 Zm00001d047255 Zm00001d049320 Zm00001d017979 Zm00001d039709 Zm00001d048529 Zm00001d014078 Zm00001d043270 Zm00001d017912 Zm00001d027556 Zm00001d002966 Zm00001d018647 Zm00001d034937 Zm00001d034649 Zm00001d034007 Zm00001d018131 Zm00001d018966 Zm00001d028453 Zm00001d003143 Zm00001d004812 Zm00001d004690 Zm00001d049764 Zm00001d021282 Zm00001d037741 Zm00001d022450 Zm00001d008757 Zm00001d050269 Zm00001d016436 Zm00001d028191 Zm00001d035050 Zm00001d032427 Zm00001d010278 Zm00001d006100 Zm00001d013329 Zm00001d035195 Zm00001d011182 Zm00001d018954 Zm00001d053087 Zm00001d013375 Zm00001d031893 Zm00001d049582 Zm00001d039007 Zm00001d034793 Zm00001d029036 Zm00001d038191 Zm00001d015212 Zm00001d007594 Zm00001d014367 Zm00001d029530 Zm00001d009776 Zm00001d046855 Zm00001d013369 Zm00001d016101 Zm00001d040314 Zm00001d042479 Zm00001d041814 Zm00001d013196 Zm00001d017783 Zm00001d014330 Zm00001d019898 Zm00001d051060 Zm00001d006738 Zm00001d030867 Zm00001d053901 Zm00001d048614 Zm00001d051203 Zm00001d026405 Zm00001d051455 Zm00001d038126 Zm00001d017647 Zm00001d007599 Zm00001d051244 Zm00001d032543 Zm00001d013339 Zm00001d031002 Zm00001d025319 Zm00001d032497 Zm00001d007234 Zm00001d053064 Zm00001d021835 Zm00001d016410 Zm00001d017889 Zm00001d031184 Zm00001d018096 Zm00001d002889 Zm00001d003878 Zm00001d018278 Zm00001d047396 Zm00001d049788 Zm00001d030148 Zm00001d029841 Zm00001d006505 Zm00001d002395 Zm00001d022274 Zm00001d035443 Zm00001d005018 Zm00001d045919 Zm00001d047940 Zm00001d042500 Zm00001d014328 Zm00001d047907 Zm00001d039289 Zm00001d026020 Zm00001d051670 Zm00001d013528 Zm00001d042601 Zm00001d015124 Zm00001d039729 Zm00001d009803 Zm00001d028569 Zm00001d022546 Zm00001d012888 Zm00001d009556 Zm00001d050637 Zm00001d028221 Zm00001d031963 Zm00001d017590 Zm00001d002369 Zm00001d051421 Zm00001d006447 Zm00001d034453 Zm00001d033288 Zm00001d045199 Zm00001d043366 Zm00001d018380 Zm00001d049676 Zm00001d036451 Zm00001d026574 Zm00001d004917 Zm00001d018826 Zm00001d039253 Zm00001d037422 Zm00001d017995 Zm00001d044916 Zm00001d039647 Zm00001d048834 Zm00001d031014 Zm00001d005172 Zm00001d048311 Zm00001d011846 Zm00001d005586 Zm00001d006861 Zm00001d052810 Zm00001d038855 Zm00001d015445 Zm00001d006328 Zm00001d040959 Zm00001d044838 Zm00001d019222 Zm00001d038904 Zm00001d050190 Zm00001d053053 Zm00001d049369 Zm00001d025694 Zm00001d005270 Zm00001d033670 Zm00001d026467 Zm00001d029215 Zm00001d049326 Zm00001d025421 Zm00001d035063 Zm00001d025359 Zm00001d052978 Zm00001d042808 Zm00001d045650 Zm00001d036521 Zm00001d044170 Zm00001d002824 Zm00001d019988 Zm00001d006931 Zm00001d045935 Zm00001d037573 Zm00001d015965 Zm00001d026096 Zm00001d041945 Zm00001d022110 Zm00001d016041 Zm00001d033744 Zm00001d050698 Zm00001d042474 Zm00001d008212 Zm00001d051674 Zm00001d037480 Zm00001d012573 Zm00001d037487 Zm00001d027266 Zm00001d042729 Zm00001d021224 Zm00001d043565 Zm00001d003426 Zm00001d049823 Zm00001d048126 Zm00001d044668 Zm00001d034432 Zm00001d012831 Zm00001d022608 Zm00001d017953 Zm00001d039719 Zm00001d037181 Zm00001d010625 Zm00001d002113 Zm00001d015251 Zm00001d025923 Zm00001d012875 Zm00001d003021 Zm00001d042250 Zm00001d048441 Zm00001d018111 Zm00001d049965 Zm00001d035256 Zm00001d052319 Zm00001d019522 Zm00001d013317 Zm00001d040500 Zm00001d025957 Zm00001d012633 Zm00001d034699 Zm00001d003275 Zm00001d044246 Zm00001d006388 Zm00001d039816 Zm00001d047714 Zm00001d013348 Zm00001d053751 Zm00001d008582 Zm00001d015449 Zm00001d018410 Zm00001d028472 Zm00001d026573 Zm00001d025745 Zm00001d015572 Zm00001d039337 Zm00001d013300 Zm00001d003025 Zm00001d048814 Zm00001d030094 Zm00001d041918 Zm00001d040596 Zm00001d019057 Zm00001d007600 Zm00001d034726 Zm00001d013238 Zm00001d009645 Zm00001d049381 Zm00001d034692 Zm00001d052108 Zm00001d018401 Zm00001d009186 Zm00001d052798 Zm00001d024595 Zm00001d033291 Zm00001d027318 Zm00001d032521 Zm00001d034298 Zm00001d039660 Zm00001d027329 Zm00001d005110 Zm00001d053635 Zm00001d053377 Zm00001d021682 Zm00001d019247 Zm00001d041703 Zm00001d053698 Zm00001d004841 Zm00001d034514 Zm00001d034628 Zm00001d032540 Zm00001d037893 Zm00001d043153 Zm00001d009171 Zm00001d010476 Zm00001d019587 Zm00001d006761 Zm00001d013861 Zm00001d008858 Zm00001d003784 Zm00001d022476 Zm00001d039956 Zm00001d011773 Zm00001d015140 Zm00001d018755 Zm00001d044931 Zm00001d017469 Zm00001d024928 Zm00001d049731 Zm00001d045567 Zm00001d030399 Zm00001d052269 Zm00001d025554 Zm00001d012537 Zm00001d053643 Zm00001d043070 Zm00001d024978 Zm00001d005622 Zm00001d009673 Zm00001d025706 Zm00001d013816 Zm00001d051794 Zm00001d042842 Zm00001d011992 Zm00001d013836 Zm00001d044350 Zm00001d026089 Zm00001d047852 Zm00001d025977 Zm00001d011362 Zm00001d011108 Zm00001d026322 Zm00001d004868 Zm00001d047262 Zm00001d017709 Zm00001d018276 Zm00001d040542 Zm00001d029848 Zm00001d047667 Zm00001d045381 Zm00001d045505 Zm00001d039427 Zm00001d054038 Zm00001d040588 Zm00001d051472 Zm00001d008791 Zm00001d051065 Zm00001d038270 Zm00001d046940 Zm00001d052212 Zm00001d037701 Zm00001d029033 Zm00001d034718 Zm00001d025589 Zm00001d028981 Zm00001d045304 Zm00001d012923 Zm00001d005179 Zm00001d002632 Zm00001d001979 Zm00001d033657 Zm00001d048193 Zm00001d044815 Zm00001d048914 Zm00001d046929 Zm00001d012266 Zm00001d028641 Zm00001d012367 Zm00001d030954 Zm00001d018828 Zm00001d034486 Zm00001d043534 Zm00001d031677 Zm00001d045027 Zm00001d040204 Zm00001d040438 Zm00001d043998 Zm00001d042519 Zm00001d010500 Zm00001d053020 Zm00001d034522 Zm00001d039401 Zm00001d047267 Zm00001d046970 Zm00001d035584 Zm00001d047918 Zm00001d021528 Zm00001d005344 Zm00001d047218 Zm00001d048362 Zm00001d032257 Zm00001d016865 Zm00001d043183 Zm00001d043735 Zm00001d012830 Zm00001d042480 Zm00001d033226 Zm00001d033181 Zm00001d012810 Zm00001d015383 Zm00001d034035 Zm00001d040169 Zm00001d030218 Zm00001d017395 Zm00001d021686 Zm00001d038301 Zm00001d013914 Zm00001d045430 Zm00001d002032 Zm00001d029868 Zm00001d038653 Zm00001d027498 Zm00001d012121 Zm00001d016470 Zm00001d036989 Zm00001d027648 Zm00001d028428 Zm00001d011122 Zm00001d031764 Zm00001d006274 Zm00001d010137 Zm00001d006212 Zm00001d032181 Zm00001d040513 Zm00001d012168 Zm00001d048233 Zm00001d048924 Zm00001d010867 Zm00001d039650 Zm00001d031975 Zm00001d043354 Zm00001d038841 Zm00001d031241 Zm00001d037737 Zm00001d027827 Zm00001d020738 Zm00001d050666 Zm00001d011075 Zm00001d039142 Zm00001d053672 Zm00001d015415 Zm00001d002601 Zm00001d048496 Zm00001d048585 Zm00001d009520 Zm00001d008744 Zm00001d023447 Zm00001d005732 Zm00001d046967 Zm00001d035761 Zm00001d043410 Zm00001d001881 Zm00001d040075 Zm00001d041831 Zm00001d022544 Zm00001d010566 Zm00001d045926 Zm00001d027435 Zm00001d001787 Zm00001d028770 Zm00001d019338 Zm00001d018786 Zm00001d004248 Zm00001d019857 Zm00001d041678 Zm00001d014882 Zm00001d043485 Zm00001d037067 Zm00001d039840 Zm00001d004052 Zm00001d029722 Zm00001d026137 Zm00001d045565 Zm00001d033099 Zm00001d037235 Zm00001d048698 Zm00001d015058 Zm00001d021642 Zm00001d028414 Zm00001d043484 Zm00001d028549 Zm00001d051967 Zm00001d038658 Zm00001d002789 Zm00001d053625 Zm00001d039929 Zm00001d014744 Zm00001d028744 Zm00001d019759 Zm00001d017491 Zm00001d039893 Zm00001d036608 Zm00001d037120 Zm00001d032423 Zm00001d034183 Zm00001d043389 Zm00001d019648 Zm00001d042329 Zm00001d029195 Zm00001d010016 Zm00001d048483 Zm00001d051082 Zm00001d020463 Zm00001d004130 Zm00001d006587 Zm00001d025847 Zm00001d027383 Zm00001d020713 Zm00001d006063 Zm00001d005769 Zm00001d047749 Zm00001d021751 Zm00001d042685 Zm00001d024980 Zm00001d004910 Zm00001d052020 Zm00001d003205 Zm00001d005140 Zm00001d009950 Zm00001d029390 Zm00001d036318 Zm00001d022355 Zm00001d009007 Zm00001d021494 Zm00001d015549 Zm00001d021300 Zm00001d038925 Zm00001d053964 Zm00001d021291 Zm00001d026060 Zm00001d033227 Zm00001d021757 Zm00001d052165 Zm00001d012936 Zm00001d045618 Zm00001d053932 Zm00001d051544 Zm00001d033913 Zm00001d008387 Zm00001d037937 Zm00001d011611 Zm00001d028357 Zm00001d032164 Zm00001d034933 Zm00001d014401 Zm00001d009548 Zm00001d042131 Zm00001d037205 Zm00001d017622 Zm00001d019733 Zm00001d008224 Zm00001d048135 Zm00001d034420 Zm00001d003349 Zm00001d047462 Zm00001d029246 Zm00001d010650 Zm00001d053716 Zm00001d045574 Zm00001d003643 Zm00001d016072 Zm00001d043851 Zm00001d043009 Zm00001d018660 Zm00001d010053 Zm00001d025911 Zm00001d013468 Zm00001d043118 Zm00001d015568 Zm00001d047236 Zm00001d032636 Zm00001d048451 Zm00001d053632 Zm00001d049153 Zm00001d051564 Zm00001d032531 Zm00001d021815 Zm00001d011966 Zm00001d012141 Zm00001d053835 Zm00001d035201 Zm00001d020667 Zm00001d011525 Zm00001d034448 Zm00001d007474 Zm00001d023651 Zm00001d006521 Zm00001d007835 Zm00001d039994 Zm00001d016787 Zm00001d032311 Zm00001d030638 Zm00001d023629 Zm00001d012992 Zm00001d033111 Zm00001d013999 Zm00001d019449 Zm00001d029673 Zm00001d037832 Zm00001d011103 Zm00001d035013 Zm00001d007422 Zm00001d002312 Zm00001d021370 Zm00001d024710 Zm00001d025624 Zm00001d013359 Zm00001d005583 Zm00001d014759 Zm00001d026603 Zm00001d034666 Zm00001d031300 Zm00001d014715 Zm00001d030920 Zm00001d049554 Zm00001d048825 Zm00001d050915 Zm00001d031927 Zm00001d005649 Zm00001d036917 Zm00001d032763 Zm00001d014417 Zm00001d012479 Zm00001d044607 Zm00001d018890 Zm00001d043418 Zm00001d042052 Zm00001d036079 Zm00001d024527 Zm00001d029696 Zm00001d009522 Zm00001d008641 Zm00001d025476 Zm00001d008649 Zm00001d026557 Zm00001d041777 Zm00001d038980 Zm00001d046210 Zm00001d035377 Zm00001d047467 Zm00001d043863 Zm00001d018982 Zm00001d025752 Zm00001d037993 Zm00001d019613 Zm00001d009762 Zm00001d034524 Zm00001d053705 Zm00001d021060 Zm00001d008407 Zm00001d012802 Zm00001d006769 Zm00001d018810 Zm00001d037675 Zm00001d004075 Zm00001d051588 Zm00001d003146 Zm00001d032083 Zm00001d032744 Zm00001d008360 Zm00001d001830 Zm00001d048987 Zm00001d003411 Zm00001d048521 Zm00001d035515 Zm00001d030677 Zm00001d011691 Zm00001d011278 Zm00001d017487 Zm00001d012891 Zm00001d045096 Zm00001d049260 Zm00001d047793 Zm00001d046946 Zm00001d017470 Zm00001d016874 Zm00001d051474 Zm00001d012741 Zm00001d018282 Zm00001d013067 Zm00001d041691 Zm00001d032337 Zm00001d039070 Zm00001d014497 Zm00001d051387 Zm00001d031640 Zm00001d054047 Zm00001d032668 Zm00001d019312 Zm00001d003252 Zm00001d016915 Zm00001d005457 Zm00001d005253 Zm00001d025901 Zm00001d053104 Zm00001d042772 Zm00001d027483 Zm00001d053801 Zm00001d009364 Zm00001d031213 Zm00001d023592 Zm00001d035171 Zm00001d032379 Zm00001d003671 Zm00001d045447 Zm00001d043767 Zm00001d007650 Zm00001d040257 Zm00001d007932 Zm00001d011646 Zm00001d041504 Zm00001d049170 Zm00001d029376 Zm00001d047909 Zm00001d007892 Zm00001d005614 Zm00001d019102 Zm00001d018634 Zm00001d048198 Zm00001d052648 Zm00001d050173 Zm00001d006620 Zm00001d013455 Zm00001d015747 Zm00001d032775 Zm00001d042609 Zm00001d043632 Zm00001d047124 Zm00001d011757 Zm00001d020487 Zm00001d002106 Zm00001d008900 Zm00001d018030 Zm00001d045800 Zm00001d003462 Zm00001d022243 Zm00001d006320 Zm00001d025953 Zm00001d015399 Zm00001d013431 Zm00001d025875 Zm00001d034853 Zm00001d008820 Zm00001d040185 Zm00001d011104 Zm00001d050428 Zm00001d022513 Zm00001d046887 Zm00001d016106 Zm00001d013583 Zm00001d024715 Zm00001d006944 Zm00001d033619 Zm00001d008289 Zm00001d006717 Zm00001d004386 Zm00001d006474 Zm00001d042861 Zm00001d002485 Zm00001d011938 Zm00001d044208 Zm00001d006309 Zm00001d007025 Zm00001d052621 Zm00001d017121 Zm00001d019260 Zm00001d029039 Zm00001d013056 Zm00001d044390 Zm00001d039081 Zm00001d036201 Zm00001d007109 Zm00001d019671 Zm00001d014844 Zm00001d031532 Zm00001d043073 Zm00001d017216 Zm00001d053658 Zm00001d028779 Zm00001d026651 Zm00001d028748 Zm00001d051611 Zm00001d011136 Zm00001d037343 Zm00001d030285 Zm00001d042128 Zm00001d022400 Zm00001d020603 Zm00001d036993 Zm00001d049347 Zm00001d006168 Zm00001d031961 Zm00001d038226 Zm00001d030496 Zm00001d011178 Zm00001d039575 Zm00001d018406 Zm00001d034838 Zm00001d017306 Zm00001d030895 Zm00001d047612 Zm00001d014272 Zm00001d021667 Zm00001d002036 Zm00001d052258 Zm00001d034336 Zm00001d011486 Zm00001d042341 Zm00001d021729 Zm00001d012323 Zm00001d013283 Zm00001d044459 Zm00001d006821 Zm00001d049043 Zm00001d040679 Zm00001d021958 Zm00001d012878 Zm00001d024720 Zm00001d037778 Zm00001d050146 Zm00001d023462 Zm00001d001870 Zm00001d006390 Zm00001d045146 Zm00001d004426 Zm00001d014853 Zm00001d002231 Zm00001d031703 Zm00001d007420 Zm00001d013159 Zm00001d005012 Zm00001d022144 Zm00001d022141 Zm00001d048606 Zm00001d011210 Zm00001d004193 Zm00001d015553 Zm00001d009618 Zm00001d039073 Zm00001d040953 Zm00001d015804 Zm00001d025622 Zm00001d002597 Zm00001d011063 Zm00001d044185 Zm00001d020402 Zm00001d016898 Zm00001d044382 Zm00001d031611 Zm00001d022565 Zm00001d048292 Zm00001d036795 Zm00001d037818 Zm00001d049190 Zm00001d020997 Zm00001d019223 Zm00001d003376 Zm00001d025423 Zm00001d005334 Zm00001d018482 Zm00001d034018 Zm00001d002429 Zm00001d004731 Zm00001d044415 Zm00001d003254 Zm00001d047554 Zm00001d010711 Zm00001d008406 Zm00001d025757 Zm00001d038961 Zm00001d030967 Zm00001d005459 Zm00001d026369 Zm00001d048104 Zm00001d022364 Zm00001d002155 Zm00001d027468 Zm00001d018141 Zm00001d041327 Zm00001d047967 Zm00001d026334 Zm00001d043095 Zm00001d050920 Zm00001d044819 Zm00001d012800 Zm00001d017767 Zm00001d039417 Zm00001d033295 Zm00001d018873 Zm00001d042600 Zm00001d041076 Zm00001d006907 Zm00001d008193 Zm00001d041734 Zm00001d038510 Zm00001d005089 Zm00001d040290 Zm00001d016411 Zm00001d040034 Zm00001d017833 Zm00001d035916 |
| Zhengzhou Harbin | 598 | Zm00001d033896 Zm00001d007457 Zm00001d037010 Zm00001d029201 Zm00001d002063 Zm00001d049485 Zm00001d043174 Zm00001d026361 Zm00001d022480 Zm00001d028813 Zm00001d053960 Zm00001d038172 Zm00001d046272 Zm00001d036023 Zm00001d034879 Zm00001d029105 Zm00001d030573 Zm00001d012146 Zm00001d025749 Zm00001d002514 Zm00001d028958 Zm00001d002490 Zm00001d005848 Zm00001d026277 Zm00001d051885 Zm00001d017812 Zm00001d033136 Zm00001d043215 Zm00001d006488 Zm00001d008646 Zm00001d040571 Zm00001d038273 Zm00001d040846 Zm00001d008691 Zm00001d031561 Zm00001d051881 Zm00001d037318 Zm00001d012868 Zm00001d037499 Zm00001d011459 Zm00001d027597 Zm00001d006327 Zm00001d051824 Zm00001d043942 Zm00001d047217 Zm00001d040435 Zm00001d043580 Zm00001d037599 Zm00001d043088 Zm00001d005826 Zm00001d011006 Zm00001d014770 Zm00001d030652 Zm00001d013985 Zm00001d038809 Zm00001d038881 Zm00001d029948 Zm00001d047385 Zm00001d044730 Zm00001d035762 Zm00001d010126 Zm00001d006213 Zm00001d042690 Zm00001d051910 Zm00001d047857 Zm00001d008622 Zm00001d013188 Zm00001d032035 Zm00001d052985 Zm00001d031118 Zm00001d028670 Zm00001d039520 Zm00001d024462 Zm00001d010211 Zm00001d044500 Zm00001d033705 Zm00001d030821 Zm00001d025843 Zm00001d018328 Zm00001d028999 Zm00001d031062 Zm00001d040857 Zm00001d012396 Zm00001d043165 Zm00001d040691 Zm00001d046304 Zm00001d039559 Zm00001d052087 Zm00001d033172 Zm00001d021999 Zm00001d006043 Zm00001d025628 Zm00001d033388 Zm00001d038400 Zm00001d009840 Zm00001d005587 Zm00001d019173 Zm00001d006033 Zm00001d034657 Zm00001d012484 Zm00001d028622 Zm00001d020642 Zm00001d047175 Zm00001d007638 Zm00001d031700 Zm00001d032283 Zm00001d030421 Zm00001d003159 Zm00001d015509 Zm00001d034251 Zm00001d021947 Zm00001d044266 Zm00001d047420 Zm00001d040733 Zm00001d045315 Zm00001d037239 Zm00001d020283 Zm00001d003256 Zm00001d028568 Zm00001d005032 Zm00001d013565 Zm00001d052472 Zm00001d003974 Zm00001d041064 Zm00001d025793 Zm00001d029560 Zm00001d047522 Zm00001d002419 Zm00001d007810 Zm00001d042347 Zm00001d026097 Zm00001d053177 Zm00001d018798 Zm00001d043834 Zm00001d025080 Zm00001d028668 Zm00001d005951 Zm00001d007105 Zm00001d009330 Zm00001d005833 Zm00001d048497 Zm00001d046445 Zm00001d005817 Zm00001d006037 Zm00001d034038 Zm00001d005480 Zm00001d015703 Zm00001d011823 Zm00001d036959 Zm00001d038208 Zm00001d009570 Zm00001d037574 Zm00001d031505 Zm00001d050430 Zm00001d012362 Zm00001d002965 Zm00001d038981 Zm00001d043500 Zm00001d005807 Zm00001d007961 Zm00001d042874 Zm00001d032989 Zm00001d011570 Zm00001d026386 Zm00001d037902 Zm00001d049738 Zm00001d011634 Zm00001d019185 Zm00001d049479 Zm00001d046923 Zm00001d016084 Zm00001d047283 Zm00001d006725 Zm00001d039411 Zm00001d021805 Zm00001d049094 Zm00001d019520 Zm00001d040503 Zm00001d037481 Zm00001d050293 Zm00001d048034 Zm00001d029004 Zm00001d041803 Zm00001d002307 Zm00001d008985 Zm00001d040408 Zm00001d035720 Zm00001d046378 Zm00001d031163 Zm00001d008285 Zm00001d038838 Zm00001d012972 Zm00001d046322 Zm00001d007162 Zm00001d006342 Zm00001d010425 Zm00001d025573 Zm00001d053397 Zm00001d030194 Zm00001d003176 Zm00001d010321 Zm00001d008854 Zm00001d033218 Zm00001d039505 Zm00001d031832 Zm00001d036759 Zm00001d018781 Zm00001d025715 Zm00001d045877 Zm00001d017360 Zm00001d048634 Zm00001d010534 Zm00001d050917 Zm00001d038752 Zm00001d042091 Zm00001d016145 Zm00001d034109 Zm00001d031986 Zm00001d015571 Zm00001d011851 Zm00001d037195 Zm00001d027576 Zm00001d024605 Zm00001d024049 Zm00001d045631 Zm00001d047776 Zm00001d025963 Zm00001d034490 Zm00001d007287 Zm00001d036961 Zm00001d029050 Zm00001d009708 Zm00001d039697 Zm00001d048551 Zm00001d027446 Zm00001d015526 Zm00001d050300 Zm00001d018893 Zm00001d002971 Zm00001d036768 Zm00001d012672 Zm00001d016640 Zm00001d026668 Zm00001d039379 Zm00001d020233 Zm00001d034805 Zm00001d043443 Zm00001d043059 Zm00001d002174 Zm00001d030184 Zm00001d050310 Zm00001d030048 Zm00001d014157 Zm00001d038606 Zm00001d026606 Zm00001d033053 Zm00001d022172 Zm00001d029266 Zm00001d049210 Zm00001d011879 Zm00001d007571 Zm00001d023692 Zm00001d028755 Zm00001d051742 Zm00001d028801 Zm00001d048736 Zm00001d032836 Zm00001d028431 Zm00001d032027 Zm00001d003635 Zm00001d009476 Zm00001d053650 Zm00001d050283 Zm00001d005981 Zm00001d034564 Zm00001d027876 Zm00001d001924 Zm00001d032126 Zm00001d053300 Zm00001d046391 Zm00001d028005 Zm00001d034414 Zm00001d028035 Zm00001d003284 Zm00001d017093 Zm00001d023674 Zm00001d006628 Zm00001d035367 Zm00001d031684 Zm00001d023331 Zm00001d038205 Zm00001d037768 Zm00001d036551 Zm00001d010189 Zm00001d022600 Zm00001d019122 Zm00001d054067 Zm00001d048133 Zm00001d032318 Zm00001d023791 Zm00001d046534 Zm00001d045263 Zm00001d018444 Zm00001d028114 Zm00001d028905 Zm00001d046654 Zm00001d027676 Zm00001d019586 Zm00001d001988 Zm00001d016535 Zm00001d032386 Zm00001d034080 Zm00001d019534 Zm00001d002810 Zm00001d007192 Zm00001d053628 Zm00001d021314 Zm00001d011517 Zm00001d007686 Zm00001d023423 Zm00001d009352 Zm00001d028020 Zm00001d014632 Zm00001d003469 Zm00001d029849 Zm00001d033906 Zm00001d003762 Zm00001d010859 Zm00001d031777 Zm00001d039441 Zm00001d034948 Zm00001d041510 Zm00001d008332 Zm00001d040305 Zm00001d042845 Zm00001d051245 Zm00001d031819 Zm00001d052186 Zm00001d049230 Zm00001d039831 Zm00001d028186 Zm00001d007776 Zm00001d049662 Zm00001d023523 Zm00001d046706 Zm00001d012074 Zm00001d043606 Zm00001d023704 Zm00001d022625 Zm00001d028417 Zm00001d030688 Zm00001d032481 Zm00001d048080 Zm00001d038765 Zm00001d048013 Zm00001d041287 Zm00001d043743 Zm00001d003674 Zm00001d029758 Zm00001d042207 Zm00001d007319 Zm00001d020615 Zm00001d015851 Zm00001d028359 Zm00001d051968 Zm00001d031490 Zm00001d028189 Zm00001d009775 Zm00001d005271 Zm00001d036626 Zm00001d005250 Zm00001d043943 Zm00001d038852 Zm00001d050823 Zm00001d036261 Zm00001d006112 Zm00001d014597 Zm00001d042841 Zm00001d040599 Zm00001d032148 Zm00001d025104 Zm00001d026689 Zm00001d011051 Zm00001d049835 Zm00001d002928 Zm00001d009411 Zm00001d015374 Zm00001d050020 Zm00001d029227 Zm00001d028740 Zm00001d012856 Zm00001d003265 Zm00001d003667 Zm00001d041861 Zm00001d010679 Zm00001d013420 Zm00001d034766 Zm00001d024204 Zm00001d006710 Zm00001d004576 Zm00001d017890 Zm00001d037620 Zm00001d017845 Zm00001d046684 Zm00001d038166 Zm00001d033633 Zm00001d007042 Zm00001d031525 Zm00001d029706 Zm00001d053539 Zm00001d018916 Zm00001d039328 Zm00001d010493 Zm00001d003390 Zm00001d052892 Zm00001d002602 Zm00001d034066 Zm00001d025339 Zm00001d028597 Zm00001d022273 Zm00001d028228 Zm00001d010251 Zm00001d021770 Zm00001d036091 Zm00001d049234 Zm00001d037700 Zm00001d007930 Zm00001d026103 Zm00001d043880 Zm00001d044458 Zm00001d030761 Zm00001d026457 Zm00001d048542 Zm00001d010411 Zm00001d038974 Zm00001d050208 Zm00001d020434 Zm00001d011767 Zm00001d006916 Zm00001d014280 Zm00001d018247 Zm00001d013678 Zm00001d020972 Zm00001d026312 Zm00001d030609 Zm00001d047184 Zm00001d015493 Zm00001d048089 Zm00001d033707 Zm00001d013444 Zm00001d053200 Zm00001d018013 Zm00001d011224 Zm00001d014587 Zm00001d004333 Zm00001d033954 Zm00001d041696 Zm00001d023912 Zm00001d017874 Zm00001d034357 Zm00001d008531 Zm00001d004976 Zm00001d029064 Zm00001d025545 Zm00001d046717 Zm00001d013471 Zm00001d047565 Zm00001d019075 Zm00001d009409 Zm00001d050179 Zm00001d035149 Zm00001d009919 Zm00001d032565 Zm00001d046033 Zm00001d017791 Zm00001d008200 Zm00001d008439 Zm00001d035014 Zm00001d033334 Zm00001d017691 Zm00001d029955 Zm00001d001947 Zm00001d012982 Zm00001d032336 Zm00001d050483 Zm00001d024424 Zm00001d012083 Zm00001d002344 Zm00001d039481 Zm00001d011387 Zm00001d005961 Zm00001d012823 Zm00001d015886 Zm00001d022438 Zm00001d010723 Zm00001d038927 Zm00001d038503 Zm00001d021187 Zm00001d005695 Zm00001d011121 Zm00001d046948 Zm00001d039366 Zm00001d048017 Zm00001d002371 Zm00001d045075 Zm00001d051980 Zm00001d012550 Zm00001d016463 Zm00001d030786 Zm00001d013111 Zm00001d051600 Zm00001d015956 Zm00001d031639 Zm00001d038689 Zm00001d006670 Zm00001d038811 Zm00001d008432 Zm00001d030627 Zm00001d046554 Zm00001d007241 Zm00001d031621 Zm00001d018040 Zm00001d007067 Zm00001d041355 Zm00001d017261 Zm00001d032994 Zm00001d039386 Zm00001d018772 Zm00001d032081 Zm00001d033589 Zm00001d034166 Zm00001d028656 Zm00001d032692 Zm00001d011840 Zm00001d051180 Zm00001d025240 Zm00001d012568 Zm00001d017717 Zm00001d032226 Zm00001d002366 Zm00001d041407 Zm00001d034406 Zm00001d045176 Zm00001d024382 Zm00001d019246 Zm00001d019041 Zm00001d043395 Zm00001d006125 Zm00001d038931 Zm00001d037501 Zm00001d045233 Zm00001d007158 Zm00001d027722 Zm00001d044606 Zm00001d011325 Zm00001d051362 Zm00001d049959 Zm00001d005966 Zm00001d004855 Zm00001d038100 Zm00001d032617 Zm00001d042216 Zm00001d041474 Zm00001d038291 Zm00001d012546 Zm00001d038754 Zm00001d006437 Zm00001d003024 Zm00001d020712 Zm00001d045431 Zm00001d017390 Zm00001d008517 Zm00001d033346 Zm00001d050942 Zm00001d048239 Zm00001d038189 Zm00001d009714 Zm00001d001846 Zm00001d035957 Zm00001d040274 Zm00001d014949 Zm00001d010047 Zm00001d002000 Zm00001d034781 Zm00001d029516 Zm00001d029232 Zm00001d033886 Zm00001d048948 Zm00001d002896 Zm00001d053404 Zm00001d026541 Zm00001d039264 Zm00001d032109 Zm00001d027626 Zm00001d033060 Zm00001d003283 Zm00001d026031 Zm00001d025626 Zm00001d034050 Zm00001d040855 Zm00001d032571 Zm00001d018822 Zm00001d014011 Zm00001d010294 Zm00001d006295 Zm00001d002100 Zm00001d005507 Zm00001d042734 Zm00001d012892 Zm00001d007966 Zm00001d004512 Zm00001d027637 Zm00001d039538 Zm00001d024435 Zm00001d022254 Zm00001d036247 Zm00001d017502 Zm00001d031328 Zm00001d023506 Zm00001d034813 Zm00001d025340 Zm00001d051496 Zm00001d005711 Zm00001d014668 Zm00001d043122 Zm00001d014903 Zm00001d031954 Zm00001d029331 Zm00001d004543 Zm00001d037415 Zm00001d052941 Zm00001d029197 Zm00001d020764 Zm00001d006070 Zm00001d052893 Zm00001d028422 Zm00001d005442 Zm00001d051765 Zm00001d002825 Zm00001d044762 Zm00001d028898 Zm00001d035527 Zm00001d019363 Zm00001d014692 Zm00001d053158 Zm00001d028475 Zm00001d035185 Zm00001d019731 Zm00001d016591 Zm00001d018468 Zm00001d045665 Zm00001d039090 Zm00001d012274 Zm00001d028287 Zm00001d053798 Zm00001d053783 Zm00001d017557 Zm00001d012261 Zm00001d042917 Zm00001d022134 Zm00001d013342 Zm00001d002695 Zm00001d041679 Zm00001d020367 Zm00001d005784 Zm00001d047537 Zm00001d026280 Zm00001d019576 Zm00001d006158 Zm00001d006059 Zm00001d018994 Zm00001d034897 Zm00001d011972 Zm00001d021065 Zm00001d043569 Zm00001d036720 Zm00001d031315 Zm00001d048544 Zm00001d047922 Zm00001d003653 Zm00001d015205 Zm00001d032815 Zm00001d040192 Zm00001d010195 Zm00001d007802 Zm00001d006227 Zm00001d031098 Zm00001d053991 Zm00001d036428 Zm00001d036564 Zm00001d053697 Zm00001d039380 Zm00001d034107 Zm00001d043445 Zm00001d018049 Zm00001d013319 Zm00001d017384 Zm00001d017777 Zm00001d032424 Zm00001d034824 Zm00001d009337 Zm00001d029286 Zm00001d003937 Zm00001d010422 Zm00001d030357 Zm00001d024939 Zm00001d039179 Zm00001d017675 Zm00001d010265 Zm00001d049461 Zm00001d037503 Zm00001d007396 Zm00001d037490 Zm00001d038860 Zm00001d022238 Zm00001d021974 Zm00001d032449 Zm00001d001903 Zm00001d041603 Zm00001d045015 Zm00001d050913 Zm00001d024694 Zm00001d028901 Zm00001d013496 Zm00001d006679 Zm00001d007381 Zm00001d039306 Zm00001d016786 Zm00001d050161 Zm00001d011975 Zm00001d047453 Zm00001d052803 Zm00001d009511 Zm00001d045615 Zm00001d003712 Zm00001d029886 Zm00001d051375 Zm00001d047121 Zm00001d029264 Zm00001d003434 Zm00001d035597 Zm00001d030533 Zm00001d002024 Zm00001d043141 Zm00001d030995 Zm00001d003358 Zm00001d004282 Zm00001d016831 Zm00001d030725 Zm00001d023499 Zm00001d050741 Zm00001d012731 Zm00001d007897 Zm00001d009952 Zm00001d038268 Zm00001d027293 Zm00001d016129 Zm00001d028689 Zm00001d012040 Zm00001d030939 Zm00001d005019 Zm00001d010491 Zm00001d015723 Zm00001d013120 Zm00001d030561 Zm00001d014973 Zm00001d025016 Zm00001d050640 Zm00001d052462 Zm00001d046971 Zm00001d053870 Zm00001d032062 Zm00001d004389 Zm00001d046755 Zm00001d043954 Zm00001d046681 Zm00001d019104 Zm00001d050616 Zm00001d018887 Zm00001d038572 Zm00001d004782 Zm00001d016490 Zm00001d006219 Zm00001d022152 Zm00001d013531 Zm00001d043490 Zm00001d002347 Zm00001d005107 Zm00001d006922 Zm00001d012110 Zm00001d017460 Zm00001d038732 Zm00001d021609 Zm00001d016530 Zm00001d008975 Zm00001d040517 Zm00001d047941 Zm00001d034857 Zm00001d051399 Zm00001d040311 Zm00001d011159 Zm00001d049292 Zm00001d036533 Zm00001d011901 Zm00001d046190 Zm00001d038594 Zm00001d029918 Zm00001d013694 Zm00001d025848 Zm00001d039839 Zm00001d006760 Zm00001d045538 Zm00001d051247 Zm00001d003492 Zm00001d003851 Zm00001d026425 Zm00001d023721 Zm00001d022148 Zm00001d038066 Zm00001d027758 Zm00001d034001 Zm00001d038207 Zm00001d016091 Zm00001d033775 Zm00001d038762 Zm00001d022050 Zm00001d037150 Zm00001d048487 Zm00001d005424 Zm00001d045155 Zm00001d034991 Zm00001d032546 Zm00001d052489 Zm00001d029601 Zm00001d051447 Zm00001d026702 Zm00001d042450 Zm00001d034788 Zm00001d007378 Zm00001d038519 Zm00001d051008 Zm00001d045171 Zm00001d036950 Zm00001d033931 Zm00001d053003 Zm00001d002225 Zm00001d050277 Zm00001d022038 Zm00001d040541 Zm00001d037236 Zm00001d048359 Zm00001d001855 Zm00001d053759 Zm00001d050123 Zm00001d046127 Zm00001d042481 Zm00001d023316 Zm00001d038709 Zm00001d026258 Zm00001d034124 Zm00001d031059 Zm00001d026670 Zm00001d030579 Zm00001d025397 Zm00001d041070 Zm00001d045134 Zm00001d032476 Zm00001d002441 Zm00001d012061 Zm00001d001856 Zm00001d013765 Zm00001d021938 Zm00001d028368 Zm00001d033256 Zm00001d010927 Zm00001d023880 Zm00001d033204 Zm00001d007113 Zm00001d053244 Zm00001d032681 Zm00001d012851 Zm00001d039245 Zm00001d041353 Zm00001d018142 Zm00001d034872 Zm00001d038792 Zm00001d042337 Zm00001d011252 Zm00001d012866 Zm00001d007764 Zm00001d009020 Zm00001d043845 Zm00001d019207 Zm00001d005308 Zm00001d023887 Zm00001d019184 Zm00001d019490 Zm00001d031956 Zm00001d050404 Zm00001d024305 Zm00001d010023 Zm00001d005860 Zm00001d006906 Zm00001d044312 Zm00001d038733 Zm00001d032346 Zm00001d002208 Zm00001d023330 Zm00001d036338 Zm00001d010901 Zm00001d013247 Zm00001d007965 Zm00001d019875 Zm00001d044970 Zm00001d037159 Zm00001d025060 Zm00001d028260 Zm00001d034730 Zm00001d011411 Zm00001d007075 Zm00001d008330 Zm00001d018687 Zm00001d042585 Zm00001d003103 Zm00001d024919 Zm00001d027837 Zm00001d052233 Zm00001d019123 Zm00001d048140 Zm00001d023366 Zm00001d036331 Zm00001d013503 Zm00001d011282 Zm00001d020479 Zm00001d027811 Zm00001d036796 Zm00001d014912 Zm00001d032530 Zm00001d046135 Zm00001d044289 Zm00001d013272 Zm00001d046624 Zm00001d044164 Zm00001d002836 Zm00001d003616 Zm00001d036964 Zm00001d011243 Zm00001d043192 Zm00001d053931 Zm00001d051948 Zm00001d052259 Zm00001d018082 Zm00001d010773 Zm00001d013486 Zm00001d043945 Zm00001d014065 Zm00001d049553 Zm00001d009609 Zm00001d002898 Zm00001d025021 Zm00001d037748 Zm00001d017397 Zm00001d006466 Zm00001d017640 Zm00001d016949 Zm00001d046411 Zm00001d045606 Zm00001d014112 Zm00001d042706 Zm00001d038885 Zm00001d029794 Zm00001d040947 Zm00001d009866 Zm00001d023755 Zm00001d018842 Zm00001d024109 Zm00001d009233 Zm00001d012351 Zm00001d004933 Zm00001d045479 Zm00001d016060 Zm00001d007339 Zm00001d036520 Zm00001d004960 Zm00001d022265 Zm00001d012998 Zm00001d017037 Zm00001d032139 Zm00001d032127 Zm00001d023713 Zm00001d028868 Zm00001d037582 Zm00001d012881 Zm00001d043595 Zm00001d048105 Zm00001d024624 Zm00001d013786 Zm00001d021614 Zm00001d026394 Zm00001d029552 Zm00001d003147 Zm00001d017651 Zm00001d029048 Zm00001d023452 Zm00001d027879 Zm00001d049053 Zm00001d018529 Zm00001d024384 Zm00001d029041 Zm00001d026237 Zm00001d026346 Zm00001d005199 Zm00001d007940 Zm00001d005185 Zm00001d029979 Zm00001d018983 Zm00001d016349 Zm00001d023277 Zm00001d017521 Zm00001d044693 Zm00001d005772 Zm00001d028612 Zm00001d028909 Zm00001d022582 Zm00001d048998 Zm00001d038998 Zm00001d013935 Zm00001d034064 Zm00001d002629 Zm00001d038109 Zm00001d045358 Zm00001d046249 Zm00001d045556 Zm00001d004025 Zm00001d050056 Zm00001d024933 Zm00001d041746 Zm00001d012749 Zm00001d045113 Zm00001d019856 Zm00001d023995 Zm00001d015123 Zm00001d016491 Zm00001d024408 Zm00001d053172 Zm00001d031988 Zm00001d031648 Zm00001d037640 Zm00001d033088 Zm00001d013549 Zm00001d031522 Zm00001d029910 Zm00001d049193 Zm00001d022393 Zm00001d020879 Zm00001d018195 Zm00001d054051 Zm00001d027436 Zm00001d025653 Zm00001d054031 Zm00001d037051 Zm00001d013590 Zm00001d031668 Zm00001d047092 Zm00001d028731 Zm00001d033055 Zm00001d015290 Zm00001d046864 Zm00001d010082 Zm00001d010589 Zm00001d024205 Zm00001d023910 Zm00001d024661 Zm00001d025651 Zm00001d038862 Zm00001d053983 Zm00001d025460 Zm00001d051928 Zm00001d032710 Zm00001d018990 Zm00001d034090 Zm00001d024325 Zm00001d036442 Zm00001d043303 Zm00001d005461 Zm00001d041884 Zm00001d031019 Zm00001d028952 Zm00001d036432 Zm00001d038763 Zm00001d037606 Zm00001d036656 Zm00001d039029 Zm00001d005489 Zm00001d013336 Zm00001d021962 Zm00001d011477 Zm00001d013094 Zm00001d037719 Zm00001d002531 Zm00001d013179 Zm00001d016234 Zm00001d010221 Zm00001d047309 Zm00001d016002 Zm00001d017264 Zm00001d054005 Zm00001d031641 Zm00001d015772 Zm00001d032934 Zm00001d011563 Zm00001d020110 Zm00001d035702 Zm00001d026260 Zm00001d049409 Zm00001d049928 Zm00001d029581 Zm00001d053017 Zm00001d042801 Zm00001d003128 Zm00001d052145 Zm00001d041186 Zm00001d032299 Zm00001d031120 Zm00001d026244 Zm00001d047833 Zm00001d007288 Zm00001d008422 Zm00001d002352 Zm00001d041236 Zm00001d029519 Zm00001d029419 Zm00001d014704 Zm00001d032060 Zm00001d024418 Zm00001d036946 Zm00001d024594 Zm00001d002934 Zm00001d043474 Zm00001d030500 Zm00001d008849 Zm00001d034358 Zm00001d007785 Zm00001d021495 Zm00001d038138 Zm00001d035925 Zm00001d043492 Zm00001d008529 Zm00001d006108 Zm00001d005754 Zm00001d043948 Zm00001d022302 Zm00001d029196 Zm00001d051306 Zm00001d005520 Zm00001d041789 Zm00001d031473 Zm00001d045755 Zm00001d047183 Zm00001d051252 Zm00001d015121 Zm00001d009727 Zm00001d023797 Zm00001d002854 Zm00001d039534 Zm00001d011827 Zm00001d005105 Zm00001d044692 Zm00001d042263 Zm00001d003801 Zm00001d013432 Zm00001d038529 Zm00001d025584 Zm00001d008932 Zm00001d022206 Zm00001d029375 Zm00001d013239 Zm00001d034866 Zm00001d037058 Zm00001d042686 Zm00001d045136 Zm00001d052756 Zm00001d021668 Zm00001d008417 Zm00001d052425 Zm00001d038485 Zm00001d012277 Zm00001d049360 Zm00001d028370 Zm00001d051546 Zm00001d037783 Zm00001d031706 Zm00001d009432 Zm00001d005503 Zm00001d009795 Zm00001d023659 Zm00001d013389 Zm00001d052306 Zm00001d039214 Zm00001d006836 Zm00001d039613 Zm00001d011353 Zm00001d006368 Zm00001d005630 Zm00001d039407 Zm00001d044320 Zm00001d051664 Zm00001d032992 Zm00001d047128 Zm00001d043356 Zm00001d051258 Zm00001d028686 Zm00001d016036 Zm00001d021830 Zm00001d014665 Zm00001d028662 Zm00001d030902 Zm00001d013230 Zm00001d037199 Zm00001d009440 Zm00001d011135 Zm00001d028279 Zm00001d049638 Zm00001d020516 Zm00001d038718 Zm00001d017113 Zm00001d027447 Zm00001d052314 Zm00001d025865 Zm00001d028354 Zm00001d007954 Zm00001d048819 Zm00001d005909 Zm00001d049698 Zm00001d021763 Zm00001d001932 Zm00001d036878 Zm00001d053611 Zm00001d034161 Zm00001d051325 Zm00001d048117 Zm00001d022132 Zm00001d042193 Zm00001d021072 Zm00001d006644 Zm00001d034674 Zm00001d001939 Zm00001d016077 Zm00001d037164 Zm00001d047728 Zm00001d035303 Zm00001d032608 Zm00001d025452 Zm00001d007870 Zm00001d012042 Zm00001d025144 Zm00001d020549 Zm00001d002853 Zm00001d051557 Zm00001d011308 Zm00001d033753 Zm00001d033419 Zm00001d029218 Zm00001d003655 Zm00001d017785 Zm00001d005657 Zm00001d007750 Zm00001d023465 Zm00001d011301 Zm00001d001913 Zm00001d052678 Zm00001d003091 Zm00001d033995 Zm00001d050992 Zm00001d030732 Zm00001d044921 Zm00001d003983 Zm00001d009139 Zm00001d002639 Zm00001d028438 Zm00001d039620 Zm00001d043076 Zm00001d026154 Zm00001d050319 Zm00001d004992 Zm00001d012962 Zm00001d003949 Zm00001d010056 Zm00001d053372 Zm00001d038892 Zm00001d007479 Zm00001d004877 Zm00001d020180 Zm00001d018440 Zm00001d009425 Zm00001d017120 Zm00001d002478 Zm00001d034351 Zm00001d013653 Zm00001d049715 Zm00001d013075 Zm00001d038432 Zm00001d006308 Zm00001d036895 Zm00001d019114 Zm00001d028733 Zm00001d036880 Zm00001d007858 Zm00001d034454 Zm00001d007354 Zm00001d030790 Zm00001d024704 Zm00001d044759 Zm00001d044601 Zm00001d053686 Zm00001d034498 Zm00001d019573 Zm00001d001901 Zm00001d007972 Zm00001d020834 Zm00001d007153 Zm00001d041397 Zm00001d015657 Zm00001d028705 Zm00001d020640 Zm00001d014987 Zm00001d008412 Zm00001d050051 Zm00001d013218 Zm00001d043895 Zm00001d043074 Zm00001d038739 Zm00001d053977 Zm00001d026517 Zm00001d011288 Zm00001d048180 Zm00001d039118 Zm00001d018944 Zm00001d042661 Zm00001d017193 Zm00001d047823 Zm00001d045435 Zm00001d047689 Zm00001d004771 Zm00001d032099 Zm00001d044781 Zm00001d020706 Zm00001d050935 Zm00001d038988 Zm00001d042916 Zm00001d005347 Zm00001d039518 Zm00001d054014 Zm00001d048461 Zm00001d021379 Zm00001d011091 Zm00001d016180 Zm00001d025456 Zm00001d023885 Zm00001d054000 Zm00001d028773 Zm00001d001884 Zm00001d018513 Zm00001d025663 Zm00001d046939 Zm00001d014998 Zm00001d017165 Zm00001d027514 Zm00001d033600 Zm00001d017106 Zm00001d043352 Zm00001d034698 Zm00001d042432 Zm00001d022333 Zm00001d009910 Zm00001d035598 Zm00001d014306 Zm00001d009320 Zm00001d044216 Zm00001d003352 Zm00001d010305 Zm00001d011830 Zm00001d018616 Zm00001d029768 Zm00001d011821 Zm00001d040757 Zm00001d017014 Zm00001d051528 Zm00001d026158 Zm00001d035065 Zm00001d018598 Zm00001d053171 Zm00001d052977 Zm00001d004737 Zm00001d039641 Zm00001d043125 Zm00001d052530 Zm00001d047831 Zm00001d028244 Zm00001d041590 Zm00001d043850 Zm00001d043843 Zm00001d044315 Zm00001d028360 Zm00001d017401 Zm00001d004159 Zm00001d005925 Zm00001d041365 Zm00001d037289 Zm00001d026326 Zm00001d019568 Zm00001d051746 Zm00001d014135 Zm00001d004023 Zm00001d050696 Zm00001d002357 Zm00001d036013 Zm00001d017258 Zm00001d012283 Zm00001d011370 Zm00001d014523 Zm00001d022395 Zm00001d005065 Zm00001d039496 Zm00001d051879 Zm00001d052067 Zm00001d049625 Zm00001d007092 Zm00001d018906 Zm00001d005178 Zm00001d044685 Zm00001d038541 Zm00001d044502 Zm00001d010653 Zm00001d038143 Zm00001d013406 Zm00001d009359 Zm00001d034069 Zm00001d020971 Zm00001d042792 Zm00001d026368 Zm00001d014340 Zm00001d042935 Zm00001d050048 Zm00001d011758 Zm00001d024258 Zm00001d013908 Zm00001d020936 Zm00001d009512 Zm00001d016604 Zm00001d022190 Zm00001d016731 Zm00001d048007 Zm00001d047555 Zm00001d028241 Zm00001d051946 Zm00001d021982 Zm00001d009763 Zm00001d005547 Zm00001d035029 Zm00001d019165 Zm00001d039325 Zm00001d033895 Zm00001d032667 Zm00001d042985 Zm00001d013560 Zm00001d020311 Zm00001d042140 Zm00001d023445 Zm00001d022440 Zm00001d053748 Zm00001d019039 Zm00001d010944 Zm00001d020673 Zm00001d031426 Zm00001d036020 Zm00001d003477 Zm00001d002428 Zm00001d014861 Zm00001d015556 Zm00001d028258 Zm00001d038684 Zm00001d021580 Zm00001d026639 Zm00001d033557 Zm00001d027861 Zm00001d002373 Zm00001d028675 Zm00001d051015 Zm00001d029548 Zm00001d053453 Zm00001d011676 Zm00001d039138 Zm00001d005846 Zm00001d011242 Zm00001d024248 Zm00001d012839 Zm00001d002682 Zm00001d013060 Zm00001d002842 Zm00001d021554 Zm00001d018277 Zm00001d022589 Zm00001d024765 Zm00001d001953 Zm00001d026335 Zm00001d039111 Zm00001d028213 Zm00001d030108 Zm00001d020152 Zm00001d033262 Zm00001d050726 Zm00001d037420 Zm00001d006430 Zm00001d028273 Zm00001d013664 Zm00001d047804 Zm00001d024053 Zm00001d035593 Zm00001d012098 Zm00001d032467 Zm00001d021410 Zm00001d043288 Zm00001d051138 Zm00001d021570 Zm00001d012801 Zm00001d042487 Zm00001d037214 Zm00001d004448 Zm00001d015612 Zm00001d053676 Zm00001d037728 Zm00001d011577 Zm00001d028008 Zm00001d028443 Zm00001d007044 Zm00001d038536 Zm00001d009783 Zm00001d047843 Zm00001d042088 Zm00001d047443 Zm00001d048355 Zm00001d005851 Zm00001d043813 Zm00001d043827 Zm00001d040038 Zm00001d013891 Zm00001d021715 Zm00001d050294 Zm00001d041741 Zm00001d016512 Zm00001d014203 Zm00001d048686 Zm00001d002029 Zm00001d043468 Zm00001d045389 Zm00001d019426 Zm00001d037593 Zm00001d027699 Zm00001d020807 Zm00001d002553 Zm00001d044831 Zm00001d039769 Zm00001d009410 Zm00001d012144 Zm00001d023968 Zm00001d039904 Zm00001d045757 Zm00001d035170 Zm00001d046214 Zm00001d025817 Zm00001d006517 Zm00001d035098 Zm00001d042996 Zm00001d006504 Zm00001d052595 Zm00001d053952 Zm00001d031587 Zm00001d003824 Zm00001d034076 Zm00001d039271 Zm00001d049505 Zm00001d025279 Zm00001d031060 Zm00001d022490 Zm00001d002095 Zm00001d039278 Zm00001d012030 Zm00001d041951 Zm00001d023729 Zm00001d042974 Zm00001d026562 Zm00001d013611 Zm00001d005193 Zm00001d006347 Zm00001d028109 Zm00001d047683 Zm00001d053981 Zm00001d023836 Zm00001d007180 Zm00001d027803 Zm00001d047977 Zm00001d024938 Zm00001d025210 Zm00001d042269 Zm00001d003313 Zm00001d003923 Zm00001d008256 Zm00001d016928 Zm00001d019684 Zm00001d005856 Zm00001d014600 Zm00001d006079 Zm00001d008870 Zm00001d029983 Zm00001d012571 Zm00001d036654 Zm00001d005958 Zm00001d050635 Zm00001d029487 Zm00001d033591 Zm00001d006421 Zm00001d018464 Zm00001d015226 Zm00001d028415 Zm00001d039471 Zm00001d010644 Zm00001d002243 Zm00001d016008 Zm00001d043962 Zm00001d047426 Zm00001d013193 Zm00001d053541 Zm00001d024796 Zm00001d011087 Zm00001d036280 Zm00001d013979 Zm00001d002864 Zm00001d051475 Zm00001d003640 Zm00001d020292 Zm00001d038217 Zm00001d010632 Zm00001d031481 Zm00001d037626 Zm00001d006586 Zm00001d047898 Zm00001d028782 Zm00001d027603 Zm00001d006795 Zm00001d049126 Zm00001d027524 Zm00001d027519 Zm00001d015945 Zm00001d024816 Zm00001d029184 Zm00001d053396 Zm00001d037005 Zm00001d051690 Zm00001d031784 Zm00001d016595 Zm00001d039311 Zm00001d034373 Zm00001d013380 Zm00001d004616 Zm00001d033675 Zm00001d007807 Zm00001d010314 Zm00001d046001 Zm00001d032078 Zm00001d049540 Zm00001d052312 Zm00001d017787 Zm00001d032331 Zm00001d022142 Zm00001d028532 Zm00001d029754 Zm00001d046342 Zm00001d020365 Zm00001d008458 Zm00001d049155 Zm00001d048432 Zm00001d027427 Zm00001d041926 Zm00001d049387 Zm00001d013073 Zm00001d003257 Zm00001d024518 Zm00001d016052 Zm00001d021592 Zm00001d048836 Zm00001d035194 Zm00001d034669 Zm00001d010054 Zm00001d021287 Zm00001d023329 Zm00001d005716 Zm00001d047484 Zm00001d042694 Zm00001d021490 Zm00001d046112 Zm00001d026293 Zm00001d053627 Zm00001d038850 Zm00001d043096 Zm00001d025322 Zm00001d045673 Zm00001d051599 Zm00001d034721 Zm00001d047273 Zm00001d039608 Zm00001d038407 Zm00001d040124 Zm00001d020617 Zm00001d030173 Zm00001d012770 Zm00001d024027 Zm00001d052874 Zm00001d032030 Zm00001d014382 Zm00001d041873 Zm00001d016103 Zm00001d016009 Zm00001d031817 Zm00001d018454 Zm00001d029647 Zm00001d047813 Zm00001d042589 Zm00001d015037 Zm00001d006876 Zm00001d038917 Zm00001d023682 Zm00001d038600 Zm00001d035700 Zm00001d016835 Zm00001d036966 Zm00001d041550 Zm00001d025837 Zm00001d043328 Zm00001d005726 Zm00001d011137 Zm00001d046672 Zm00001d032166 Zm00001d024738 Zm00001d034590 Zm00001d017448 Zm00001d008567 Zm00001d024416 Zm00001d038380 Zm00001d032009 Zm00001d008721 Zm00001d049583 Zm00001d044556 Zm00001d017567 Zm00001d042632 Zm00001d040678 Zm00001d030087 Zm00001d011565 Zm00001d005636 Zm00001d021788 Zm00001d032873 Zm00001d052153 Zm00001d011139 Zm00001d038367 Zm00001d052061 Zm00001d052056 Zm00001d012662 Zm00001d043752 Zm00001d037323 Zm00001d039460 Zm00001d049687 Zm00001d011130 Zm00001d009074 Zm00001d013812 Zm00001d044874 Zm00001d008777 Zm00001d052303 Zm00001d045571 Zm00001d017771 Zm00001d031805 Zm00001d001827 Zm00001d052997 Zm00001d008726 Zm00001d009189 Zm00001d050816 Zm00001d014898 Zm00001d037610 Zm00001d047635 Zm00001d021491 Zm00001d024824 Zm00001d012469 Zm00001d012924 Zm00001d040365 Zm00001d024048 Zm00001d016621 Zm00001d050755 Zm00001d012224 Zm00001d011581 Zm00001d050728 Zm00001d013296 Zm00001d044332 Zm00001d020185 Zm00001d012687 Zm00001d009856 Zm00001d049627 Zm00001d014385 Zm00001d010455 Zm00001d009707 Zm00001d034638 Zm00001d010556 Zm00001d045696 Zm00001d034862 Zm00001d035723 Zm00001d007405 Zm00001d039300 Zm00001d046334 Zm00001d032010 Zm00001d038480 Zm00001d038330 Zm00001d024729 Zm00001d048721 Zm00001d018443 Zm00001d027239 Zm00001d025050 Zm00001d008749 Zm00001d031251 Zm00001d011642 Zm00001d018439 Zm00001d022107 Zm00001d012244 Zm00001d028113 Zm00001d024702 Zm00001d017296 Zm00001d036829 Zm00001d002822 Zm00001d052930 Zm00001d015932 Zm00001d007960 Zm00001d018200 Zm00001d038359 Zm00001d006630 Zm00001d052074 Zm00001d041595 Zm00001d035254 Zm00001d047536 Zm00001d016602 Zm00001d040721 Zm00001d018668 Zm00001d017345 Zm00001d006936 Zm00001d043986 Zm00001d036262 Zm00001d018939 Zm00001d044586 Zm00001d034586 Zm00001d045723 Zm00001d034457 Zm00001d017239 Zm00001d051650 Zm00001d023934 Zm00001d033623 Zm00001d026585 Zm00001d005028 Zm00001d026584 Zm00001d013456 Zm00001d033153 Zm00001d042084 Zm00001d012529 Zm00001d011298 Zm00001d046718 Zm00001d028047 Zm00001d003935 Zm00001d009723 Zm00001d002090 Zm00001d007318 Zm00001d019233 Zm00001d024511 Zm00001d004740 Zm00001d026590 Zm00001d037305 Zm00001d048703 Zm00001d002503 Zm00001d026302 Zm00001d037114 Zm00001d015893 Zm00001d049493 Zm00001d042469 Zm00001d041511 Zm00001d027949 Zm00001d011706 Zm00001d006834 Zm00001d051911 Zm00001d050320 Zm00001d044184 Zm00001d039449 Zm00001d048335 Zm00001d040696 Zm00001d041580 Zm00001d002282 Zm00001d035669 Zm00001d028203 Zm00001d011086 Zm00001d026266 Zm00001d002246 Zm00001d028720 Zm00001d012955 Zm00001d053747 Zm00001d032293 Zm00001d028229 Zm00001d035230 Zm00001d038204 Zm00001d038387 Zm00001d021825 Zm00001d047150 Zm00001d003493 Zm00001d013176 Zm00001d013547 Zm00001d044457 Zm00001d049024 Zm00001d043511 Zm00001d036940 Zm00001d047053 Zm00001d018895 Zm00001d033610 Zm00001d051268 Zm00001d034512 Zm00001d041854 Zm00001d012286 Zm00001d043151 Zm00001d008424 Zm00001d025681 Zm00001d034680 Zm00001d025767 Zm00001d012689 Zm00001d049293 Zm00001d030968 Zm00001d046900 Zm00001d033948 Zm00001d016402 Zm00001d021201 Zm00001d015627 Zm00001d005269 Zm00001d045632 Zm00001d038538 Zm00001d039456 Zm00001d048625 Zm00001d006702 Zm00001d001798 Zm00001d053776 Zm00001d049746 Zm00001d052589 Zm00001d006124 Zm00001d021710 Zm00001d017846 Zm00001d028064 Zm00001d033028 Zm00001d038165 Zm00001d007043 Zm00001d023258 Zm00001d017721 Zm00001d018086 Zm00001d049222 Zm00001d003607 Zm00001d038337 Zm00001d018669 Zm00001d026669 Zm00001d015952 Zm00001d027919 Zm00001d034606 Zm00001d024008 Zm00001d042284 Zm00001d045181 Zm00001d034104 Zm00001d013162 Zm00001d051884 Zm00001d009503 Zm00001d021613 Zm00001d028588 Zm00001d024425 Zm00001d017811 Zm00001d041759 Zm00001d005368 Zm00001d039403 Zm00001d049383 Zm00001d017772 Zm00001d018286 Zm00001d018718 Zm00001d024322 Zm00001d014226 Zm00001d049737 Zm00001d024245 Zm00001d049082 Zm00001d041852 Zm00001d042146 Zm00001d011286 Zm00001d011373 Zm00001d009813 Zm00001d031209 Zm00001d015148 Zm00001d048102 Zm00001d002433 Zm00001d028026 Zm00001d026353 Zm00001d039013 Zm00001d049332 Zm00001d031941 Zm00001d033488 Zm00001d024339 Zm00001d045133 Zm00001d049797 Zm00001d042965 Zm00001d051812 Zm00001d025027 Zm00001d020112 Zm00001d003504 Zm00001d054013 Zm00001d009016 Zm00001d032687 Zm00001d046890 Zm00001d003774 Zm00001d038121 Zm00001d045183 Zm00001d030936 Zm00001d021347 Zm00001d029200 Zm00001d049095 Zm00001d012513 Zm00001d046397 Zm00001d025131 Zm00001d051733 Zm00001d034716 Zm00001d038691 Zm00001d003393 Zm00001d048282 Zm00001d028211 Zm00001d027590 Zm00001d038027 Zm00001d008502 Zm00001d046166 Zm00001d002806 Zm00001d038955 Zm00001d025258 Zm00001d003259 Zm00001d044866 Zm00001d014345 Zm00001d013746 Zm00001d048839 Zm00001d030126 Zm00001d052423 Zm00001d017765 Zm00001d032679 Zm00001d045323 Zm00001d006317 Zm00001d031072 Zm00001d012395 Zm00001d046444 Zm00001d023469 Zm00001d052744 Zm00001d050811 Zm00001d047531 Zm00001d025656 Zm00001d034670 Zm00001d052769 Zm00001d043941 Zm00001d038171 Zm00001d014123 Zm00001d034883 Zm00001d017836 Zm00001d040061 Zm00001d002756 Zm00001d046919 Zm00001d013228 Zm00001d043879 Zm00001d025916 Zm00001d016911 Zm00001d012380 Zm00001d007294 Zm00001d023998 Zm00001d053066 Zm00001d034817 Zm00001d019981 Zm00001d044701 Zm00001d042367 Zm00001d032472 Zm00001d041769 Zm00001d005894 Zm00001d032604 Zm00001d028218 Zm00001d029810 Zm00001d005849 Zm00001d035625 Zm00001d011225 Zm00001d009137 Zm00001d008716 Zm00001d020052 Zm00001d023838 Zm00001d021487 Zm00001d030518 Zm00001d046377 Zm00001d021629 Zm00001d038267 Zm00001d033882 Zm00001d005159 Zm00001d011766 Zm00001d007095 Zm00001d016276 Zm00001d028726 Zm00001d006534 Zm00001d018504 Zm00001d022322 Zm00001d046790 Zm00001d038134 Zm00001d053232 Zm00001d010066 Zm00001d026278 Zm00001d045497 Zm00001d014489 Zm00001d037663 Zm00001d006355 Zm00001d029189 Zm00001d028232 Zm00001d023291 Zm00001d009404 Zm00001d013971 Zm00001d009771 Zm00001d050618 Zm00001d006602 Zm00001d037124 Zm00001d015208 Zm00001d049982 Zm00001d017416 Zm00001d017139 Zm00001d017959 Zm00001d034662 Zm00001d002537 Zm00001d002891 Zm00001d043198 Zm00001d033917 Zm00001d030061 Zm00001d037512 Zm00001d012653 Zm00001d036396 Zm00001d034179 Zm00001d051135 Zm00001d018522 Zm00001d002137 Zm00001d053090 Zm00001d040734 Zm00001d029611 Zm00001d041222 Zm00001d027897 Zm00001d050107 Zm00001d029597 Zm00001d007692 Zm00001d002579 Zm00001d030916 Zm00001d020870 Zm00001d017868 Zm00001d014895 Zm00001d026285 Zm00001d011705 Zm00001d010690 Zm00001d017925 Zm00001d048516 Zm00001d038292 Zm00001d033321 Zm00001d046602 Zm00001d019282 Zm00001d049379 Zm00001d053616 Zm00001d019054 Zm00001d035695 Zm00001d015426 Zm00001d044783 Zm00001d021132 Zm00001d003179 Zm00001d048635 Zm00001d034956 Zm00001d037735 Zm00001d053552 Zm00001d029416 Zm00001d012849 Zm00001d045397 Zm00001d033214 Zm00001 |
| Beijing | 693 | Zm00001d019163 Zm00001d037918 Zm00001d015139 Zm00001d049831 Zm00001d034356 Zm00001d013573 Zm00001d005168 Zm00001d041418 Zm00001d052194 Zm00001d025831 Zm00001d052037 Zm00001d042350 Zm00001d027441 Zm00001d043923 Zm00001d021971 Zm00001d029822 Zm00001d047801 Zm00001d028295 Zm00001d024687 Zm00001d045106 Zm00001d029214 Zm00001d018335 Zm00001d048169 Zm00001d033766 Zm00001d036738 Zm00001d021014 Zm00001d038364 Zm00001d013824 Zm00001d035125 Zm00001d039854 Zm00001d043386 Zm00001d039582 Zm00001d030796 Zm00001d021935 Zm00001d044159 Zm00001d002261 Zm00001d008731 Zm00001d029628 Zm00001d034248 Zm00001d007773 Zm00001d012994 Zm00001d017186 Zm00001d029974 Zm00001d012855 Zm00001d052375 Zm00001d043993 Zm00001d004751 Zm00001d047603 Zm00001d025037 Zm00001d019200 Zm00001d006166 Zm00001d046587 Zm00001d014486 Zm00001d008462 Zm00001d001973 Zm00001d016803 Zm00001d015744 Zm00001d041988 Zm00001d027742 Zm00001d035750 Zm00001d028691 Zm00001d030412 Zm00001d050705 Zm00001d042508 Zm00001d007076 Zm00001d004259 Zm00001d015406 Zm00001d023908 Zm00001d052928 Zm00001d012795 Zm00001d038896 Zm00001d044495 Zm00001d039321 Zm00001d036359 Zm00001d012725 Zm00001d042018 Zm00001d049725 Zm00001d047794 Zm00001d042627 Zm00001d040818 Zm00001d013644 Zm00001d035400 Zm00001d013122 Zm00001d018261 Zm00001d027505 Zm00001d048342 Zm00001d032204 Zm00001d003993 Zm00001d021607 Zm00001d050550 Zm00001d016801 Zm00001d026135 Zm00001d024164 Zm00001d031267 Zm00001d025659 Zm00001d045394 Zm00001d038992 Zm00001d043339 Zm00001d026619 Zm00001d018554 Zm00001d040639 Zm00001d006340 Zm00001d029457 Zm00001d051865 Zm00001d045026 Zm00001d009116 Zm00001d002172 Zm00001d042640 Zm00001d033551 Zm00001d014471 Zm00001d049161 Zm00001d004342 Zm00001d012167 Zm00001d013098 Zm00001d038622 Zm00001d006533 Zm00001d050106 Zm00001d012627 Zm00001d038318 Zm00001d048204 Zm00001d035094 Zm00001d001880 Zm00001d042636 Zm00001d039821 Zm00001d029750 Zm00001d039276 Zm00001d022326 Zm00001d039106 Zm00001d018665 Zm00001d012969 Zm00001d045897 Zm00001d028761 Zm00001d013858 Zm00001d004348 Zm00001d006899 Zm00001d018490 Zm00001d001899 Zm00001d031423 Zm00001d039439 Zm00001d039746 Zm00001d031502 Zm00001d022164 Zm00001d008916 Zm00001d028820 Zm00001d015985 Zm00001d032199 Zm00001d044024 Zm00001d008874 Zm00001d027719 Zm00001d037070 Zm00001d017724 Zm00001d045340 Zm00001d032588 Zm00001d006377 Zm00001d012681 Zm00001d048417 Zm00001d048702 Zm00001d031290 Zm00001d011002 Zm00001d017653 Zm00001d040455 Zm00001d038650 Zm00001d045495 Zm00001d040067 Zm00001d034941 Zm00001d010400 Zm00001d007581 Zm00001d001785 Zm00001d037105 Zm00001d031824 Zm00001d050501 Zm00001d042738 Zm00001d003064 Zm00001d035815 Zm00001d047806 Zm00001d046697 Zm00001d032206 Zm00001d006270 Zm00001d018370 Zm00001d013023 Zm00001d009652 Zm00001d026300 Zm00001d029473 Zm00001d020496 Zm00001d006502 Zm00001d053963 Zm00001d014947 Zm00001d005200 Zm00001d001811 Zm00001d013271 Zm00001d033992 Zm00001d014778 Zm00001d003212 Zm00001d050125 Zm00001d012899 Zm00001d030509 Zm00001d016256 Zm00001d017992 Zm00001d006510 Zm00001d021608 Zm00001d039211 Zm00001d014116 Zm00001d026712 Zm00001d044036 Zm00001d053652 Zm00001d048959 Zm00001d021025 Zm00001d029761 Zm00001d010200 Zm00001d020025 Zm00001d018098 Zm00001d006511 Zm00001d051156 Zm00001d014858 Zm00001d024567 Zm00001d026224 Zm00001d030727 Zm00001d038859 Zm00001d052435 Zm00001d011183 Zm00001d008611 Zm00001d014553 Zm00001d002285 Zm00001d032396 Zm00001d040619 Zm00001d027532 Zm00001d008173 Zm00001d038768 Zm00001d043361 Zm00001d032812 Zm00001d038227 Zm00001d051974 Zm00001d013725 Zm00001d003268 Zm00001d045298 Zm00001d022367 Zm00001d001895 Zm00001d050234 Zm00001d048908 Zm00001d013325 Zm00001d021745 Zm00001d037916 Zm00001d010428 Zm00001d009975 Zm00001d050266 Zm00001d041583 Zm00001d003294 Zm00001d049389 Zm00001d017703 Zm00001d015570 Zm00001d014580 Zm00001d003287 Zm00001d050222 Zm00001d006331 Zm00001d014769 Zm00001d042850 Zm00001d019454 Zm00001d008294 Zm00001d017209 Zm00001d017621 Zm00001d027478 Zm00001d021978 Zm00001d044669 Zm00001d026633 Zm00001d046586 Zm00001d004553 Zm00001d002650 Zm00001d011799 Zm00001d038167 Zm00001d010672 Zm00001d048876 Zm00001d027839 Zm00001d004931 Zm00001d039687 Zm00001d003830 Zm00001d018803 Zm00001d035319 Zm00001d012137 Zm00001d003395 Zm00001d037533 Zm00001d011644 Zm00001d029272 Zm00001d011275 Zm00001d012938 Zm00001d027677 Zm00001d024155 Zm00001d030032 Zm00001d011532 Zm00001d032155 Zm00001d003067 Zm00001d039786 Zm00001d029289 Zm00001d005421 Zm00001d009990 Zm00001d035222 Zm00001d022384 Zm00001d003297 Zm00001d041191 Zm00001d032621 Zm00001d011736 Zm00001d021572 Zm00001d037315 Zm00001d041346 Zm00001d011708 Zm00001d047944 Zm00001d044253 Zm00001d048505 Zm00001d034043 Zm00001d002848 Zm00001d022154 Zm00001d050341 Zm00001d050837 Zm00001d045296 Zm00001d008297 Zm00001d048134 Zm00001d015231 Zm00001d042490 Zm00001d010535 Zm00001d048787 Zm00001d049945 Zm00001d048833 Zm00001d003660 Zm00001d035820 Zm00001d050963 Zm00001d003112 Zm00001d049858 Zm00001d053707 Zm00001d020793 Zm00001d004078 Zm00001d031790 Zm00001d038198 Zm00001d014581 Zm00001d043031 Zm00001d017788 Zm00001d013443 Zm00001d007070 Zm00001d018937 Zm00001d033951 Zm00001d007889 Zm00001d052170 Zm00001d028071 Zm00001d044291 Zm00001d018472 Zm00001d036097 Zm00001d048503 Zm00001d046937 Zm00001d007012 Zm00001d003019 Zm00001d003174 Zm00001d009085 Zm00001d022386 Zm00001d004898 Zm00001d033634 Zm00001d043364 Zm00001d012693 Zm00001d038365 Zm00001d037648 Zm00001d040527 Zm00001d013005 Zm00001d033719 Zm00001d006776 Zm00001d016895 Zm00001d025765 Zm00001d032190 Zm00001d048474 Zm00001d044564 Zm00001d021991 Zm00001d052792 Zm00001d019164 Zm00001d002679 Zm00001d044109 Zm00001d007793 Zm00001d039026 Zm00001d040040 Zm00001d043206 Zm00001d010614 Zm00001d045393 Zm00001d019994 Zm00001d006758 Zm00001d036255 Zm00001d011496 Zm00001d038051 Zm00001d026192 Zm00001d054105 Zm00001d007106 Zm00001d037929 Zm00001d008984 Zm00001d048137 Zm00001d005504 Zm00001d052972 Zm00001d047090 Zm00001d006496 Zm00001d007836 Zm00001d034782 Zm00001d051339 Zm00001d041530 Zm00001d043404 Zm00001d030623 Zm00001d037612 Zm00001d005594 Zm00001d006051 Zm00001d038218 Zm00001d053293 Zm00001d050874 Zm00001d022530 Zm00001d006700 Zm00001d020915 Zm00001d036875 Zm00001d007825 Zm00001d044785 Zm00001d015423 Zm00001d011958 Zm00001d033384 Zm00001d054078 Zm00001d021288 Zm00001d040254 Zm00001d024786 Zm00001d017467 Zm00001d024573 Zm00001d044641 Zm00001d021875 Zm00001d039987 Zm00001d032295 Zm00001d046626 Zm00001d029318 Zm00001d002661 Zm00001d017412 Zm00001d026495 Zm00001d052076 Zm00001d035879 Zm00001d019724 Zm00001d053800 Zm00001d039444 Zm00001d032811 Zm00001d025228 Zm00001d032075 Zm00001d044212 Zm00001d024677 Zm00001d004152 Zm00001d052237 Zm00001d045661 Zm00001d013673 Zm00001d045074 Zm00001d012335 Zm00001d013777 Zm00001d025619 Zm00001d020017 Zm00001d018821 Zm00001d004705 Zm00001d032916 Zm00001d007207 Zm00001d042256 Zm00001d043515 Zm00001d034635 Zm00001d027440 Zm00001d024423 Zm00001d033966 Zm00001d008925 Zm00001d038753 Zm00001d021263 Zm00001d002834 Zm00001d044995 Zm00001d042274 Zm00001d003023 Zm00001d021177 Zm00001d052112 Zm00001d009000 Zm00001d029662 Zm00001d021883 Zm00001d045492 Zm00001d007016 Zm00001d041711 Zm00001d032045 Zm00001d007197 Zm00001d019291 Zm00001d022462 Zm00001d010869 Zm00001d005124 Zm00001d036100 Zm00001d008808 Zm00001d010234 Zm00001d053626 Zm00001d036177 Zm00001d009506 Zm00001d033717 Zm00001d020188 Zm00001d014193 Zm00001d049158 Zm00001d020636 Zm00001d002450 Zm00001d017558 Zm00001d043290 Zm00001d019061 Zm00001d023240 Zm00001d053639 Zm00001d054003 Zm00001d029869 Zm00001d053107 Zm00001d048222 Zm00001d014752 Zm00001d022514 Zm00001d030713 Zm00001d049613 Zm00001d048370 Zm00001d026594 Zm00001d015355 Zm00001d028363 Zm00001d002996 Zm00001d003102 Zm00001d007154 Zm00001d048731 Zm00001d029524 Zm00001d037025 Zm00001d047838 Zm00001d018032 Zm00001d052533 Zm00001d001980 Zm00001d029040 Zm00001d027613 Zm00001d048561 Zm00001d026084 Zm00001d028821 Zm00001d043903 Zm00001d044411 Zm00001d038531 Zm00001d044542 Zm00001d025887 Zm00001d028915 Zm00001d044654 Zm00001d022126 Zm00001d042683 Zm00001d008503 Zm00001d017373 Zm00001d049277 Zm00001d038535 Zm00001d053283 Zm00001d053395 Zm00001d024338 Zm00001d041568 Zm00001d032422 Zm00001d015293 Zm00001d044599 Zm00001d008307 Zm00001d008467 Zm00001d041920 Zm00001d038937 Zm00001d017706 Zm00001d042308 Zm00001d036131 Zm00001d039252 Zm00001d033457 Zm00001d038114 Zm00001d017678 Zm00001d029704 Zm00001d009509 Zm00001d045125 Zm00001d029443 Zm00001d010795 Zm00001d039895 Zm00001d025575 Zm00001d009858 Zm00001d028319 Zm00001d028661 Zm00001d038528 Zm00001d048349 Zm00001d014696 Zm00001d047495 Zm00001d005973 Zm00001d025640 Zm00001d036339 Zm00001d043563 Zm00001d023734 Zm00001d036709 Zm00001d048877 Zm00001d046157 Zm00001d007254 Zm00001d032238 Zm00001d018473 Zm00001d028774 Zm00001d021626 Zm00001d043523 Zm00001d034031 Zm00001d013010 Zm00001d008665 Zm00001d047306 Zm00001d013934 Zm00001d035124 Zm00001d012736 Zm00001d009295 Zm00001d035789 Zm00001d008535 Zm00001d034876 Zm00001d052742 Zm00001d008604 Zm00001d004477 Zm00001d009108 Zm00001d034270 Zm00001d031523 Zm00001d048284 |
| Harbin | 2952 | Zm00001d032654 Zm00001d038729 Zm00001d026352 Zm00001d031958 Zm00001d039541 Zm00001d048492 Zm00001d045302 id Zm00001d015618 Zm00001d021950 Zm00001d034040 Zm00001d043860 Zm00001d012838 Zm00001d045949 Zm00001d005816 Zm00001d044011 Zm00001d031908 Zm00001d052422 Zm00001d051806 Zm00001d005975 Zm00001d029337 Zm00001d014540 Zm00001d010413 Zm00001d027900 Zm00001d033558 Zm00001d043540 Zm00001d019117 Zm00001d031332 Zm00001d049331 Zm00001d010606 Zm00001d013507 Zm00001d044839 Zm00001d010804 Zm00001d009449 Zm00001d045984 Zm00001d020358 Zm00001d049494 Zm00001d045735 Zm00001d013794 Zm00001d043700 Zm00001d043801 Zm00001d038699 Zm00001d051917 Zm00001d016902 Zm00001d042721 Zm00001d034553 Zm00001d012267 Zm00001d005705 Zm00001d038692 Zm00001d017930 Zm00001d047532 Zm00001d024321 Zm00001d032457 Zm00001d019824 Zm00001d028776 Zm00001d005001 Zm00001d042055 Zm00001d054054 Zm00001d023235 Zm00001d046786 Zm00001d012776 Zm00001d042830 Zm00001d041168 Zm00001d010171 Zm00001d030069 Zm00001d048524 Zm00001d047442 Zm00001d008226 Zm00001d013427 Zm00001d048203 Zm00001d023923 Zm00001d042611 Zm00001d042225 Zm00001d044982 Zm00001d019560 Zm00001d014901 Zm00001d009407 Zm00001d043449 Zm00001d039435 Zm00001d021790 Zm00001d042853 Zm00001d032098 Zm00001d044144 Zm00001d039637 Zm00001d051500 Zm00001d018791 Zm00001d021390 Zm00001d030361 Zm00001d018112 Zm00001d024270 Zm00001d039046 Zm00001d023892 Zm00001d032847 Zm00001d048651 Zm00001d046690 Zm00001d008269 Zm00001d014062 Zm00001d033573 Zm00001d012787 Zm00001d025507 Zm00001d017284 Zm00001d013896 Zm00001d018648 Zm00001d053630 Zm00001d025774 Zm00001d006905 Zm00001d034729 Zm00001d012610 Zm00001d005497 Zm00001d039631 Zm00001d049138 Zm00001d050134 Zm00001d039667 Zm00001d035475 Zm00001d008209 Zm00001d009646 Zm00001d045251 Zm00001d031883 Zm00001d013150 Zm00001d043019 Zm00001d034835 Zm00001d047470 Zm00001d024522 Zm00001d018404 Zm00001d033883 Zm00001d016557 Zm00001d040144 Zm00001d030138 Zm00001d016764 Zm00001d035086 Zm00001d044566 Zm00001d052318 Zm00001d047256 Zm00001d018936 Zm00001d038540 Zm00001d008586 Zm00001d020789 Zm00001d044110 Zm00001d007473 Zm00001d018940 Zm00001d028812 Zm00001d019079 Zm00001d051900 Zm00001d026704 Zm00001d038127 Zm00001d026487 Zm00001d023376 Zm00001d037492 Zm00001d035051 Zm00001d027585 Zm00001d005324 Zm00001d030213 Zm00001d022356 Zm00001d052721 Zm00001d039273 Zm00001d021733 Zm00001d016100 Zm00001d038336 Zm00001d001779 Zm00001d010060 Zm00001d045417 Zm00001d018756 Zm00001d028055 Zm00001d052732 Zm00001d042152 Zm00001d037252 Zm00001d006103 Zm00001d033979 Zm00001d045760 Zm00001d040215 Zm00001d006506 Zm00001d047355 Zm00001d012561 Zm00001d026392 Zm00001d013821 Zm00001d026698 Zm00001d033749 Zm00001d001807 Zm00001d004914 Zm00001d018363 Zm00001d010907 Zm00001d005370 Zm00001d020670 Zm00001d022259 Zm00001d004706 Zm00001d039489 Zm00001d007205 Zm00001d038362 Zm00001d052837 Zm00001d018973 Zm00001d029555 Zm00001d042461 Zm00001d016172 Zm00001d036401 Zm00001d012381 Zm00001d016049 Zm00001d039116 Zm00001d009468 Zm00001d015491 Zm00001d037396 Zm00001d047876 Zm00001d043529 Zm00001d016185 Zm00001d022052 Zm00001d015616 Zm00001d047583 Zm00001d005392 Zm00001d017966 Zm00001d018287 Zm00001d028992 Zm00001d007026 Zm00001d042345 Zm00001d013046 Zm00001d035304 Zm00001d030640 Zm00001d039899 Zm00001d015014 Zm00001d005814 Zm00001d030953 Zm00001d026500 Zm00001d044056 Zm00001d044468 Zm00001d040313 Zm00001d034672 Zm00001d006336 Zm00001d011970 Zm00001d050018 Zm00001d008577 Zm00001d001781 Zm00001d014003 Zm00001d011483 Zm00001d039196 Zm00001d024275 Zm00001d009152 Zm00001d033166 Zm00001d029009 Zm00001d017168 Zm00001d018125 Zm00001d005632 Zm00001d044096 Zm00001d004366 Zm00001d011919 Zm00001d038133 Zm00001d035552 Zm00001d008408 Zm00001d048337 Zm00001d045927 Zm00001d039851 Zm00001d015028 Zm00001d051119 Zm00001d045313 Zm00001d016550 Zm00001d039682 Zm00001d006002 Zm00001d036483 Zm00001d027451 Zm00001d003157 Zm00001d033405 Zm00001d004497 Zm00001d014735 Zm00001d036593 Zm00001d006777 Zm00001d037140 Zm00001d007884 Zm00001d030556 Zm00001d012482 Zm00001d019288 Zm00001d032669 Zm00001d041693 Zm00001d003398 Zm00001d036263 Zm00001d002967 Zm00001d008583 Zm00001d049133 Zm00001d017176 Zm00001d004559 Zm00001d011187 Zm00001d027708 Zm00001d014386 Zm00001d033746 Zm00001d005118 Zm00001d035053 Zm00001d003601 Zm00001d017625 Zm00001d025675 Zm00001d012679 Zm00001d021387 Zm00001d029527 Zm00001d005888 Zm00001d043062 Zm00001d048334 Zm00001d048553 Zm00001d013202 Zm00001d028536 Zm00001d036084 Zm00001d015279 Zm00001d053953 Zm00001d024965 Zm00001d053909 Zm00001d040233 Zm00001d042494 Zm00001d028664 Zm00001d009987 Zm00001d053289 Zm00001d045205 Zm00001d042125 Zm00001d012050 Zm00001d002511 Zm00001d051044 Zm00001d044250 Zm00001d038328 Zm00001d052543 Zm00001d049303 Zm00001d013390 Zm00001d038514 Zm00001d006508 Zm00001d039241 Zm00001d009929 Zm00001d010231 Zm00001d040155 Zm00001d012458 Zm00001d036416 Zm00001d002109 Zm00001d025892 Zm00001d034621 Zm00001d022065 Zm00001d043655 Zm00001d049333 Zm00001d027333 Zm00001d005317 Zm00001d043294 Zm00001d042061 Zm00001d051795 Zm00001d032046 Zm00001d004531 Zm00001d025375 Zm00001d001812 Zm00001d027371 Zm00001d003785 Zm00001d031694 Zm00001d042313 Zm00001d034326 Zm00001d044639 Zm00001d011740 Zm00001d028713 Zm00001d029608 Zm00001d048890 Zm00001d014282 Zm00001d015366 Zm00001d006549 Zm00001d008706 Zm00001d029583 Zm00001d048494 Zm00001d038326 Zm00001d011629 Zm00001d025305 Zm00001d017420 Zm00001d037112 Zm00001d031058 Zm00001d051692 Zm00001d014722 Zm00001d049541 Zm00001d011993 Zm00001d041472 Zm00001d002351 Zm00001d020555 Zm00001d031431 Zm00001d044673 Zm00001d031496 Zm00001d033625 Zm00001d017085 Zm00001d020731 Zm00001d035629 Zm00001d030132 Zm00001d012035 Zm00001d018255 Zm00001d031191 Zm00001d021376 Zm00001d051527 Zm00001d006293 Zm00001d025116 Zm00001d022369 Zm00001d024343 Zm00001d041480 Zm00001d009027 Zm00001d007255 Zm00001d050694 Zm00001d053212 Zm00001d014659 Zm00001d021866 Zm00001d002406 Zm00001d016942 Zm00001d049734 Zm00001d018001 Zm00001d021909 Zm00001d006066 Zm00001d034915 Zm00001d018183 Zm00001d045482 Zm00001d033391 Zm00001d048569 Zm00001d021569 Zm00001d050082 Zm00001d048114 Zm00001d033366 Zm00001d043317 Zm00001d012884 Zm00001d004973 Zm00001d038725 Zm00001d052494 Zm00001d018072 Zm00001d006771 Zm00001d007345 Zm00001d005406 Zm00001d033555 Zm00001d024962 Zm00001d039498 Zm00001d033296 Zm00001d046277 Zm00001d032473 Zm00001d045993 Zm00001d044379 Zm00001d042425 Zm00001d002649 Zm00001d053889 Zm00001d023877 Zm00001d004861 Zm00001d016182 Zm00001d025360 Zm00001d050914 Zm00001d033428 Zm00001d004279 Zm00001d016873 Zm00001d018087 Zm00001d038084 Zm00001d039133 Zm00001d004843 Zm00001d049913 Zm00001d012231 Zm00001d036956 Zm00001d051206 Zm00001d003969 Zm00001d053743 Zm00001d044376 Zm00001d048219 Zm00001d040317 Zm00001d012902 Zm00001d029389 Zm00001d015228 Zm00001d007454 Zm00001d010463 Zm00001d004726 Zm00001d043368 Zm00001d040183 Zm00001d043483 Zm00001d040775 Zm00001d047399 Zm00001d023680 Zm00001d008602 Zm00001d016792 Zm00001d032376 Zm00001d048880 Zm00001d034516 Zm00001d018498 Zm00001d013544 Zm00001d004573 Zm00001d031127 Zm00001d018609 Zm00001d027982 Zm00001d006020 Zm00001d014820 Zm00001d047998 Zm00001d053972 Zm00001d052806 Zm00001d025352 Zm00001d015432 Zm00001d031619 Zm00001d048593 Zm00001d045950 Zm00001d016544 Zm00001d009686 Zm00001d017241 Zm00001d031808 Zm00001d043692 Zm00001d047772 Zm00001d029749 Zm00001d029645 Zm00001d039754 Zm00001d002087 Zm00001d042637 Zm00001d006481 Zm00001d034437 Zm00001d045905 Zm00001d051458 Zm00001d030993 Zm00001d032991 Zm00001d013498 Zm00001d024700 Zm00001d012656 Zm00001d011969 Zm00001d030056 Zm00001d045617 Zm00001d006659 Zm00001d048361 Zm00001d053634 Zm00001d023939 Zm00001d054002 Zm00001d023897 Zm00001d010197 Zm00001d053834 Zm00001d047744 Zm00001d017978 Zm00001d006790 Zm00001d017900 Zm00001d005884 Zm00001d029699 Zm00001d035854 Zm00001d028936 Zm00001d037174 Zm00001d038194 Zm00001d032115 Zm00001d014054 Zm00001d051337 Zm00001d051233 Zm00001d019727 Zm00001d043614 Zm00001d018133 Zm00001d002122 Zm00001d002871 Zm00001d031689 Zm00001d012301 Zm00001d038645 Zm00001d032606 Zm00001d037411 Zm00001d041707 Zm00001d051627 Zm00001d020057 GRMZM5G815453 Zm00001d035872 Zm00001d020653 Zm00001d036137 Zm00001d001908 Zm00001d021467 Zm00001d005229 Zm00001d004772 Zm00001d028062 Zm00001d038058 Zm00001d005026 Zm00001d044101 Zm00001d001791 Zm00001d028669 Zm00001d045082 Zm00001d021085 Zm00001d046535 Zm00001d030130 Zm00001d052247 Zm00001d004466 Zm00001d013736 Zm00001d009054 Zm00001d035467 Zm00001d017656 Zm00001d028802 Zm00001d008648 Zm00001d038522 Zm00001d039085 Zm00001d008479 Zm00001d042438 Zm00001d013261 Zm00001d016166 Zm00001d046474 Zm00001d014739 Zm00001d015338 Zm00001d028089 Zm00001d009557 Zm00001d044442 Zm00001d011605 Zm00001d040097 Zm00001d049031 Zm00001d027621 Zm00001d012387 Zm00001d011377 Zm00001d052344 Zm00001d029031 Zm00001d044864 Zm00001d036756 Zm00001d045972 Zm00001d022199 Zm00001d034978 Zm00001d023779 Zm00001d045951 Zm00001d033393 Zm00001d025099 Zm00001d011625 Zm00001d034017 Zm00001d028094 Zm00001d042153 Zm00001d038447 Zm00001d038914 Zm00001d016858 Zm00001d051635 Zm00001d012516 Zm00001d047356 Zm00001d031494 Zm00001d021344 Zm00001d039916 Zm00001d020869 Zm00001d019283 Zm00001d039494 Zm00001d018813 Zm00001d040829 Zm00001d039745 Zm00001d034518 Zm00001d026653 Zm00001d034842 Zm00001d002630 Zm00001d024386 Zm00001d052467 Zm00001d016291 Zm00001d050905 Zm00001d016714 Zm00001d050606 Zm00001d008520 Zm00001d026002 Zm00001d035819 Zm00001d024596 Zm00001d039338 Zm00001d040933 Zm00001d018419 Zm00001d018412 Zm00001d021791 Zm00001d023673 Zm00001d024535 Zm00001d018831 Zm00001d017809 Zm00001d038274 Zm00001d033611 Zm00001d023699 Zm00001d048053 Zm00001d034412 Zm00001d005591 Zm00001d047096 Zm00001d047908 Zm00001d026131 Zm00001d006638 Zm00001d029176 Zm00001d053306 Zm00001d052775 Zm00001d027995 Zm00001d033980 Zm00001d020560 Zm00001d051166 Zm00001d003292 Zm00001d022301 Zm00001d027347 Zm00001d012978 Zm00001d027431 Zm00001d003846 Zm00001d008737 Zm00001d027384 Zm00001d026242 Zm00001d039006 Zm00001d033092 Zm00001d041870 Zm00001d015857 Zm00001d029387 Zm00001d039870 Zm00001d023303 Zm00001d035717 Zm00001d016203 Zm00001d015221 Zm00001d012501 Zm00001d012326 Zm00001d015971 Zm00001d026705 Zm00001d006220 Zm00001d047392 Zm00001d048046 Zm00001d028389 Zm00001d021655 Zm00001d051652 Zm00001d014037 Zm00001d018180 Zm00001d038894 Zm00001d019413 Zm00001d012394 Zm00001d037446 Zm00001d047710 Zm00001d006167 Zm00001d018977 Zm00001d040129 Zm00001d013049 Zm00001d034468 Zm00001d032688 Zm00001d019793 Zm00001d006022 Zm00001d052901 Zm00001d007073 Zm00001d010759 Zm00001d016979 Zm00001d015700 Zm00001d038465 Zm00001d030639 Zm00001d010894 Zm00001d028328 Zm00001d043119 Zm00001d028771 Zm00001d042551 Zm00001d035001 Zm00001d019218 Zm00001d052139 Zm00001d053295 Zm00001d021104 Zm00001d020696 Zm00001d043018 Zm00001d029806 Zm00001d020512 Zm00001d004525 Zm00001d004929 Zm00001d006711 Zm00001d027425 Zm00001d029285 Zm00001d027892 Zm00001d052662 Zm00001d036237 Zm00001d019920 Zm00001d021400 Zm00001d045312 Zm00001d042394 Zm00001d010752 Zm00001d006525 Zm00001d021913 Zm00001d003198 Zm00001d017699 Zm00001d015977 Zm00001d038513 Zm00001d012212 Zm00001d011405 Zm00001d034443 Zm00001d012607 Zm00001d020886 Zm00001d033047 Zm00001d048760 Zm00001d025031 Zm00001d029124 Zm00001d052945 Zm00001d042665 Zm00001d034415 Zm00001d038509 Zm00001d051749 Zm00001d027320 Zm00001d028762 Zm00001d014271 Zm00001d031732 Zm00001d024454 Zm00001d047289 Zm00001d053565 Zm00001d019361 Zm00001d014601 Zm00001d003262 Zm00001d028515 Zm00001d014393 Zm00001d017380 Zm00001d028712 Zm00001d011637 Zm00001d014189 Zm00001d044648 Zm00001d052701 Zm00001d052002 Zm00001d008844 Zm00001d013627 Zm00001d011092 Zm00001d015217 Zm00001d017379 Zm00001d027885 Zm00001d043634 Zm00001d005612 Zm00001d020136 Zm00001d018623 Zm00001d051908 Zm00001d014918 Zm00001d052602 Zm00001d029164 Zm00001d043592 Zm00001d006885 Zm00001d042013 Zm00001d016156 Zm00001d050216 Zm00001d013265 Zm00001d023825 Zm00001d048566 Zm00001d048139 Zm00001d001806 Zm00001d040392 Zm00001d011518 Zm00001d005391 Zm00001d038018 Zm00001d025241 Zm00001d045029 Zm00001d022436 Zm00001d026140 Zm00001d030780 Zm00001d038930 GRMZM5G884912 Zm00001d031261 Zm00001d031268 Zm00001d009236 Zm00001d052561 Zm00001d047763 Zm00001d021518 Zm00001d044418 Zm00001d043166 Zm00001d020501 Zm00001d013241 Zm00001d021905 Zm00001d038047 Zm00001d026398 Zm00001d012553 Zm00001d036423 Zm00001d038299 Zm00001d042074 Zm00001d002548 Zm00001d037103 Zm00001d034338 Zm00001d028565 Zm00001d029151 Zm00001d038319 Zm00001d007350 Zm00001d011923 Zm00001d007080 Zm00001d052040 Zm00001d032306 Zm00001d037284 Zm00001d049174 Zm00001d048730 Zm00001d009059 Zm00001d018871 Zm00001d043789 Zm00001d040270 Zm00001d014088 Zm00001d038452 Zm00001d018904 Zm00001d017700 Zm00001d043600 Zm00001d034232 Zm00001d034968 Zm00001d027727 Zm00001d018749 Zm00001d044698 Zm00001d028707 Zm00001d051431 Zm00001d016705 Zm00001d047125 Zm00001d015377 Zm00001d014318 Zm00001d017560 Zm00001d011144 Zm00001d042217 Zm00001d005741 Zm00001d003540 Zm00001d019398 Zm00001d013384 Zm00001d037780 Zm00001d047055 Zm00001d042328 Zm00001d025067 Zm00001d007077 Zm00001d048693 Zm00001d013721 Zm00001d009372 Zm00001d012605 Zm00001d002353 Zm00001d044554 Zm00001d033674 Zm00001d041067 Zm00001d041767 Zm00001d013827 Zm00001d039091 Zm00001d012618 Zm00001d012965 Zm00001d025193 Zm00001d050346 Zm00001d047438 Zm00001d031540 Zm00001d008201 Zm00001d013728 Zm00001d026331 Zm00001d044251 Zm00001d029808 Zm00001d033397 Zm00001d051374 Zm00001d021781 Zm00001d003401 Zm00001d008724 Zm00001d031439 Zm00001d039226 Zm00001d005338 Zm00001d016977 Zm00001d039674 Zm00001d027877 Zm00001d012728 Zm00001d034425 Zm00001d028975 Zm00001d050989 Zm00001d028690 Zm00001d050955 Zm00001d012229 Zm00001d022153 Zm00001d038248 Zm00001d051676 Zm00001d006244 Zm00001d014764 Zm00001d052231 Zm00001d043491 Zm00001d023596 Zm00001d010520 Zm00001d011854 Zm00001d024833 Zm00001d010546 Zm00001d027863 Zm00001d011401 Zm00001d015839 Zm00001d042998 Zm00001d049816 Zm00001d003522 Zm00001d034675 Zm00001d053004 Zm00001d052362 Zm00001d033446 Zm00001d034438 Zm00001d051986 Zm00001d035156 Zm00001d009968 Zm00001d035090 Zm00001d013652 Zm00001d016223 Zm00001d047839 Zm00001d016793 Zm00001d011746 Zm00001d007184 Zm00001d022390 Zm00001d012504 Zm00001d027999 Zm00001d033056 Zm00001d017097 Zm00001d036455 Zm00001d048333 Zm00001d002150 Zm00001d002143 Zm00001d012995 Zm00001d002815 Zm00001d028998 Zm00001d044232 Zm00001d052242 Zm00001d020329 Zm00001d026649 Zm00001d029422 Zm00001d004977 Zm00001d049363 Zm00001d020939 Zm00001d041826 Zm00001d022088 Zm00001d011873 Zm00001d005429 Zm00001d006551 Zm00001d042185 Zm00001d035907 Zm00001d053967 Zm00001d013958 Zm00001d028611 Zm00001d033836 Zm00001d014615 Zm00001d002234 Zm00001d012382 Zm00001d013332 Zm00001d011001 Zm00001d015658 Zm00001d011458 Zm00001d020428 Zm00001d038408 Zm00001d021867 Zm00001d051094 Zm00001d034673 Zm00001d040323 Zm00001d012746 Zm00001d003115 Zm00001d037369 Zm00001d049002 Zm00001d051814 Zm00001d008662 Zm00001d019049 Zm00001d008229 Zm00001d015188 Zm00001d026678 Zm00001d019343 Zm00001d018394 Zm00001d018528 Zm00001d052746 Zm00001d026283 Zm00001d018204 Zm00001d047659 Zm00001d012031 Zm00001d044763 Zm00001d031643 Zm00001d035526 Zm00001d011503 Zm00001d018790 Zm00001d011805 Zm00001d012709 Zm00001d047501 Zm00001d040416 Zm00001d006454 Zm00001d017927 Zm00001d015092 Zm00001d040142 Zm00001d042549 Zm00001d025574 Zm00001d040232 Zm00001d029140 Zm00001d024458 Zm00001d014655 Zm00001d020790 Zm00001d018809 Zm00001d024470 Zm00001d037227 Zm00001d014669 Zm00001d031485 Zm00001d018477 Zm00001d013798 Zm00001d029078 Zm00001d003451 Zm00001d012361 Zm00001d020454 Zm00001d014733 Zm00001d038910 Zm00001d010388 Zm00001d018339 Zm00001d014717 Zm00001d004042 Zm00001d026086 Zm00001d036482 Zm00001d015008 Zm00001d015690 Zm00001d037118 Zm00001d053123 Zm00001d045382 Zm00001d012527 Zm00001d015361 Zm00001d024420 Zm00001d036534 Zm00001d034543 Zm00001d038618 Zm00001d020063 Zm00001d006910 Zm00001d005658 Zm00001d040960 Zm00001d033553 Zm00001d042867 Zm00001d013274 Zm00001d038200 Zm00001d048317 Zm00001d012079 Zm00001d043975 Zm00001d025538 Zm00001d011588 Zm00001d017461 Zm00001d027365 Zm00001d021298 Zm00001d003911 Zm00001d033680 Zm00001d044204 Zm00001d046583 Zm00001d005502 Zm00001d022252 Zm00001d046751 Zm00001d053954 Zm00001d052519 Zm00001d016922 Zm00001d008601 Zm00001d034517 Zm00001d025950 Zm00001d020772 Zm00001d020267 Zm00001d032170 Zm00001d038533 Zm00001d044823 Zm00001d048532 Zm00001d024679 Zm00001d025864 Zm00001d037711 Zm00001d046234 Zm00001d039922 Zm00001d041455 Zm00001d011890 Zm00001d013314 Zm00001d014961 Zm00001d042045 Zm00001d034736 Zm00001d019327 Zm00001d014664 Zm00001d011788 Zm00001d007173 Zm00001d034618 Zm00001d006614 Zm00001d022092 Zm00001d045002 Zm00001d021802 Zm00001d047241 Zm00001d012621 Zm00001d048556 Zm00001d043911 Zm00001d025065 Zm00001d040783 Zm00001d043741 Zm00001d002814 Zm00001d026690 Zm00001d039053 Zm00001d050167 Zm00001d034012 Zm00001d007379 Zm00001d004417 Zm00001d014657 Zm00001d038377 Zm00001d033606 Zm00001d043296 Zm00001d042470 Zm00001d022053 Zm00001d037367 Zm00001d018830 Zm00001d048962 Zm00001d028025 Zm00001d037794 Zm00001d029803 Zm00001d053880 Zm00001d024744 Zm00001d004346 Zm00001d020845 Zm00001d034257 Zm00001d003913 Zm00001d018639 Zm00001d046812 GRMZM5G874448 Zm00001d049099 Zm00001d006205 Zm00001d027291 Zm00001d006016 Zm00001d044705 Zm00001d016950 Zm00001d006291 Zm00001d043233 Zm00001d006443 Zm00001d016134 Zm00001d004009 Zm00001d027581 Zm00001d033579 Zm00001d033504 Zm00001d018627 Zm00001d052022 Zm00001d015129 Zm00001d013919 Zm00001d012748 Zm00001d052033 Zm00001d039595 Zm00001d033777 Zm00001d017212 Zm00001d038638 Zm00001d034152 Zm00001d027963 Zm00001d036790 Zm00001d042380 Zm00001d038793 Zm00001d012883 Zm00001d021732 Zm00001d046560 Zm00001d046817 Zm00001d002405 Zm00001d020543 Zm00001d020537 Zm00001d006755 Zm00001d053157 Zm00001d042725 Zm00001d034033 Zm00001d007502 Zm00001d023318 Zm00001d011355 Zm00001d013534 Zm00001d004733 Zm00001d033483 Zm00001d043968 Zm00001d001941 Zm00001d012894 Zm00001d036708 Zm00001d025195 Zm00001d012932 Zm00001d039639 Zm00001d006247 Zm00001d033564 Zm00001d038381 Zm00001d051930 Zm00001d003007 Zm00001d041961 Zm00001d009918 Zm00001d034221 Zm00001d030584 Zm00001d032144 Zm00001d012970 Zm00001d034022 Zm00001d006704 Zm00001d020409 Zm00001d006199 Zm00001d035503 Zm00001d045336 Zm00001d041839 Zm00001d052433 Zm00001d032455 Zm00001d001972 Zm00001d011709 Zm00001d042455 Zm00001d041972 Zm00001d039967 Zm00001d047896 Zm00001d017899 Zm00001d013194 Zm00001d020910 Zm00001d007640 Zm00001d052618 Zm00001d005859 Zm00001d025218 Zm00001d039077 Zm00001d028547 Zm00001d011297 Zm00001d028399 Zm00001d027509 Zm00001d042147 Zm00001d051939 Zm00001d027842 Zm00001d053927 Zm00001d032377 Zm00001d015968 Zm00001d045021 Zm00001d010698 Zm00001d024169 Zm00001d039958 Zm00001d028880 Zm00001d047020 Zm00001d053197 Zm00001d049349 Zm00001d005828 Zm00001d006645 Zm00001d043104 Zm00001d044547 Zm00001d020694 Zm00001d008380 Zm00001d028753 Zm00001d053236 Zm00001d016982 Zm00001d033210 Zm00001d022111 GRMZM5G804708 Zm00001d029720 Zm00001d016176 Zm00001d010236 Zm00001d024220 Zm00001d010758 Zm00001d048622 Zm00001d051511 Zm00001d031781 Zm00001d017387 Zm00001d032399 Zm00001d047732 Zm00001d052104 Zm00001d046399 Zm00001d023543 Zm00001d008397 Zm00001d037947 Zm00001d024697 Zm00001d007072 Zm00001d053763 Zm00001d043378 Zm00001d016299 Zm00001d044060 Zm00001d009596 Zm00001d027387 Zm00001d003857 Zm00001d041772 GRMZM5G811749 Zm00001d046132 Zm00001d033324 Zm00001d018925 Zm00001d020298 Zm00001d032557 Zm00001d004411 Zm00001d016846 Zm00001d053452 Zm00001d046632 Zm00001d040467 Zm00001d025400 Zm00001d051854 Zm00001d039372 Zm00001d018019 Zm00001d050062 Zm00001d009907 Zm00001d021730 Zm00001d049079 Zm00001d024712 Zm00001d022581 Zm00001d020532 Zm00001d050076 Zm00001d033853 Zm00001d011180 Zm00001d030105 Zm00001d038174 Zm00001d003673 Zm00001d037221 Zm00001d028423 Zm00001d023238 Zm00001d007902 Zm00001d033837 Zm00001d025167 Zm00001d032114 Zm00001d022113 Zm00001d027816 Zm00001d003807 Zm00001d012765 Zm00001d035031 Zm00001d044642 Zm00001d002624 Zm00001d002374 Zm00001d019816 Zm00001d006555 Zm00001d029684 Zm00001d044130 Zm00001d005064 Zm00001d039315 Zm00001d039185 Zm00001d003272 Zm00001d008977 Zm00001d003058 Zm00001d052357 Zm00001d052816 Zm00001d004452 Zm00001d024171 Zm00001d033815 Zm00001d047848 Zm00001d031997 Zm00001d043188 Zm00001d036638 Zm00001d037057 Zm00001d008498 Zm00001d015613 Zm00001d052493 Zm00001d046866 Zm00001d010892 Zm00001d011555 Zm00001d040450 Zm00001d037874 Zm00001d007790 Zm00001d052767 Zm00001d035869 Zm00001d051869 Zm00001d043598 Zm00001d019042 Zm00001d032787 Zm00001d039913 Zm00001d019765 |
| Zhengzhou | 1109 | Zm00001d013669 Zm00001d036900 Zm00001d031210 Zm00001d010950 Zm00001d053936 Zm00001d014108 Zm00001d028951 Zm00001d014944 Zm00001d001911 Zm00001d008398 Zm00001d012357 Zm00001d038922 Zm00001d012446 Zm00001d034103 Zm00001d021635 Zm00001d016477 Zm00001d034717 Zm00001d009709 Zm00001d013358 Zm00001d037840 Zm00001d036571 Zm00001d047502 Zm00001d015130 Zm00001d044451 Zm00001d018595 Zm00001d011811 Zm00001d037756 Zm00001d018275 Zm00001d025449 Zm00001d032160 Zm00001d023987 Zm00001d049163 Zm00001d029393 Zm00001d009589 Zm00001d008845 Zm00001d047057 Zm00001d052715 Zm00001d020158 Zm00001d053966 Zm00001d021119 Zm00001d021565 Zm00001d025781 Zm00001d009317 Zm00001d005144 Zm00001d014124 Zm00001d040901 Zm00001d025513 Zm00001d054012 Zm00001d017166 Zm00001d005662 Zm00001d026381 Zm00001d033497 Zm00001d039038 Zm00001d048174 Zm00001d040766 Zm00001d003793 Zm00001d002338 Zm00001d021309 Zm00001d010914 Zm00001d034059 Zm00001d033383 Zm00001d033969 Zm00001d048310 Zm00001d028053 Zm00001d028238 Zm00001d013747 Zm00001d021321 Zm00001d003539 Zm00001d045747 Zm00001d025174 Zm00001d035371 Zm00001d012175 Zm00001d012825 Zm00001d003894 Zm00001d052216 Zm00001d044447 Zm00001d038929 Zm00001d024154 Zm00001d036496 Zm00001d032539 Zm00001d012771 Zm00001d011994 Zm00001d016766 Zm00001d036422 Zm00001d021810 Zm00001d034957 Zm00001d025692 Zm00001d028840 Zm00001d017830 Zm00001d026284 Zm00001d025112 Zm00001d043963 Zm00001d044628 Zm00001d029462 Zm00001d022335 Zm00001d017089 Zm00001d037393 Zm00001d034196 Zm00001d014654 Zm00001d025327 Zm00001d032707 Zm00001d017403 Zm00001d044597 Zm00001d032725 Zm00001d005536 Zm00001d020717 Zm00001d009849 Zm00001d048540 Zm00001d030106 Zm00001d012402 Zm00001d009515 Zm00001d041180 Zm00001d027460 Zm00001d048020 Zm00001d031586 Zm00001d024393 Zm00001d006256 Zm00001d008268 Zm00001d044277 Zm00001d006455 Zm00001d008689 Zm00001d046460 Zm00001d002642 Zm00001d016878 Zm00001d023299 Zm00001d029118 Zm00001d042033 Zm00001d049615 Zm00001d009808 Zm00001d026675 Zm00001d015308 Zm00001d045965 Zm00001d002105 Zm00001d039250 Zm00001d013915 Zm00001d038756 Zm00001d051684 Zm00001d026257 Zm00001d018127 Zm00001d008345 Zm00001d042234 Zm00001d049922 Zm00001d008525 Zm00001d028298 Zm00001d043069 Zm00001d013869 Zm00001d003864 Zm00001d025816 Zm00001d045706 Zm00001d037736 Zm00001d035321 Zm00001d033371 Zm00001d013081 Zm00001d034010 Zm00001d016440 Zm00001d029776 Zm00001d029174 Zm00001d038481 Zm00001d043856 Zm00001d023536 Zm00001d021401 Zm00001d049188 Zm00001d011150 Zm00001d039578 Zm00001d044331 Zm00001d020688 Zm00001d020744 Zm00001d034578 Zm00001d016105 Zm00001d009161 Zm00001d044713 Zm00001d035722 Zm00001d006565 Zm00001d027309 Zm00001d002978 Zm00001d023974 Zm00001d031542 Zm00001d047617 Zm00001d015799 Zm00001d049873 Zm00001d035589 Zm00001d042007 Zm00001d012480 Zm00001d048419 Zm00001d007388 Zm00001d003415 Zm00001d014278 Zm00001d042057 Zm00001d020511 Zm00001d011145 Zm00001d035859 Zm00001d031317 Zm00001d015036 Zm00001d001765 Zm00001d007261 Zm00001d018409 Zm00001d040344 Zm00001d022083 Zm00001d051754 Zm00001d027706 Zm00001d050910 Zm00001d004655 Zm00001d021779 Zm00001d039597 Zm00001d010693 Zm00001d010868 Zm00001d010198 Zm00001d029646 Zm00001d048314 Zm00001d038190 Zm00001d048008 Zm00001d043669 Zm00001d044194 Zm00001d047407 Zm00001d008581 Zm00001d043052 Zm00001d021084 Zm00001d033927 Zm00001d048604 Zm00001d029649 Zm00001d051800 Zm00001d030382 Zm00001d027323 Zm00001d031420 Zm00001d037264 Zm00001d029429 Zm00001d034659 Zm00001d018938 Zm00001d012634 Zm00001d021395 Zm00001d036098 Zm00001d006374 Zm00001d003729 Zm00001d025679 Zm00001d028173 Zm00001d041438 Zm00001d040265 Zm00001d010209 Zm00001d025307 Zm00001d017201 Zm00001d005911 Zm00001d011199 Zm00001d005396 Zm00001d014785 Zm00001d001799 Zm00001d003291 Zm00001d039095 Zm00001d033594 Zm00001d029490 Zm00001d005205 Zm00001d015463 Zm00001d054077 Zm00001d008326 Zm00001d002504 Zm00001d002301 Zm00001d002938 Zm00001d009749 Zm00001d038158 Zm00001d052475 Zm00001d011595 Zm00001d050121 Zm00001d051605 Zm00001d023305 Zm00001d023767 Zm00001d011220 Zm00001d031851 Zm00001d017174 Zm00001d024951 Zm00001d049860 Zm00001d049083 Zm00001d001828 Zm00001d038473 Zm00001d019595 Zm00001d001809 Zm00001d032064 Zm00001d032739 Zm00001d045518 Zm00001d012052 Zm00001d028556 Zm00001d049225 Zm00001d031894 Zm00001d002051 Zm00001d018864 Zm00001d038012 Zm00001d020903 Zm00001d042899 Zm00001d033467 Zm00001d010806 Zm00001d049391 Zm00001d012688 Zm00001d053728 Zm00001d023340 Zm00001d046344 Zm00001d053146 Zm00001d009954 Zm00001d021908 Zm00001d017888 Zm00001d033635 Zm00001d025273 Zm00001d013512 Zm00001d042760 Zm00001d035297 Zm00001d001940 Zm00001d026345 Zm00001d022168 Zm00001d052713 Zm00001d050862 Zm00001d034764 Zm00001d011512 Zm00001d011828 Zm00001d013290 Zm00001d013505 Zm00001d019641 Zm00001d039740 Zm00001d007096 Zm00001d010714 Zm00001d005203 Zm00001d046060 Zm00001d001959 Zm00001d004903 Zm00001d050610 Zm00001d018147 Zm00001d041069 Zm00001d029664 Zm00001d022487 Zm00001d035634 Zm00001d003566 Zm00001d043795 Zm00001d028619 Zm00001d014532 Zm00001d048084 Zm00001d032058 Zm00001d002770 Zm00001d018024 Zm00001d003258 Zm00001d032357 Zm00001d021520 Zm00001d042750 Zm00001d043182 Zm00001d043516 Zm00001d014772 Zm00001d030109 Zm00001d028620 Zm00001d014775 Zm00001d050350 Zm00001d002680 Zm00001d034634 Zm00001d021579 Zm00001d036546 Zm00001d029203 Zm00001d021031 Zm00001d051861 Zm00001d050119 Zm00001d021676 Zm00001d018383 Zm00001d052800 Zm00001d022569 Zm00001d051380 Zm00001d028918 Zm00001d036624 Zm00001d002065 Zm00001d043307 Zm00001d017941 Zm00001d013642 Zm00001d038048 Zm00001d018719 Zm00001d054052 Zm00001d044083 Zm00001d006552 Zm00001d008549 Zm00001d005358 Zm00001d037871 Zm00001d032761 Zm00001d011113 Zm00001d002033 Zm00001d005647 Zm00001d017954 Zm00001d032732 Zm00001d018765 Zm00001d008597 Zm00001d018103 Zm00001d030765 Zm00001d031937 Zm00001d015714 Zm00001d017199 Zm00001d029543 Zm00001d024318 Zm00001d034368 Zm00001d006845 Zm00001d043682 Zm00001d012701 Zm00001d029034 Zm00001d025025 Zm00001d040617 Zm00001d052747 Zm00001d046664 Zm00001d025713 Zm00001d038266 Zm00001d002126 Zm00001d037084 Zm00001d024499 Zm00001d026516 Zm00001d031740 Zm00001d038107 Zm00001d036798 Zm00001d033322 Zm00001d014418 Zm00001d027957 Zm00001d021708 Zm00001d040445 Zm00001d004015 Zm00001d014894 Zm00001d013245 Zm00001d037452 Zm00001d019562 Zm00001d006133 Zm00001d005468 Zm00001d024640 Zm00001d005302 Zm00001d034361 Zm00001d021822 Zm00001d017333 Zm00001d020087 Zm00001d049286 Zm00001d033174 Zm00001d050245 Zm00001d038526 Zm00001d037326 Zm00001d003276 Zm00001d008856 Zm00001d006471 Zm00001d010577 Zm00001d006329 Zm00001d002181 Zm00001d036274 Zm00001d039141 Zm00001d015006 Zm00001d011792 Zm00001d049857 Zm00001d003904 Zm00001d012404 Zm00001d006031 Zm00001d027612 Zm00001d006864 Zm00001d025398 Zm00001d012674 Zm00001d033215 Zm00001d051163 Zm00001d033307 Zm00001d020780 Zm00001d034758 Zm00001d020787 Zm00001d011536 Zm00001d044470 Zm00001d030544 Zm00001d052944 Zm00001d035253 Zm00001d027444 Zm00001d047757 Zm00001d016794 Zm00001d049723 Zm00001d032683 Zm00001d025746 Zm00001d001929 Zm00001d042599 Zm00001d011717 Zm00001d003267 Zm00001d053371 Zm00001d033642 Zm00001d008847 Zm00001d033303 Zm00001d014572 Zm00001d033454 Zm00001d023267 Zm00001d007090 Zm00001d024500 Zm00001d049159 Zm00001d010000 Zm00001d012155 Zm00001d021508 Zm00001d025939 Zm00001d023249 Zm00001d016361 Zm00001d039512 Zm00001d044987 Zm00001d008171 Zm00001d002054 Zm00001d033764 Zm00001d038199 Zm00001d038072 Zm00001d006106 Zm00001d042525 Zm00001d025469 Zm00001d032505 Zm00001d019372 Zm00001d010309 Zm00001d041161 Zm00001d031581 Zm00001d024807 Zm00001d022175 Zm00001d011393 Zm00001d053636 Zm00001d012398 Zm00001d002550 Zm00001d041730 Zm00001d014865 Zm00001d024277 Zm00001d029657 Zm00001d017203 Zm00001d040141 Zm00001d030011 Zm00001d039200 Zm00001d005329 Zm00001d010480 Zm00001d002980 Zm00001d028436 Zm00001d019329 Zm00001d051125 Zm00001d006672 Zm00001d044699 Zm00001d046004 Zm00001d041112 Zm00001d053642 Zm00001d002788 Zm00001d018891 Zm00001d002423 Zm00001d018515 Zm00001d034550 Zm00001d044551 Zm00001d020923 Zm00001d018435 Zm00001d005212 Zm00001d035072 Zm00001d052850 Zm00001d031266 Zm00001d013966 Zm00001d011147 Zm00001d001767 Zm00001d010159 Zm00001d015649 Zm00001d016811 Zm00001d017468 Zm00001d049787 Zm00001d003664 Zm00001d022296 Zm00001d008759 Zm00001d028903 Zm00001d036054 Zm00001d040008 Zm00001d011602 Zm00001d049743 Zm00001d004469 Zm00001d017883 Zm00001d034180 Zm00001d003123 Zm00001d014447 Zm00001d024625 Zm00001d003392 Zm00001d050384 Zm00001d048041 Zm00001d017595 Zm00001d017956 Zm00001d010512 Zm00001d034629 Zm00001d053547 Zm00001d012417 Zm00001d008871 Zm00001d028540 Zm00001d025275 Zm00001d018740 Zm00001d047661 Zm00001d030153 Zm00001d033494 Zm00001d039256 Zm00001d027788 Zm00001d048480 Zm00001d044647 Zm00001d013863 Zm00001d007369 Zm00001d038170 Zm00001d033849 Zm00001d029329 Zm00001d027871 Zm00001d047983 Zm00001d011152 Zm00001d051525 Zm00001d043309 Zm00001d028396 Zm00001d051085 Zm00001d003002 Zm00001d016854 Zm00001d044844 Zm00001d050314 Zm00001d022003 Zm00001d028728 Zm00001d029627 Zm00001d051553 Zm00001d042266 Zm00001d033993 Zm00001d034442 Zm00001d030264 Zm00001d044333 Zm00001d046388 Zm00001d028757 Zm00001d034719 Zm00001d012100 Zm00001d019629 Zm00001d026485 Zm00001d034667 Zm00001d037856 Zm00001d028450 Zm00001d028356 Zm00001d041727 Zm00001d053799 Zm00001d046974 Zm00001d023327 Zm00001d013186 Zm00001d046801 Zm00001d040839 Zm00001d044979 Zm00001d026144 Zm00001d021557 Zm00001d021916 Zm00001d028375 Zm00001d013738 Zm00001d018367 Zm00001d030770 Zm00001d048059 Zm00001d048979 Zm00001d015151 Zm00001d012556 Zm00001d005542 Zm00001d053802 Zm00001d023504 Zm00001d033502 Zm00001d002733 Zm00001d029557 Zm00001d016198 Zm00001d049572 Zm00001d014650 Zm00001d039354 Zm00001d003646 Zm00001d004978 Zm00001d044593 Zm00001d008303 Zm00001d011256 Zm00001d044175 Zm00001d011102 Zm00001d048577 Zm00001d035974 Zm00001d044302 Zm00001d023296 Zm00001d015115 Zm00001d047563 Zm00001d037775 Zm00001d023733 Zm00001d048655 Zm00001d036293 Zm00001d053555 Zm00001d023792 Zm00001d016029 Zm00001d030666 Zm00001d002878 Zm00001d026515 Zm00001d053688 Zm00001d051932 Zm00001d043649 Zm00001d025711 Zm00001d047638 Zm00001d007956 Zm00001d009612 Zm00001d047863 Zm00001d006459 Zm00001d019631 Zm00001d034620 Zm00001d042213 Zm00001d028530 Zm00001d030760 Zm00001d038054 Zm00001d047498 Zm00001d050187 Zm00001d053979 Zm00001d048574 Zm00001d044226 Zm00001d035844 Zm00001d014314 Zm00001d048562 Zm00001d043098 Zm00001d018377 Zm00001d052034 Zm00001d029380 Zm00001d046915 Zm00001d037731 Zm00001d011160 Zm00001d010447 Zm00001d027557 Zm00001d016374 Zm00001d008739 Zm00001d049120 Zm00001d037258 Zm00001d036541 Zm00001d016863 Zm00001d020374 Zm00001d012585 Zm00001d030301 Zm00001d039747 Zm00001d049449 Zm00001d035199 Zm00001d034858 Zm00001d021138 Zm00001d007295 Zm00001d044401 Zm00001d025665 Zm00001d046476 Zm00001d008249 Zm00001d028111 Zm00001d021645 Zm00001d025232 Zm00001d040286 Zm00001d026358 Zm00001d047875 Zm00001d009627 Zm00001d043573 Zm00001d005451 Zm00001d043955 Zm00001d033484 Zm00001d005812 Zm00001d021488 Zm00001d033925 Zm00001d042603 Zm00001d023239 Zm00001d033578 Zm00001d050489 Zm00001d004913 Zm00001d015842 Zm00001d036609 Zm00001d012428 Zm00001d027486 Zm00001d037873 Zm00001d012511 Zm00001d019531 Zm00001d004472 Zm00001d037066 Zm00001d021142 Zm00001d031572 Zm00001d011321 Zm00001d046145 Zm00001d013447 Zm00001d033186 Zm00001d049910 Zm00001d009599 Zm00001d025913 Zm00001d007745 Zm00001d034278 Zm00001d011929 Zm00001d052391 Zm00001d010946 Zm00001d047468 Zm00001d042699 Zm00001d012917 Zm00001d028786 Zm00001d037463 Zm00001d011601 Zm00001d021067 Zm00001d035030 Zm00001d045152 Zm00001d039305 Zm00001d001831 Zm00001d042676 Zm00001d008632 Zm00001d028902 Zm00001d029206 Zm00001d041175 Zm00001d018792 Zm00001d042275 Zm00001d011177 Zm00001d003812 Zm00001d024936 Zm00001d018326 Zm00001d033065 Zm00001d031659 Zm00001d047582 Zm00001d025165 Zm00001d016520 Zm00001d020274 Zm00001d007483 Zm00001d033025 Zm00001d022352 Zm00001d045160 Zm00001d009071 Zm00001d011034 Zm00001d008591 Zm00001d019195 Zm00001d030165 Zm00001d020921 Zm00001d040831 Zm00001d008322 Zm00001d033535 Zm00001d047302 Zm00001d045975 Zm00001d040409 Zm00001d051473 Zm00001d026122 Zm00001d047946 Zm00001d051552 Zm00001d024187 Zm00001d007084 Zm00001d042976 Zm00001d037712 Zm00001d024890 Zm00001d034944 Zm00001d013025 Zm00001d002905 Zm00001d020228 Zm00001d042442 Zm00001d051021 Zm00001d028606 Zm00001d038681 Zm00001d049135 Zm00001d013582 Zm00001d002385 Zm00001d012684 Zm00001d021385 Zm00001d008259 Zm00001d050886 Zm00001d038449 Zm00001d033975 Zm00001d024787 Zm00001d021363 Zm00001d014766 Zm00001d025316 Zm00001d041077 Zm00001d008772 Zm00001d049048 Zm00001d030665 Zm00001d006128 Zm00001d009761 Zm00001d011770 Zm00001d032044 Zm00001d034028 Zm00001d041004 Zm00001d048499 Zm00001d020740 Zm00001d048346 Zm00001d040479 Zm00001d037538 Zm00001d050174 Zm00001d003822 Zm00001d003328 Zm00001d033107 Zm00001d020139 Zm00001d048199 Zm00001d051062 Zm00001d016784 Zm00001d022637 Zm00001d036982 Zm00001d029281 Zm00001d053290 Zm00001d053633 Zm00001d053778 Zm00001d024709 Zm00001d012140 Zm00001d041246 Zm00001d047388 Zm00001d043548 Zm00001d053648 Zm00001d032380 Zm00001d019703 Zm00001d009756 Zm00001d029489 Zm00001d006874 Zm00001d037249 Zm00001d047779 Zm00001d045448 Zm00001d033620 Zm00001d015975 Zm00001d039387 Zm00001d021318 Zm00001d016837 Zm00001d049007 Zm00001d035574 Zm00001d033647 Zm00001d044335 Zm00001d033912 Zm00001d052680 Zm00001d034760 Zm00001d018430 Zm00001d012929 Zm00001d037427 Zm00001d019865 Zm00001d010646 Zm00001d028920 Zm00001d009549 Zm00001d013840 Zm00001d049830 Zm00001d051323 Zm00001d016577 Zm00001d008233 Zm00001d052989 Zm00001d047988 Zm00001d040357 Zm00001d008617 Zm00001d041594 Zm00001d053010 Zm00001d002027 Zm00001d044224 Zm00001d034600 Zm00001d035731 Zm00001d035211 Zm00001d015948 Zm00001d043224 Zm00001d010887 Zm00001d003405 Zm00001d025420 Zm00001d047456 Zm00001d025425 Zm00001d026366 Zm00001d043348 Zm00001d006160 Zm00001d048471 Zm00001d041203 Zm00001d016694 Zm00001d025581 Zm00001d014039 Zm00001d028377 Zm00001d051965 Zm00001d029208 Zm00001d041305 Zm00001d042596 Zm00001d051934 Zm00001d052325 Zm00001d012628 Zm00001d030739 Zm00001d052964 Zm00001d032500 Zm00001d041549 Zm00001d048147 Zm00001d037448 Zm00001d010241 Zm00001d010530 Zm00001d025132 Zm00001d039072 Zm00001d021587 Zm00001d006398 Zm00001d007118 Zm00001d019934 Zm00001d053553 Zm00001d044287 Zm00001d013240 Zm00001d038021 Zm00001d042799 Zm00001d027768 Zm00001d035776 Zm00001d015990 Zm00001d003511 Zm00001d053184 Zm00001d037104 Zm00001d048285 Zm00001d005694 Zm00001d039726 Zm00001d006457 Zm00001d050063 Zm00001d025571 Zm00001d049282 Zm00001d008347 Zm00001d030409 Zm00001d034401 Zm00001d041597 Zm00001d048824 Zm00001d044186 Zm00001d014261 Zm00001d013593 Zm00001d043736 Zm00001d053816 Zm00001d020133 Zm00001d046492 Zm00001d019644 Zm00001d003509 Zm00001d025967 Zm00001d053067 Zm00001d034665 Zm00001d015256 Zm00001d048207 Zm00001d036651 Zm00001d049376 Zm00001d011761 Zm00001d050518 Zm00001d038948 Zm00001d043006 Zm00001d013429 Zm00001d027633 Zm00001d025435 Zm00001d035987 Zm00001d036679 Zm00001d048190 Zm00001d024906 Zm00001d007762 Zm00001d008640 Zm00001d052612 Zm00001d037220 Zm00001d026348 Zm00001d037546 Zm00001d006868 Zm00001d022558 Zm00001d031940 Zm00001d015389 Zm00001d009997 Zm00001d045351 Zm00001d044812 Zm00001d041917 Zm00001d016496 Zm00001d033171 Zm00001d013781 Zm00001d014159 Zm00001d020011 Zm00001d032326 Zm00001d052442 Zm00001d018144 Zm00001d038440 Zm00001d034954 Zm00001d038672 Zm00001d037900 Zm00001d009539 Zm00001d012996 Zm00001d007667 Zm00001d038690 Zm00001d037465 Zm00001d028197 Zm00001d028269 Zm00001d008996 Zm00001d003896 Zm00001d031734 Zm00001d017894 Zm00001d031534 Zm00001d014043 Zm00001d048404 Zm00001d023384 Zm00001d007501 Zm00001d033378 Zm00001d015102 Zm00001d010210 Zm00001d003153 Zm00001d010915 Zm00001d007179 Zm00001d007938 Zm00001d013523 Zm00001d005373 Zm00001d031338 Zm00001d046305 Zm00001d017877 Zm00001d034148 Zm00001d038272 Zm00001d042908 Zm00001d039362 Zm00001d037842 Zm00001d005479 Zm00001d012447 Zm00001d039218 Zm00001d004489 Zm00001d052009 Zm00001d048988 Zm00001d040056 Zm00001d002615 Zm00001d051595 Zm00001d009211 Zm00001d047310 Zm00001d041632 Zm00001d043325 Zm00001d025180 Zm00001d037944 Zm00001d016131 Zm00001d043185 Zm00001d005498 Zm00001d047307 Zm00001d019062 Zm00001d045463 Zm00001d006566 Zm00001d032231 Zm00001d009661 Zm00001d012467 Zm00001d034525 Zm00001d038797 Zm00001d024466 Zm00001d039350 Zm00001d049673 Zm00001d013744 Zm00001d044445 Zm00001d005248 Zm00001d034886 Zm00001d048637 Zm00001d012640 Zm00001d001864 Zm00001d041979 Zm00001d037875 Zm00001d025808 Zm00001d036630 Zm00001d021245 Zm00001d017887 Zm00001d037291 Zm00001d052886 Zm00001d028570 Zm00001d017353 Zm00001d053450 Zm00001d044284 Zm00001d020380 Zm00001d022465 Zm00001d013282 Zm00001d042848 Zm00001d017555 Zm00001d054107 Zm00001d048271 Zm00001d012826 Zm00001d010720 Zm00001d045124 Zm00001d042639 Zm00001d047828 Zm00001d031186 Zm00001d040449 Zm00001d023521 Zm00001d053994 Zm00001d039625 Zm00001d015890 Zm00001d019052 Zm00001d016439 Zm00001d052138 Zm00001d033109 Zm00001d008906 Zm00001d030955 Zm00001d003552 Zm00001d021902 Zm00001d027376 Zm00001d018979 Zm00001d052184 Zm00001d049664 Zm00001d039656 Zm00001d051067 Zm00001d024593 Zm00001d045500 Zm00001d021861 Zm00001d020419 Zm00001d042709 Zm00001d018084 Zm00001d004463 Zm00001d028749 Zm00001d045383 Zm00001d011179 Zm00001d009163 Zm00001d047904 Zm00001d007533 Zm00001d031967 Zm00001d036485 Zm00001d003246 Zm00001d014943 Zm00001d048324 Zm00001d007060 Zm00001d014186 Zm00001d038722 Zm00001d046930 Zm00001d020776 Zm00001d036674 Zm00001d010212 Zm00001d035949 Zm00001d011979 Zm00001d012044 Zm00001d017728 Zm00001d037714 Zm00001d027405 Zm00001d047754 Zm00001d034934 Zm00001d017976 Zm00001d034597 Zm00001d004172 Zm00001d026691 Zm00001d012954 Zm00001d011526 Zm00001d014243 Zm00001d003428 Zm00001d043912 Zm00001d045577 Zm00001d049500 Zm00001d037927 Zm00001d010262 Zm00001d051896 Zm00001d038839 Zm00001d053589 Zm00001d017352 Zm00001d052321 Zm00001d029129 Zm00001d042954 Zm00001d031065 Zm00001d017458 Zm00001d020932 Zm00001d034868 Zm00001d010834 Zm00001d039059 Zm00001d012086 Zm00001d008564 Zm00001d025286 Zm00001d021087 Zm00001d021267 Zm00001d035542 Zm00001d023391 Zm00001d008468 Zm00001d008642 Zm00001d047726 Zm00001d028194 Zm00001d018332 Zm00001d034714 Zm00001d003166 Zm00001d037306 Zm00001d023270 Zm00001d003790 Zm00001d006822 Zm00001d014663 Zm00001d004857 Zm00001d049187 Zm00001d020441 Zm00001d038619 Zm00001d037243 Zm00001d002085 Zm00001d027573 Zm00001d037601 Zm00001d005898 Zm00001d046749 Zm00001d007133 Zm00001d014626 Zm00001d018403 Zm00001d046700 Zm00001d020510 Zm00001d017654 Zm00001d013125 Zm00001d027325 Zm00001d002267 Zm00001d038396 Zm00001d023979 Zm00001d020519 Zm00001d042651 Zm00001d006302 Zm00001d051669 Zm00001d006175 Zm00001d033786 Zm00001d019069 Zm00001d018122 Zm00001d011914 Zm00001d046405 Zm00001d052308 Zm00001d017802 Zm00001d033190 Zm00001d049761 Zm00001d032591 Zm00001d031701 Zm00001d005501 Zm00001d044197 Zm00001d026505 Zm00001d050258 Zm00001d035862 Zm00001d008594 Zm00001d031660 Zm00001d036224 Zm00001d045512 Zm00001d032156 Zm00001d051288 Zm00001d019076 Zm00001d029846 Zm00001d029301 Zm00001d003986 Zm00001d011158 Zm00001d021261 Zm00001d041088 Zm00001d020913 Zm00001d014557 Zm00001d028655 Zm00001d017323 Zm00001d047239 Zm00001d028809 Zm00001d025103 Zm00001d024876 Zm00001d036927 Zm00001d045655 Zm00001d049141 Zm00001d041092 Zm00001d020687 Zm00001d025304 Zm00001d004644 Zm00001d025413 Zm00001d002715 Zm00001d046663 Zm00001d008311 Zm00001d040264 Zm00001d016365 Zm00001d040988 Zm00001d042259 Zm00001d007783 Zm00001d051898 Zm00001d016237 Zm00001d043619 Zm00001d029740 Zm00001d021430 Zm00001d038776 Zm00001d021170 Zm00001d049712 Zm00001d011941 Zm00001d010522 Zm00001d015118 Zm00001d043267 Zm00001d035329 Zm00001d017363 Zm00001d003357 Zm00001d019348 Zm00001d047349 Zm00001d050238 Zm00001d023820 Zm00001d040477 Zm00001d034462 Zm00001d053301 Zm00001d036758 Zm00001d038687 Zm00001d029606 Zm00001d035186 Zm00001d044826 Zm00001d013658 Zm00001d036760 Zm00001d012275 Zm00001d024804 Zm00001d010574 Zm00001d031327 Zm00001d011750 Zm00001d019577 Zm00001d021778 Zm00001d013107 Zm00001d003797 Zm00001d002944 Zm00001d013426 Zm00001d037760 Zm00001d007320 Zm00001d023311 Zm00001d044273 Zm00001d046385 Zm00001d002018 Zm00001d047533 Zm00001d038082 Zm00001d049991 Zm00001d048081 Zm00001d012103 Zm00001d003106 Zm00001d017094 Zm00001d049703 Zm00001d021021 Zm00001d013707 Zm00001d017175 Zm00001d048050 Zm00001d004383 Zm00001d035540 Zm00001d012289 Zm00001d001808 Zm00001d026134 Zm00001d010678 Zm00001d025946 Zm00001d040641 Zm00001d043780 Zm00001d048323 Zm00001d039065 Zm00001d043607 Zm00001d028571 Zm00001d002684 Zm00001d038173 Zm00001d036640 Zm00001d020201 Zm00001d006242 Zm00001d020804 Zm00001d007300 Zm00001d038880 Zm00001d049680 Zm00001d017351 Zm00001d046553 Zm00001d010478 Zm00001d042535 Zm00001d021878 Zm00001d042567 Zm00001d012768 Zm00001d042066 Zm00001d015274 Zm00001d027869 Zm00001d002523 Zm00001d019441 Zm00001d020486 Zm00001d014360 Zm00001d008198 Zm00001d029173 Zm00001d007259 Zm00001d028555 Zm00001d043666 Zm00001d044213 Zm00001d048892 Zm00001d002341 Zm00001d044365 Zm00001d050434 Zm00001d003556 Zm00001d044099 Zm00001d043539 Zm00001d010802 Zm00001d047292 Zm00001d048175 Zm00001d043392 Zm00001d047937 Zm00001d032908 Zm00001d033559 Zm00001d044088 Zm00001d032272 Zm00001d016584 Zm00001d032554 Zm00001d049109 Zm00001d025300 Zm00001d039365 Zm00001d027790 Zm00001d041255 Zm00001d034520 Zm00001d038142 Zm00001d030690 Zm00001d010619 Zm00001d011816 Zm00001d021526 Zm00001d053857 Zm00001d049443 Zm00001d014771 Zm00001d035166 Zm00001d026165 Zm00001d051394 Zm00001d044501 Zm00001d013253 Zm00001d010135 Zm00001d025786 Zm00001d052396 Zm00001d044089 Zm00001d031204 Zm00001d017347 Zm00001d044469 Zm00001d032852 Zm00001d035664 Zm00001d007675 Zm00001d012147 Zm00001d047841 Zm00001d039526 Zm00001d030532 Zm00001d051345 Zm00001d017544 Zm00001d010146 Zm00001d019180 Zm00001d044255 Zm00001d046723 Zm00001d021951 Zm00001d018863 Zm00001d053298 Zm00001d012734 Zm00001d002945 Zm00001d005403 Zm00001d028054 Zm00001d016993 Zm00001d023943 Zm00001d042722 Zm00001d044724 Zm00001d028401 Zm00001d040561 Zm00001d016301 Zm00001d022308 Zm00001d018146 Zm00001d002558 Zm00001d036356 Zm00001d033860 Zm00001d049956 Zm00001d009826 Zm00001d046456 Zm00001d010877 Zm00001d025904 Zm00001d022035 Zm00001d018360 Zm00001d044653 Zm00001d014610 Zm00001d037877 Zm00001d040638 Zm00001d022264 Zm00001d002501 Zm00001d013147 Zm00001d010021 Zm00001d024755 Zm00001d005182 Zm00001d020357 Zm00001d021371 Zm00001d012988 Zm00001d017361 Zm00001d034733 Zm00001d014194 Zm00001d048826 Zm00001d014887 Zm00001d005789 Zm00001d032215 Zm00001d047961 Zm00001d048110 Zm00001d036673 Zm00001d014187 Zm00001d037482 Zm00001d028736 Zm00001d017270 Zm00001d021646 Zm00001d041013 Zm00001d017501 Zm00001d005481 Zm00001d010341 Zm00001d003495 Zm00001d009666 Zm00001d039144 Zm00001d032282 Zm00001d005246 Zm00001d029251 Zm00001d007032 Zm00001d038984 Zm00001d007435 Zm00001d026469 Zm00001d031445 Zm00001d039050 Zm00001d049617 Zm00001d024693 Zm00001d019467 Zm00001d029137 Zm00001d018005 Zm00001d006008 Zm00001d049656 Zm00001d038355 Zm00001d005653 Zm00001d032356 Zm00001d034256 Zm00001d021197 Zm00001d008277 Zm00001d053401 Zm00001d006657 Zm00001d021218 Zm00001d033843 Zm00001d035670 Zm00001d019249 Zm00001d013419 Zm00001d033414 Zm00001d043423 Zm00001d026647 Zm00001d014140 Zm00001d049921 Zm00001d029570 Zm00001d012539 Zm00001d023426 Zm00001d016651 Zm00001d020001 Zm00001d052760 Zm00001d052439 Zm00001d038543 Zm00001d038065 Zm00001d017988 Zm00001d036206 Zm00001d002157 Zm00001d047354 Zm00001d036403 Zm00001d030185 Zm00001d052689 Zm00001d009765 Zm00001d015385 Zm00001d051871 Zm00001d044602 Zm00001d047685 Zm00001d021426 Zm00001d039514 Zm00001d021064 Zm00001d036337 Zm00001d008816 Zm00001d042582 Zm00001d018036 Zm00001d010529 Zm00001d037412 Zm00001d047282 Zm00001d026047 Zm00001d002156 Zm00001d006726 Zm00001d040535 Zm00001d028963 Zm00001d038671 Zm00001d019553 Zm00001d007302 Zm00001d001898 Zm00001d049148 Zm00001d043620 Zm00001d016619 Zm00001d023922 Zm00001d053564 Zm00001d021456 Zm00001d025268 Zm00001d009187 Zm00001d015581 Zm00001d053585 Zm00001d018445 Zm00001d053379 Zm00001d035323 Zm00001d030019 Zm00001d043012 Zm00001d032385 Zm00001d042303 Zm00001d047989 Zm00001d046740 Zm00001d019472 Zm00001d014843 Zm00001d002350 Zm00001d013571 Zm00001d012934 Zm00001d043612 Zm00001d008354 Zm00001d013309 Zm00001d053427 Zm00001d026587 Zm00001d022139 Zm00001d046703 Zm00001d021424 Zm00001d022599 Zm00001d050256 Zm00001d049798 Zm00001d050166 Zm00001d013497 Zm00001d026607 Zm00001d049685 Zm00001d036322 Zm00001d037530 Zm00001d043462 Zm00001d030207 Zm00001d026051 Zm00001d047203 Zm00001d032699 Zm00001d028885 Zm00001d031720 Zm00001d048098 Zm00001d015474 Zm00001d036563 Zm00001d048315 Zm00001d047265 Zm00001d046170 Zm00001d013615 Zm00001d041464 Zm00001d022268 Zm00001d030862 Zm00001d038290 Zm00001d039521 Zm00001d047253 Zm00001d043953 Zm00001d041545 Zm00001d052683 Zm00001d012260 Zm00001d024966 Zm00001d011392 Zm00001d040726 Zm00001d022176 Zm00001d006226 Zm00001d013481 Zm00001d014021 Zm00001d012590 Zm00001d023524 Zm00001d012702 Zm00001d002166 Zm00001d033002 Zm00001d027801 Zm00001d051876 Zm00001d012284 Zm00001d034505 Zm00001d054070 Zm00001d047681 Zm00001d042712 Zm00001d025062 Zm00001d033575 Zm00001d023343 Zm00001d032049 Zm00001d044929 Zm00001d010527 Zm00001d041258 Zm00001d048481 Zm00001d027799 Zm00001d025274 Zm00001d032708 Zm00001d047780 Zm00001d020948 Zm00001d034809 Zm00001d012766 Zm00001d005146 Zm00001d043946 Zm00001d032894 Zm00001d023510 Zm00001d046754 Zm00001d021162 Zm00001d027455 Zm00001d009417 Zm00001d012476 Zm00001d015328 Zm00001d012019 Zm00001d046972 Zm00001d040670 Zm00001d006928 Zm00001d017049 Zm00001d045341 Zm00001d039565 Zm00001d051632 Zm00001d015986 Zm00001d021895 Zm00001d022585 Zm00001d008222 Zm00001d002993 Zm00001d042451 Zm00001d013709 Zm00001d003941 Zm00001d004993 Zm00001d015557 Zm00001d010784 Zm00001d010620 Zm00001d024708 Zm00001d047971 Zm00001d003317 Zm00001d052298 Zm00001d040823 Zm00001d053855 Zm00001d026701 Zm00001d042621 Zm00001d021754 Zm00001d007010 Zm00001d020950 Zm00001d005831 Zm00001d037603 Zm00001d041900 Zm00001d003852 Zm00001d029044 Zm00001d025239 Zm00001d013597 Zm00001d025015 Zm00001d038357 Zm00001d013927 Zm00001d028653 Zm00001d023411 Zm00001d038766 Zm00001d043831 Zm00001d021709 Zm00001d042724 Zm00001d012898 Zm00001d013417 Zm00001d034023 Zm00001d053761 Zm00001d009753 Zm00001d016802 Zm00001d023749 Zm00001d017862 Zm00001d013082 Zm00001d011732 Zm00001d023317 Zm00001d040681 Zm00001d020476 Zm00001d042064 Zm00001d028097 Zm00001d005161 Zm00001d015097 Zm00001d022251 Zm00001d034750 Zm00001d004164 Zm00001d013631 Zm00001d005085 Zm00001d021050 Zm00001d034503 Zm00001d052197 Zm00001d037333 Zm00001d007907 Zm00001d019263 Zm00001d008962 Zm00001d028861 Zm00001d037397 Zm00001d038819 Zm00001d008690 Zm00001d002734 Zm00001d043868 Zm00001d013986 Zm00001d012840 Zm00001d009554 Zm00001d047242 Zm00001d047597 Zm00001d044860 Zm00001d045063 Zm00001d028678 Zm00001d021199 Zm00001d020182 Zm00001d012459 Zm00001d014178 Zm00001d026625 Zm00001d012913 Zm00001d038932 Zm00001d015837 Zm00001d009488 Zm00001d035003 Zm00001d015634 Zm00001d047800 Zm00001d034568 Zm00001d054094 Zm00001d034856 Zm00001d031957 Zm00001d018557 Zm00001d014609 Zm00001d002339 Zm00001d012295 Zm00001d025931 Zm00001d052753 Zm00001d044156 Zm00001d027834 Zm00001d014428 Zm00001d015247 Zm00001d013182 Zm00001d010259 Zm00001d048189 Zm00001d011561 Zm00001d017157 Zm00001d012620 Zm00001d046400 Zm00001d010048 Zm00001d049419 Zm00001d048823 Zm00001d024916 Zm00001d013838 Zm00001d022429 Zm00001d031741 Zm00001d035693 Zm00001d040334 Zm00001d044374 Zm00001d003750 Zm00001d029233 Zm00001d025717 Zm00001d011195 Zm00001d012710 Zm00001d011628 Zm00001d017057 Zm00001d013187 Zm00001d018113 Zm00001d047447 Zm00001d004856 Zm00001d048112 Zm00001d008596 Zm00001d011779 Zm00001d032719 Zm00001d003076 Zm00001d029782 Zm00001d011585 Zm00001d012786 Zm00001d030012 Zm00001d016158 Zm00001d009848 Zm00001d002182 Zm00001d021137 Zm00001d019637 Zm00001d005516 Zm00001d033328 Zm00001d018088 Zm00001d002739 Zm00001d013804 Zm00001d002593 Zm00001d021485 Zm00001d014302 Zm00001d048202 Zm00001d046835 Zm00001d050116 Zm00001d009740 Zm00001d027925 Zm00001d020147 Zm00001d030996 Zm00001d026030 Zm00001d036749 Zm00001d022031 Zm00001d045054 Zm00001d010401 Zm00001d040837 Zm00001d002408 Zm00001d011718 Zm00001d029135 Zm00001d019228 Zm00001d049557 Zm00001d042034 Zm00001d024239 Zm00001d042923 Zm00001d003242 Zm00001d002532 Zm00001d029534 Zm00001d047103 Zm00001d030379 Zm00001d038704 Zm00001d036447 Zm00001d024043 Zm00001d009779 Zm00001d024430 Zm00001d002969 Zm00001d017171 Zm00001d033187 Zm00001d041444 Zm00001d006192 Zm00001d008377 Zm00001d049995 Zm00001d005460 Zm00001d047762 Zm00001d011604 Zm00001d018470 Zm00001d049826 Zm00001d042043 Zm00001d006762 Zm00001d037970 Zm00001d013192 Zm00001d035838 Zm00001d047894 Zm00001d015404 Zm00001d049134 Zm00001d011035 Zm00001d031049 Zm00001d035899 Zm00001d025129 Zm00001d034411 Zm00001d024148 Zm00001d044270 Zm00001d052030 Zm00001d008428 Zm00001d029435 Zm00001d052939 Zm00001d050357 Zm00001d026071 Zm00001d015120 Zm00001d037613 Zm00001d047017 Zm00001d028143 Zm00001d014512 Zm00001d052044 Zm00001d052057 Zm00001d028152 Zm00001d015168 Zm00001d043707 Zm00001d014850 Zm00001d005090 Zm00001d003928 Zm00001d053305 Zm00001d011541 Zm00001d026374 Zm00001d014974 Zm00001d022489 Zm00001d005715 Zm00001d021242 Zm00001d029177 Zm00001d004894 Zm00001d042530 Zm00001d025182 Zm00001d011081 Zm00001d043367 Zm00001d043425 Zm00001d004167 Zm00001d031589 Zm00001d035473 Zm00001d006512 Zm00001d046513 Zm00001d018102 Zm00001d050633 Zm00001d035874 Zm00001d001974 Zm00001d050735 Zm00001d014781 Zm00001d039539 Zm00001d029497 Zm00001d053610 Zm00001d043329 Zm00001d048360 Zm00001d022230 Zm00001d019950 Zm00001d017214 Zm00001d010128 Zm00001d051457 Zm00001d024967 Zm00001d053334 Zm00001d044247 Zm00001d006480 Zm00001d053853 Zm00001d034724 Zm00001d012574 Zm00001d038877 Zm00001d009077 Zm00001d021652 Zm00001d020703 Zm00001d012660 Zm00001d020722 Zm00001d034160 Zm00001d016521 Zm00001d040348 Zm00001d045610 Zm00001d003685 Zm00001d030620 Zm00001d029093 Zm00001d017784 Zm00001d020571 Zm00001d032970 Zm00001d045649 Zm00001d012400 Zm00001d013648 Zm00001d049008 Zm00001d042879 Zm00001d003632 Zm00001d042096 Zm00001d027701 Zm00001d039016 Zm00001d036151 Zm00001d002584 Zm00001d045477 Zm00001d044475 Zm00001d025380 Zm00001d043075 Zm00001d028687 Zm00001d032956 Zm00001d037248 Zm00001d040941 Zm00001d029860 Zm00001d052113 Zm00001d015431 Zm00001d045127 Zm00001d037173 Zm00001d051430 Zm00001d002880 Zm00001d044553 Zm00001d002119 Zm00001d026547 Zm00001d007962 Zm00001d010261 Zm00001d013058 Zm00001d007085 Zm00001d038928 Zm00001d032547 Zm00001d020578 Zm00001d037830 Zm00001d043473 Zm00001d004651 Zm00001d040603 Zm00001d039285 Zm00001d027572 Zm00001d049184 Zm00001d034019 Zm00001d028183 Zm00001d027359 Zm00001d053640 Zm00001d023537 Zm00001d034601 Zm00001d040010 Zm00001d043981 Zm00001d029733 Zm00001d034591 Zm00001d012088 Zm00001d003427 Zm00001d045838 Zm00001d043693 Zm00001d003742 Zm00001d047745 Zm00001d049948 Zm00001d050340 Zm00001d035913 Zm00001d031825 Zm00001d039355 Zm00001d040362 Zm00001d042669 Zm00001d038734 Zm00001d003813 Zm00001d005513 Zm00001d020908 Zm00001d052206 Zm00001d032407 Zm00001d005346 Zm00001d006472 Zm00001d047720 Zm00001d022536 Zm00001d048191 Zm00001d012809 Zm00001d037507 Zm00001d030018 Zm00001d017427 Zm00001d028355 Zm00001d015806 Zm00001d053738 Zm00001d011492 Zm00001d051964 Zm00001d019287 Zm00001d028425 Zm00001d027726 Zm00001d038351 Zm00001d044579 Zm00001d012015 Zm00001d042828 Zm00001d054088 Zm00001d012307 Zm00001d036748 Zm00001d033890 Zm00001d042929 Zm00001d052998 Zm00001d047637 Zm00001d006497 Zm00001d049652 Zm00001d026111 Zm00001d048121 Zm00001d045455 Zm00001d009504 Zm00001d044261 Zm00001d048234 Zm00001d028088 Zm00001d005675 Zm00001d017529 Zm00001d025267 Zm00001d009669 Zm00001d026036 Zm00001d035500 Zm00001d007819 Zm00001d053150 Zm00001d018206 Zm00001d052453 Zm00001d008893 Zm00001d021480 Zm00001d045072 Zm00001d016228 Zm00001d029507 Zm00001d032985 Zm00001d011990 Zm00001d002153 Zm00001d039260 Zm00001d023367 Zm00001d036642 Zm00001d015829 Zm00001d002132 Zm00001d024222 Zm00001d017786 Zm00001d009800 Zm00001d051761 Zm00001d035288 Zm00001d013785 Zm00001d046120 Zm00001d002737 Zm00001d020411 Zm00001d006722 Zm00001d025385 Zm00001d014153 Zm00001d020989 Zm00001d011999 Zm00001d045112 Zm00001d009212 Zm00001d003839 Zm00001d017169 Zm00001d039110 Zm00001d039219 Zm00001d042398 Zm00001d032263 Zm00001d019106 Zm00001d020340 Zm00001d040429 Zm00001d039020 Zm00001d053346 Zm00001d018060 Zm00001d016891 Zm00001d028511 Zm00001d018325 Zm00001d050393 Zm00001d051987 Zm00001d017326 Zm00001d028272 Zm00001d020530 Zm00001d049672 Zm00001d006137 Zm00001d010910 Zm00001d012916 Zm00001d002240 Zm00001d011229 Zm00001d051571 Zm00001d038865 Zm00001d024759 Zm00001d026301 Zm00001d020947 Zm00001d052230 Zm00001d028955 Zm00001d042742 Zm00001d028613 Zm00001d021881 Zm00001d028009 Zm00001d003220 Zm00001d045203 Zm00001d008625 Zm00001d038139 Zm00001 |

**Table S6.** Overlapping metabolites between GM-ZD958 and its parent line as well as non-GM Z58 in growth chamber.

| **Names** | **total** | **elements** |
| --- | --- | --- |
| Beijing Zhengzhou Harbin | 8 | L-Sorbose Fluconazole Chlorogenic acid D-Fructose 1-Naphthol L-Alanine Choline Val-Ile L-Aspartate Astragalin trans-3-Coumaric acid 4-Guanidinobutyric acid Isomaltose 5-L-Glutamyl-L-alanine DL-Indole-3-lactic acid Retinol (Vitamin A) |
| Beijing Harbin | 11 | Protoporphyrin IX L-Ascorbic acid Glycerophosphocholine L-Threonine Diethanolamine Sebacic acid D-Aspartic acid D-Galactarate PC(16:0/16:0) Sunitinib D-Allose m-Chlorohippuric acid 3-Dehydroshikimic acid 5(S)-HETE L-Arginine Traumatic Acid Dihydroxyacetone D-Biotin cis-Aconitate Trehalose 3',5'-Cyclic guanosine monophosphate Arbutin D-Mannose Manumycin A 2,3-Dihydroxy-3-methylbutyric acid Shikimate L-Gulonic gamma-lactone D-Pipecolinic acid Flavin mononucleotide (FMN) APIIN |
| Beijing Zhengzhou | 284 | Lys-Leu Uridine diphosphate glucose(UDP-D-Glucose) 20-Hydroxyarachidonic acid (+)-Catechin Lys-Pro Guanidine L-Pyroglutamic acid L-Carnitine N-Acetyl-D-glucosamine L-Serine Matairesinol 1-Palmitoyl-2-oleoyl-phosphatidylglycerol Linoleic acid L-Lysine Nomilin Embelin N-Acetyl-D-Glucosamine 6-Phosphate Alantolactone N6,N6,N6-Trimethyl-L-lysine beta-Nicotinamide D-ribonucleotide Stearic acid 1-Stearoyl-sn-glycerol 3-phosphocholine Norharmane Cytidine Cytosine Eicosapentaenoic acid D-Mannose 1-phosphate Tetracosanoic acid N-Acetyl-L-glutamic acid N-.alpha.-Acetyl-L-arginine Dopamine N-Acetyl-L-tyrosine Citrate 1-Palmitoyl-2-linoleoyl-sn-glycero-3-phosphate Triethanolamine Acetylcarnitine MG(18:2(9Z,12Z)/0:0/0:0)[rac] N2,N2-Dimethylguanosine Inosine 2-Oxoadipic acid Uridine 1-Aminocyclopropanecarboxylic acid Raffinose UDP-D-Galactose |
| Zhengzhou Harbin | 5 | D-Lyxose 2-Hydroxyadenine 13(S)-HOTrE Kynurenic acid Norethindrone Acetate UDP-N-acetylglucosamine Testosterone propionate Eicosapentaenoic Acid ethyl ester Perseitol L-Methionine |
| Beijing | 132 | 11(Z),14(Z),17(Z)-Eicosatrienoic Acid Kaempferol Malvidin 3-O-glucoside cation 1-Palmitoyl Lysophosphatidic Acid Dimethylglycine Formylanthranilic acid D-Quinovose Palmitic acid Uridine 5'-monophosphate (UMP) Arg-Ala DL-2-Aminooctanoic acid Echinacoside 3-Hydroxy-4-methoxycinnamic acid Vitexin 6-Aminocaproic acid 1,3,5-Benzenetriol Citramalic acid Deoxyadenosine Cellobiose Methyl linolenate L-Tryptophan Glycine Pectin (Galacturonic acid) Glycerol 3-phosphate Xylitol L-Asparagine 1-Oleoyl-sn-glycero-3-phosphocholine L-Iditol Acetylcholine myo-Inositol Methyl 4-hydroxybenzoate 4-Aminobenzoate 1-Oleoyl-sn-glycerol 3-phosphate Heptadecanoic acid Phenylethylamine Quercetin 3'-methyl ether D-Lyxose 2-Hydroxyadenine 13(S)-HOTrE Kynurenic acid Norethindrone Acetate UDP-N-acetylglucosamine Testosterone propionate Eicosapentaenoic Acid ethyl ester Perseitol L-Methionine |
| Harbin | 505 | Pyridoxal (Vitamin B6) 2-Isopropylmalic acid Pyridoxine Lys-Leu Uridine diphosphate glucose(UDP-D-Glucose) 20-Hydroxyarachidonic acid (+)-Catechin Lys-Pro Guanidine L-Pyroglutamic acid L-Carnitine N-Acetyl-D-glucosamine L-Serine Matairesinol 1-Palmitoyl-2-oleoyl-phosphatidylglycerol Linoleic acid L-Lysine Nomilin Embelin N-Acetyl-D-Glucosamine 6-Phosphate Alantolactone N6,N6,N6-Trimethyl-L-lysine beta-Nicotinamide D-ribonucleotide Stearic acid 1-Stearoyl-sn-glycerol 3-phosphocholine Norharmane Cytidine Cytosine Eicosapentaenoic acid D-Mannose 1-phosphate Tetracosanoic acid N-Acetyl-L-glutamic acid N-.alpha.-Acetyl-L-arginine Dopamine N-Acetyl-L-tyrosine Citrate 1-Palmitoyl-2-linoleoyl-sn-glycero-3-phosphate Triethanolamine Acetylcarnitine MG(18:2(9Z,12Z)/0:0/0:0)[rac] N2,N2-Dimethylguanosine Inosine 2-Oxoadipic acid Uridine 1-Aminocyclopropanecarboxylic acid Raffinose UDP-D-Galactose |
| Zhengzhou | 143 | D-galacturonic acid all cis-(6,9,12)-Linolenic acid DL-3-Hydroxybutyric acid Pro-Asp (4Z,7Z,10Z,13Z,16Z,19Z)-4,7,10,13,1 6,19-Docosahexaenoic acid 3.alpha.-Mannobiose Leu-Leu Diosmetin Uridine 5'-diphosphate (UDP) Allantoin Thiamine Glyceric acid sn-Glycerol 1-phosphate trans-cinnamate alpha-Linolenic acid Metaraminol Perillyl alcohol L-Malic acid Pro-Glu D-Ribose 5-phosphate Apigenin Uracil L-Glutamate 4-acetamidobutanoate trans-Aconitic acid Orotate 1-Palmitoyl-sn-glycero-3-phosphocholine D-Glucose 6-phosphate Quinate Quinic acid Larixinic Acid Cyclohexylamine Hieracin Adenosine 3',5'-cyclic phosphate (cAMP) Urea Vanillin 1-Palmitoyl-2-hydroxy-sn-glycero-3-phosphoethanolamine Adenosine monophosphate (AMP) Myristoleic acid Ethanolamine Hydrocortisone Phytosphingosine 2'-Deoxyguanosine 5'-monophosphate (dGMP) Tolbutamide PC(20:5(5Z,8Z,11Z,14Z,17Z)/20:5(5Z,8Z,11Z,14Z,17Z)) Kaempferol 3-O-rutinoside Stearidonic Acid Pantothenate Rutin Anthranilic acid (Vitamin L1) (3-Carboxypropyl)trimethylammonium cation N-Acetyl-D-lactosamine Camphor 1-Palmitoylglycerol D-gluconate Riboflavin S-Methyl-5'-thioadenosine 2-C-Methyl-D-erythritol 2,4-cyclodiphosphate Citraconic acid Phthalic acid Mono-2-ethylhexyl Ester Tyramine Betaine N6-Methyl-L-lysine D-Proline 4-Aminobutyric acid Pargyline alpha-D-Glucose 1-phosphate Fludrocortisone acetate Diosmin 1-O-(cis-9-Octadecenyl)-2-O-acetyl-sn-glycero-3-phosphocholine Jasmine lactone Erucamide 2-Ethoxyethanol Narcissin 4-Hydroxycinnamic acid Thioetheramide-PC L-Sorbose Fluconazole Chlorogenic acid D-Fructose 1-Naphthol L-Alanine Choline Val-Ile L-Aspartate Astragalin trans-3-Coumaric acid 4-Guanidinobutyric acid Isomaltose 5-L-Glutamyl-L-alanine DL-Indole-3-lactic acid Retinol (Vitamin A) |

**Supporting dataset 1.** qRT-PCR cycle number (Ct values) for the differentially expressed genes in different maize lines. Data were used to produce Figure 4.

| Maize lines | Replicates | Reference and target gene | Ct_values | | | Reference and target gene | Ct_values | | | Reference and target gene | Ct_values | | |
| --- | --- | --- | --- | --- | --- | --- | --- | --- | --- | --- | --- | --- | --- |
|  |  |  | Biol.Rep_1 | Biol.Rep_2 | Biol.Rep_3 |  | Biol.Rep_1 | Biol.Rep_2 | Biol.Rep_3 |  | Biol.Rep_1 | Biol.Rep_2 | Biol.Rep_3 |
| Event2.4 | tech.Rep_1 | actin | 22.01 | 23.31 | 23.31 | actin | 22.01 | 23.31 | 23.31 | actin | 22.01 | 23.31 | 23.31 |
|  | tech.Rep_2 | actin | 21.92 | 23.01 | 23.01 | actin | 21.92 | 23.01 | 23.01 | actin | 21.92 | 23.01 | 23.01 |
|  | tech.Rep_3 | actin | 21.94 | 22.91 | 22.91 | actin | 21.94 | 22.91 | 22.91 | actin | 21.94 | 22.91 | 22.91 |
| Event3.5 | tech.Rep_1 | actin | 22.27 | 23.79 | 23.79 | actin | 22.27 | 23.79 | 23.79 | actin | 22.27 | 23.79 | 23.79 |
|  | tech.Rep_2 | actin | 21.96 | 23.61 | 23.61 | actin | 21.96 | 23.61 | 23.61 | actin | 21.96 | 23.61 | 23.61 |
|  | tech.Rep_3 | actin | 21.91 | 23.19 | 23.19 | actin | 21.91 | 23.19 | 23.19 | actin | 21.91 | 23.19 | 23.19 |
| ZD958 | tech.Rep_1 | actin | 22.52 | 23.49 | 22.01 | actin | 22.52 | 23.49 | 22.01 | actin | 22.52 | 23.49 | 22.01 |
|  | tech.Rep_2 | actin | 22.44 | 23.60 | 22.92 | actin | 22.44 | 23.60 | 22.92 | actin | 22.44 | 23.60 | 22.92 |
|  | tech.Rep_3 | actin | 22.43 | 23.19 | 22.94 | actin | 22.43 | 23.19 | 22.94 | actin | 22.43 | 23.19 | 22.94 |
| Z58 | tech.Rep_1 | actin | 22.94 | 23.12 | 24.27 | actin | 22.94 | 23.12 | 24.27 | actin | 22.94 | 23.12 | 24.27 |
|  | tech.Rep_2 | actin | 22.77 | 23.02 | 23.96 | actin | 22.77 | 23.02 | 23.96 | actin | 22.77 | 23.02 | 23.96 |
|  | tech.Rep_3 | actin | 22.73 | 22.79 | 23.91 | actin | 22.73 | 22.79 | 23.91 | actin | 22.73 | 22.79 | 23.91 |
| Chang7-2 | tech.Rep_1 | actin | 23.91 | 23.37 | 22.91 | actin | 23.91 | 23.37 | 22.91 | actin | 23.91 | 23.37 | 22.91 |
|  | tech.Rep_2 | actin | 23.83 | 23.25 | 22.73 | actin | 23.83 | 23.25 | 22.73 | actin | 23.83 | 23.25 | 22.73 |
|  | tech.Rep_3 | actin | 23.74 | 23.05 | 22.57 | actin | 23.74 | 23.05 | 22.57 | actin | 23.74 | 23.05 | 22.57 |
| Event2.4 | tech.Rep_1 | Zm00001d001911 | 21.46 | 22.99 | 21.99 | Zm00001d009709 | 21.11 | 22.36 | 20.41 | Zm00001d012446 | 22.46 | 22.99 | 24.31 |
|  | tech.Rep_2 | Zm00001d001911 | 21.46 | 21.96 | 22.46 | Zm00001d009709 | 21.01 | 22.26 | 20.31 | Zm00001d012446 | 22.46 | 22.96 | 24.01 |
|  | tech.Rep_3 | Zm00001d001911 | 21.49 | 21.98 | 21.38 | Zm00001d009709 | 20.95 | 22.23 | 20.25 | Zm00001d012446 | 22.49 | 22.98 | 23.91 |
| Event3.5 | tech.Rep_1 | Zm00001d001911 | 21.01 | 22.77 | 22.77 | Zm00001d009709 | 20.77 | 20.93 | 20.77 | Zm00001d012446 | 24.01 | 21.77 | 22.79 |
|  | tech.Rep_2 | Zm00001d001911 | 21.95 | 23.68 | 22.68 | Zm00001d009709 | 20.71 | 20.88 | 20.71 | Zm00001d012446 | 23.95 | 21.68 | 22.61 |
|  | tech.Rep_3 | Zm00001d001911 | 22.92 | 22.77 | 22.77 | Zm00001d009709 | 20.63 | 20.87 | 20.63 | Zm00001d012446 | 23.92 | 21.77 | 22.19 |
| ZD958 | tech.Rep_1 | Zm00001d001911 | 34.92 | 30.41 | 23.46 | Zm00001d009709 | 23.43 | 31.82 | 23.43 | Zm00001d012446 | 34.92 | 30.41 | 30.01 |
|  | tech.Rep_2 | Zm00001d001911 | 33.95 | 30.69 | 23.46 | Zm00001d009709 | 23.49 | 31.61 | 23.49 | Zm00001d012446 | 33.95 | 30.69 | 29.92 |
|  | tech.Rep_3 | Zm00001d001911 | 34.28 | 30.65 | 23.49 | Zm00001d009709 | 23.54 | 32.04 | 23.54 | Zm00001d012446 | 34.28 | 30.65 | 29.94 |
| Z58 | tech.Rep_1 | Zm00001d001911 | 34.06 | 30.95 | 25.01 | Zm00001d009709 | 25.62 | 31.57 | 25.62 | Zm00001d012446 | 34.06 | 30.95 | 32.27 |
|  | tech.Rep_2 | Zm00001d001911 | 33.95 | 30.94 | 24.95 | Zm00001d009709 | 25.50 | 31.46 | 25.50 | Zm00001d012446 | 33.95 | 30.94 | 31.96 |
|  | tech.Rep_3 | Zm00001d001911 | 34.43 | 31.09 | 24.92 | Zm00001d009709 | 25.47 | 31.69 | 25.47 | Zm00001d012446 | 34.43 | 31.09 | 31.91 |
| Chang7-2 | tech.Rep_1 | Zm00001d001911 | 34.16 | 32.03 | 22.91 | Zm00001d009709 | 23.98 | 32.30 | 23.98 | Zm00001d012446 | 34.16 | 32.03 | 30.91 |
|  | tech.Rep_2 | Zm00001d001911 | 33.58 | 31.63 | 22.90 | Zm00001d009709 | 23.92 | 31.78 | 23.92 | Zm00001d012446 | 33.58 | 31.63 | 30.73 |
|  | tech.Rep_3 | Zm00001d001911 | 33.98 | 32.22 | 22.97 | Zm00001d009709 | 23.91 | 31.87 | 23.91 | Zm00001d012446 | 33.98 | 32.22 | 30.57 |

**Supporting dataset 2.** qRT-PCR cycle number (Ct values) for the differentially expressed genes in different maize lines (continue 1). Data were used to produce Figure 4.

| Maize lines | Replicates | Reference and target gene | Ct_values | | | Reference and target gene | Ct_values | | | Reference and target gene | Ct_values | | |
| --- | --- | --- | --- | --- | --- | --- | --- | --- | --- | --- | --- | --- | --- |
|  |  |  | Biol.Rep_1 | Biol.Rep_2 | Biol.Rep_3 |  | Biol.Rep_1 | Biol.Rep_2 | Biol.Rep_3 |  | Biol.Rep_1 | Biol.Rep_2 | Biol.Rep_3 |
| Event2.4 | tech.Rep_1 | actin | 22.52 | 21.91 | 22.05 | actin | 22.52 | 21.91 | 22.05 | actin | 22.52 | 21.91 | 22.05 |
|  | tech.Rep_2 | actin | 22.44 | 21.87 | 21.87 | actin | 22.44 | 21.87 | 21.87 | actin | 22.44 | 21.87 | 21.87 |
|  | tech.Rep_3 | actin | 22.43 | 21.70 | 21.73 | actin | 22.43 | 21.70 | 21.73 | actin | 22.43 | 21.70 | 21.73 |
| Event3.5 | tech.Rep_1 | actin | 21.94 | 22.85 | 21.92 | actin | 21.94 | 22.85 | 21.92 | actin | 21.94 | 22.85 | 21.92 |
|  | tech.Rep_2 | actin | 22.80 | 22.80 | 21.71 | actin | 22.80 | 22.80 | 21.71 | actin | 22.80 | 22.80 | 21.71 |
|  | tech.Rep_3 | actin | 22.74 | 22.74 | 21.38 | actin | 22.74 | 22.74 | 21.38 | actin | 22.74 | 22.74 | 21.38 |
| ZD958 | tech.Rep_1 | actin | 22.87 | 22.79 | 22.02 | actin | 22.87 | 22.79 | 22.02 | actin | 22.87 | 22.79 | 22.02 |
|  | tech.Rep_2 | actin | 22.69 | 22.47 | 21.87 | actin | 22.69 | 22.47 | 21.87 | actin | 22.69 | 22.47 | 21.87 |
|  | tech.Rep_3 | actin | 22.53 | 22.27 | 21.92 | actin | 22.53 | 22.27 | 21.92 | actin | 22.53 | 22.27 | 21.92 |
| Z58 | tech.Rep_1 | actin | 22.27 | 20.17 | 22.27 | actin | 22.27 | 20.17 | 22.27 | actin | 22.27 | 20.17 | 22.27 |
|  | tech.Rep_2 | actin | 22.97 | 21.96 | 21.97 | actin | 22.97 | 21.96 | 21.97 | actin | 22.97 | 21.96 | 21.97 |
|  | tech.Rep_3 | actin | 22.87 | 23.89 | 21.87 | actin | 22.87 | 23.89 | 21.87 | actin | 22.87 | 23.89 | 21.87 |
| Chang7-2 | tech.Rep_1 | actin | 23.75 | 22.17 | 22.75 | actin | 23.75 | 22.17 | 22.75 | actin | 23.75 | 22.17 | 22.75 |
|  | tech.Rep_2 | actin | 22.57 | 22.14 | 22.57 | actin | 22.57 | 22.14 | 22.57 | actin | 22.57 | 22.14 | 22.57 |
|  | tech.Rep_3 | actin | 23.15 | 23.80 | 22.15 | actin | 23.15 | 23.80 | 22.15 | actin | 23.15 | 23.80 | 22.15 |
| Event2.4 | tech.Rep_1 | Zm00001d013358 | 20.97 | 21.53 | 24.02 | Zm00001d014108 | 23.44 | 22.93 | 22.93 | Zm00001d014944 | 21.44 | 22.93 | 22.93 |
|  | tech.Rep_2 | Zm00001d013358 | 20.87 | 21.48 | 23.92 | Zm00001d014108 | 23.22 | 22.85 | 22.85 | Zm00001d014944 | 21.22 | 22.85 | 22.85 |
|  | tech.Rep_3 | Zm00001d013358 | 20.82 | 21.40 | 23.90 | Zm00001d014108 | 23.00 | 22.78 | 22.78 | Zm00001d014944 | 21.00 | 22.78 | 22.78 |
| Event3.5 | tech.Rep_1 | Zm00001d013358 | 22.52 | 25.70 | 22.68 | Zm00001d014108 | 22.40 | 22.10 | 22.10 | Zm00001d014944 | 20.40 | 22.10 | 22.10 |
|  | tech.Rep_2 | Zm00001d013358 | 22.47 | 25.71 | 22.62 | Zm00001d014108 | 22.32 | 22.05 | 22.05 | Zm00001d014944 | 20.32 | 22.05 | 22.05 |
|  | tech.Rep_3 | Zm00001d013358 | 22.39 | 25.55 | 22.62 | Zm00001d014108 | 22.12 | 21.91 | 21.91 | Zm00001d014944 | 20.12 | 21.91 | 21.91 |
| ZD958 | tech.Rep_1 | Zm00001d013358 | 23.39 | 25.47 | 31.76 | Zm00001d014108 | 31.08 | 31.46 | 31.46 | Zm00001d014944 | 31.08 | 31.46 | 31.46 |
|  | tech.Rep_2 | Zm00001d013358 | 23.44 | 25.38 | 31.55 | Zm00001d014108 | 31.01 | 31.32 | 31.32 | Zm00001d014944 | 31.01 | 31.32 | 31.32 |
|  | tech.Rep_3 | Zm00001d013358 | 23.49 | 25.50 | 31.97 | Zm00001d014108 | 30.97 | 31.32 | 31.32 | Zm00001d014944 | 30.97 | 31.32 | 31.32 |
| Z58 | tech.Rep_1 | Zm00001d013358 | 25.56 | 25.33 | 31.51 | Zm00001d014108 | 31.96 | 31.08 | 31.08 | Zm00001d014944 | 31.96 | 31.08 | 31.08 |
|  | tech.Rep_2 | Zm00001d013358 | 25.45 | 25.11 | 31.39 | Zm00001d014108 | 31.90 | 31.01 | 31.01 | Zm00001d014944 | 31.90 | 31.01 | 31.01 |
|  | tech.Rep_3 | Zm00001d013358 | 25.42 | 24.95 | 31.63 | Zm00001d014108 | 31.88 | 30.97 | 30.97 | Zm00001d014944 | 31.88 | 30.97 | 30.97 |
| Chang7-2 | tech.Rep_1 | Zm00001d013358 | 23.93 | 23.47 | 32.24 | Zm00001d014108 | 31.38 | 31.96 | 31.96 | Zm00001d014944 | 31.38 | 31.96 | 31.96 |
|  | tech.Rep_2 | Zm00001d013358 | 23.87 | 23.34 | 31.71 | Zm00001d014108 | 31.07 | 31.90 | 31.90 | Zm00001d014944 | 31.07 | 31.90 | 31.90 |
|  | tech.Rep_3 | Zm00001d013358 | 23.86 | 23.27 | 31.81 | Zm00001d014108 | 30.93 | 31.88 | 31.88 | Zm00001d014944 | 30.93 | 31.88 | 31.88 |

**Supporting dataset 3.** qRT-PCR cycle number (Ct values) for the differentially expressed genes in different maize lines (continue 2). Data were used to produce Figure 4.

| Maize lines | Replicates | Reference and target gene | Ct_values | | | Reference and target gene | Ct_values | | | Reference and target gene | Ct_values | | |
| --- | --- | --- | --- | --- | --- | --- | --- | --- | --- | --- | --- | --- | --- |
|  |  |  | Biol.Rep_1 | Biol.Rep_2 | Biol.Rep_3 |  | Biol.Rep_1 | Biol.Rep_2 | Biol.Rep_3 |  | Biol.Rep_1 | Biol.Rep_2 | Biol.Rep_3 |
| Event2.4 | tech.Rep_1 | actin | 21.92 | 22.10 | 21.07 | actin | 21.92 | 22.10 | 21.07 | actin | 21.92 | 22.10 | 21.07 |
|  | tech.Rep_2 | actin | 21.87 | 21.92 | 22.27 | actin | 21.87 | 21.92 | 22.27 | actin | 21.87 | 21.92 | 22.27 |
|  | tech.Rep_3 | actin | 21.71 | 21.78 | 21.91 | actin | 21.71 | 21.78 | 21.91 | actin | 21.71 | 21.78 | 21.91 |
| Event3.5 | tech.Rep_1 | actin | 22.86 | 21.97 | 22.52 | actin | 22.86 | 21.97 | 22.52 | actin | 22.86 | 21.97 | 22.52 |
|  | tech.Rep_2 | actin | 22.81 | 21.76 | 22.43 | actin | 22.81 | 21.76 | 22.43 | actin | 22.81 | 21.76 | 22.43 |
|  | tech.Rep_3 | actin | 22.75 | 21.43 | 22.77 | actin | 22.75 | 21.43 | 22.77 | actin | 22.75 | 21.43 | 22.77 |
| ZD958 | tech.Rep_1 | actin | 22.88 | 22.07 | 22.73 | actin | 22.88 | 22.07 | 22.73 | actin | 22.88 | 22.07 | 22.73 |
|  | tech.Rep_2 | actin | 22.70 | 21.91 | 23.91 | actin | 22.70 | 21.91 | 23.91 | actin | 22.70 | 21.91 | 23.91 |
|  | tech.Rep_3 | actin | 22.54 | 21.97 | 23.83 | actin | 22.54 | 21.97 | 23.83 | actin | 22.54 | 21.97 | 23.83 |
| Z58 | tech.Rep_1 | actin | 22.27 | 21.40 | 21.32 | actin | 22.27 | 21.40 | 21.32 | actin | 22.27 | 21.40 | 21.32 |
|  | tech.Rep_2 | actin | 21.98 | 21.32 | 21.01 | actin | 21.98 | 21.32 | 21.01 | actin | 21.98 | 21.32 | 21.01 |
|  | tech.Rep_3 | actin | 21.87 | 21.08 | 21.96 | actin | 21.87 | 21.08 | 21.96 | actin | 21.87 | 21.08 | 21.96 |
| Chang7-2 | tech.Rep_1 | actin | 23.76 | 20.97 | 21.88 | actin | 23.76 | 20.97 | 21.88 | actin | 23.76 | 20.97 | 21.88 |
|  | tech.Rep_2 | actin | 23.58 | 21.96 | 21.07 | actin | 23.58 | 21.96 | 21.07 | actin | 23.58 | 21.96 | 21.07 |
|  | tech.Rep_3 | actin | 23.16 | 21.88 | 22.11 | actin | 23.16 | 21.88 | 22.11 | actin | 23.16 | 21.88 | 22.11 |
| Event2.4 | tech.Rep_1 | Zm00001d015130 | 19.63 | 22.23 | 19.01 | Zm00001d016477 | 19.73 | 22.37 | 19.13 | Zm00001d021635 | 19.72 | 22.15 | 19.17 |
|  | tech.Rep_2 | Zm00001d015130 | 19.58 | 22.14 | 18.91 | Zm00001d016477 | 19.68 | 22.27 | 19.02 | Zm00001d021635 | 19.67 | 22.05 | 19.06 |
|  | tech.Rep_3 | Zm00001d015130 | 19.50 | 22.11 | 18.86 | Zm00001d016477 | 19.59 | 22.25 | 18.97 | Zm00001d021635 | 19.59 | 22.03 | 19.01 |
| Event3.5 | tech.Rep_1 | Zm00001d015130 | 24.05 | 20.82 | 20.66 | Zm00001d016477 | 24.17 | 20.94 | 20.78 | Zm00001d021635 | 24.16 | 20.74 | 20.82 |
|  | tech.Rep_2 | Zm00001d015130 | 24.06 | 20.76 | 20.60 | Zm00001d016477 | 24.18 | 20.89 | 20.73 | Zm00001d021635 | 24.17 | 20.68 | 20.77 |
|  | tech.Rep_3 | Zm00001d015130 | 23.90 | 20.75 | 20.52 | Zm00001d016477 | 24.01 | 20.88 | 20.64 | Zm00001d021635 | 24.00 | 20.68 | 20.68 |
| ZD958 | tech.Rep_1 | Zm00001d015130 | 25.43 | 31.65 | 23.31 | Zm00001d016477 | 25.55 | 31.84 | 23.45 | Zm00001d021635 | 25.54 | 31.53 | 23.50 |
|  | tech.Rep_2 | Zm00001d015130 | 25.34 | 31.44 | 23.37 | Zm00001d016477 | 25.45 | 31.63 | 23.51 | Zm00001d021635 | 25.45 | 31.32 | 23.55 |
|  | tech.Rep_3 | Zm00001d015130 | 25.45 | 31.86 | 23.42 | Zm00001d016477 | 25.57 | 32.06 | 23.56 | Zm00001d021635 | 25.56 | 31.74 | 23.61 |
| Z58 | tech.Rep_1 | Zm00001d015130 | 25.29 | 31.40 | 25.48 | Zm00001d016477 | 25.40 | 31.59 | 25.64 | Zm00001d021635 | 25.40 | 31.28 | 25.69 |
|  | tech.Rep_2 | Zm00001d015130 | 25.06 | 31.28 | 25.37 | Zm00001d016477 | 25.18 | 31.47 | 25.52 | Zm00001d021635 | 25.17 | 31.17 | 25.57 |
|  | tech.Rep_3 | Zm00001d015130 | 24.90 | 31.52 | 25.34 | Zm00001d016477 | 25.02 | 31.71 | 25.49 | Zm00001d021635 | 25.01 | 31.40 | 25.55 |
| Chang7-2 | tech.Rep_1 | Zm00001d015130 | 31.42 | 32.13 | 23.86 | Zm00001d016477 | 31.53 | 32.32 | 24.00 | Zm00001d021635 | 31.52 | 32.01 | 24.05 |
|  | tech.Rep_2 | Zm00001d015130 | 31.29 | 31.60 | 23.79 | Zm00001d016477 | 31.40 | 31.79 | 23.94 | Zm00001d021635 | 31.39 | 31.49 | 23.99 |
|  | tech.Rep_3 | Zm00001d015130 | 31.23 | 31.70 | 23.78 | Zm00001d016477 | 31.34 | 31.89 | 23.93 | Zm00001d021635 | 31.33 | 31.58 | 23.98 |

**Supporting dataset 4.** qRT-PCR cycle number (Ct values) for the differentially expressed genes in different maize lines (continue 3). Data were used to produce Figure 4.

| Maize lines | Replicates | Reference and target gene | Ct_values | | | Reference and target gene | Ct_values | | | Reference and target gene | Ct_values | | |
| --- | --- | --- | --- | --- | --- | --- | --- | --- | --- | --- | --- | --- | --- |
|  |  |  | Biol.Rep_1 | Biol.Rep_2 | Biol.Rep_3 |  | Biol.Rep_1 | Biol.Rep_2 | Biol.Rep_3 |  | Biol.Rep_1 | Biol.Rep_2 | Biol.Rep_3 |
| Event2.4 | tech.Rep_1 | actin | 22.44 | 21.95 | 22.52 | actin | 22.44 | 21.95 | 22.52 | actin | 22.44 | 21.95 | 22.52 |
|  | tech.Rep_2 | actin | 22.22 | 21.90 | 22.44 | actin | 22.22 | 21.90 | 22.44 | actin | 22.22 | 21.90 | 22.44 |
|  | tech.Rep_3 | actin | 22.00 | 21.74 | 22.43 | actin | 22.00 | 21.74 | 22.43 | actin | 22.00 | 21.74 | 22.43 |
| Event3.5 | tech.Rep_1 | actin | 21.40 | 22.90 | 21.94 | actin | 21.40 | 22.90 | 21.94 | actin | 21.40 | 22.90 | 21.94 |
|  | tech.Rep_2 | actin | 21.32 | 22.84 | 21.77 | actin | 21.32 | 22.84 | 21.77 | actin | 21.32 | 22.84 | 21.77 |
|  | tech.Rep_3 | actin | 21.12 | 22.78 | 21.73 | actin | 21.12 | 22.78 | 21.73 | actin | 21.12 | 22.78 | 21.73 |
| ZD958 | tech.Rep_1 | actin | 21.08 | 22.91 | 21.91 | actin | 21.08 | 22.91 | 21.91 | actin | 21.08 | 22.91 | 21.91 |
|  | tech.Rep_2 | actin | 21.01 | 22.73 | 21.83 | actin | 21.01 | 22.73 | 21.83 | actin | 21.01 | 22.73 | 21.83 |
|  | tech.Rep_3 | actin | 20.97 | 22.57 | 22.74 | actin | 20.97 | 22.57 | 22.74 | actin | 20.97 | 22.57 | 22.74 |
| Z58 | tech.Rep_1 | actin | 21.96 | 22.31 | 22.01 | actin | 21.96 | 22.31 | 22.01 | actin | 21.96 | 22.31 | 22.01 |
|  | tech.Rep_2 | actin | 21.90 | 22.01 | 21.92 | actin | 21.90 | 22.01 | 21.92 | actin | 21.90 | 22.01 | 21.92 |
|  | tech.Rep_3 | actin | 21.88 | 21.91 | 21.94 | actin | 21.88 | 21.91 | 21.94 | actin | 21.88 | 21.91 | 21.94 |
| Chang7-2 | tech.Rep_1 | actin | 21.38 | 22.79 | 22.27 | actin | 21.38 | 22.79 | 22.27 | actin | 21.38 | 22.79 | 22.27 |
|  | tech.Rep_2 | actin | 21.07 | 22.61 | 21.96 | actin | 21.07 | 22.61 | 21.96 | actin | 21.07 | 22.61 | 21.96 |
|  | tech.Rep_3 | actin | 22.93 | 22.19 | 21.91 | actin | 22.93 | 22.19 | 21.91 | actin | 22.93 | 22.19 | 21.91 |
| Event2.4 | tech.Rep_1 | Zm00001d028951 | 21.93 | 21.93 | 23.44 | Zm00001d034717 | 19.72 | 22.37 | 19.12 | Zm00001d036571 | 22.44 | 22.93 | 22.93 |
|  | tech.Rep_2 | Zm00001d028951 | 21.85 | 21.85 | 23.22 | Zm00001d034717 | 19.67 | 22.27 | 19.02 | Zm00001d036571 | 22.22 | 22.85 | 22.85 |
|  | tech.Rep_3 | Zm00001d028951 | 21.78 | 21.78 | 23.00 | Zm00001d034717 | 19.59 | 22.25 | 18.97 | Zm00001d036571 | 22.00 | 22.78 | 22.78 |
| Event3.5 | tech.Rep_1 | Zm00001d028951 | 21.10 | 21.10 | 22.40 | Zm00001d034717 | 24.16 | 20.94 | 20.78 | Zm00001d036571 | 21.40 | 22.10 | 22.10 |
|  | tech.Rep_2 | Zm00001d028951 | 21.05 | 21.05 | 22.32 | Zm00001d034717 | 24.17 | 20.89 | 20.72 | Zm00001d036571 | 21.32 | 22.05 | 22.05 |
|  | tech.Rep_3 | Zm00001d028951 | 20.91 | 20.91 | 22.12 | Zm00001d034717 | 24.01 | 20.88 | 20.64 | Zm00001d036571 | 21.12 | 21.91 | 21.91 |
| ZD958 | tech.Rep_1 | Zm00001d028951 | 23.46 | 23.46 | 23.08 | Zm00001d034717 | 25.54 | 31.84 | 23.45 | Zm00001d036571 | 23.08 | 23.46 | 23.46 |
|  | tech.Rep_2 | Zm00001d028951 | 23.32 | 23.32 | 23.01 | Zm00001d034717 | 25.45 | 31.63 | 23.50 | Zm00001d036571 | 23.01 | 23.32 | 23.32 |
|  | tech.Rep_3 | Zm00001d028951 | 23.32 | 23.32 | 22.97 | Zm00001d034717 | 25.56 | 32.06 | 23.56 | Zm00001d036571 | 22.97 | 23.32 | 23.32 |
| Z58 | tech.Rep_1 | Zm00001d028951 | 23.08 | 23.08 | 23.96 | Zm00001d034717 | 25.40 | 31.59 | 25.63 | Zm00001d036571 | 23.96 | 23.08 | 23.08 |
|  | tech.Rep_2 | Zm00001d028951 | 23.01 | 23.01 | 23.90 | Zm00001d034717 | 25.17 | 31.47 | 25.52 | Zm00001d036571 | 23.90 | 23.01 | 23.01 |
|  | tech.Rep_3 | Zm00001d028951 | 22.97 | 22.97 | 23.88 | Zm00001d034717 | 25.01 | 31.71 | 25.49 | Zm00001d036571 | 23.88 | 22.97 | 22.97 |
| Chang7-2 | tech.Rep_1 | Zm00001d028951 | 23.96 | 23.96 | 23.38 | Zm00001d034717 | 23.53 | 32.32 | 24.00 | Zm00001d036571 | 23.38 | 23.96 | 23.96 |
|  | tech.Rep_2 | Zm00001d028951 | 23.90 | 23.90 | 23.07 | Zm00001d034717 | 23.40 | 31.79 | 23.93 | Zm00001d036571 | 23.07 | 23.90 | 23.90 |
|  | tech.Rep_3 | Zm00001d028951 | 23.88 | 23.88 | 22.93 | Zm00001d034717 | 23.33 | 31.89 | 23.92 | Zm00001d036571 | 22.93 | 23.88 | 23.88 |

**Supporting dataset 5.** qRT-PCR cycle number (Ct values) for the differentially expressed genes in different maize lines (continue 4). Data were used to produce Figure 4.

| Maize lines | Replicates | Reference and target gene | Ct_values | | | Reference and target gene | Ct_values | | | Reference and target gene | Ct_values | | |
| --- | --- | --- | --- | --- | --- | --- | --- | --- | --- | --- | --- | --- | --- |
|  |  |  | Biol.Rep_1 | Biol.Rep_2 | Biol.Rep_3 |  | Biol.Rep_1 | Biol.Rep_2 | Biol.Rep_3 |  | Biol.Rep_1 | Biol.Rep_2 | Biol.Rep_3 |
| Event2.4 | tech.Rep_1 | actin | 21.95 | 22.35 | 21.94 | actin | 21.95 | 22.35 | 21.94 | actin | 21.95 | 22.35 | 21.94 |
|  | tech.Rep_2 | actin | 21.90 | 22.25 | 22.27 | actin | 21.90 | 22.25 | 22.27 | actin | 21.90 | 22.25 | 22.27 |
|  | tech.Rep_3 | actin | 21.74 | 22.22 | 21.96 | actin | 21.74 | 22.22 | 21.96 | actin | 21.74 | 22.22 | 21.96 |
| Event3.5 | tech.Rep_1 | actin | 22.90 | 22.31 | 21.91 | actin | 22.90 | 22.31 | 21.91 | actin | 22.90 | 22.31 | 21.91 |
|  | tech.Rep_2 | actin | 22.84 | 22.01 | 22.01 | actin | 22.84 | 22.01 | 22.01 | actin | 22.84 | 22.01 | 22.01 |
|  | tech.Rep_3 | actin | 22.78 | 21.91 | 21.92 | actin | 22.78 | 21.91 | 21.92 | actin | 22.78 | 21.91 | 21.92 |
| ZD958 | tech.Rep_1 | actin | 22.91 | 22.91 | 21.94 | actin | 22.91 | 22.91 | 21.94 | actin | 22.91 | 22.91 | 21.94 |
|  | tech.Rep_2 | actin | 22.73 | 22.73 | 22.91 | actin | 22.73 | 22.73 | 22.91 | actin | 22.73 | 22.73 | 22.91 |
|  | tech.Rep_3 | actin | 22.57 | 22.57 | 22.73 | actin | 22.57 | 22.57 | 22.73 | actin | 22.57 | 22.57 | 22.73 |
| Z58 | tech.Rep_1 | actin | 22.31 | 22.52 | 22.57 | actin | 22.31 | 22.52 | 22.57 | actin | 22.31 | 22.52 | 22.57 |
|  | tech.Rep_2 | actin | 22.01 | 22.44 | 22.37 | actin | 22.01 | 22.44 | 22.37 | actin | 22.01 | 22.44 | 22.37 |
|  | tech.Rep_3 | actin | 22.91 | 22.43 | 22.27 | actin | 22.91 | 22.43 | 22.27 | actin | 22.91 | 22.43 | 22.27 |
| Chang7-2 | tech.Rep_1 | actin | 22.79 | 22.74 | 22.25 | actin | 22.79 | 22.74 | 22.25 | actin | 22.79 | 22.74 | 22.25 |
|  | tech.Rep_2 | actin | 22.61 | 22.01 | 22.01 | actin | 22.61 | 22.01 | 22.01 | actin | 22.61 | 22.01 | 22.01 |
|  | tech.Rep_3 | actin | 22.19 | 21.92 | 22.57 | actin | 22.19 | 21.92 | 22.57 | actin | 22.19 | 21.92 | 22.57 |
| Event2.4 | tech.Rep_1 | Zm00001d037840 | 21.70 | 22.34 | 21.10 | Zm00001d038922 | 21.99 | 21.99 | 21.46 | Zm00001d044451 | 21.11 | 22.35 | 21.89 |
|  | tech.Rep_2 | Zm00001d037840 | 21.65 | 22.24 | 22.00 | Zm00001d038922 | 21.96 | 21.96 | 21.46 | Zm00001d044451 | 21.00 | 22.25 | 21.84 |
|  | tech.Rep_3 | Zm00001d037840 | 21.57 | 22.22 | 21.94 | Zm00001d038922 | 21.98 | 21.98 | 21.49 | Zm00001d044451 | 21.95 | 22.22 | 21.76 |
| Event3.5 | tech.Rep_1 | Zm00001d037840 | 24.13 | 20.92 | 20.75 | Zm00001d038922 | 21.77 | 21.77 | 24.01 | Zm00001d044451 | 20.76 | 20.92 | 24.37 |
|  | tech.Rep_2 | Zm00001d037840 | 24.14 | 20.86 | 20.70 | Zm00001d038922 | 21.68 | 21.68 | 23.95 | Zm00001d044451 | 20.70 | 20.87 | 24.38 |
|  | tech.Rep_3 | Zm00001d037840 | 23.98 | 20.85 | 20.61 | Zm00001d038922 | 21.77 | 21.77 | 23.92 | Zm00001d044451 | 20.62 | 20.86 | 24.21 |
| ZD958 | tech.Rep_1 | Zm00001d037840 | 25.51 | 31.80 | 23.42 | Zm00001d038922 | 23.46 | 30.41 | 34.92 | Zm00001d044451 | 23.42 | 31.81 | 25.76 |
|  | tech.Rep_2 | Zm00001d037840 | 25.42 | 31.59 | 23.47 | Zm00001d038922 | 23.46 | 30.69 | 33.95 | Zm00001d044451 | 23.48 | 31.59 | 25.67 |
|  | tech.Rep_3 | Zm00001d037840 | 25.53 | 32.02 | 23.53 | Zm00001d038922 | 23.49 | 30.65 | 34.28 | Zm00001d044451 | 23.53 | 32.02 | 25.78 |
| Z58 | tech.Rep_1 | Zm00001d037840 | 25.37 | 31.55 | 25.60 | Zm00001d038922 | 25.01 | 30.95 | 34.06 | Zm00001d044451 | 25.61 | 31.55 | 25.62 |
|  | tech.Rep_2 | Zm00001d037840 | 25.14 | 31.44 | 25.48 | Zm00001d038922 | 24.95 | 30.94 | 33.95 | Zm00001d044451 | 25.49 | 31.44 | 25.39 |
|  | tech.Rep_3 | Zm00001d037840 | 24.98 | 31.67 | 25.46 | Zm00001d038922 | 24.92 | 31.09 | 34.43 | Zm00001d044451 | 25.46 | 31.68 | 25.23 |
| Chang7-2 | tech.Rep_1 | Zm00001d037840 | 23.50 | 32.28 | 23.97 | Zm00001d038922 | 22.91 | 32.03 | 34.16 | Zm00001d044451 | 23.97 | 32.29 | 23.73 |
|  | tech.Rep_2 | Zm00001d037840 | 23.37 | 31.76 | 23.90 | Zm00001d038922 | 22.90 | 31.63 | 33.58 | Zm00001d044451 | 23.91 | 31.76 | 23.60 |
|  | tech.Rep_3 | Zm00001d037840 | 23.30 | 31.85 | 23.89 | Zm00001d038922 | 22.97 | 32.22 | 33.98 | Zm00001d044451 | 23.90 | 31.86 | 23.53 |

**Supporting dataset 6.** qRT-PCR cycle number (Ct values) for the differentially expressed genes in different maize lines (continue 5). Data were used to produce Figure 4.

| Maize lines | Replicates | Reference and target gene | Ct_values | | | Reference and target gene | Ct_values | | |
| --- | --- | --- | --- | --- | --- | --- | --- | --- | --- |
|  |  |  | Biol.Rep_1 | Biol.Rep_2 | Biol.Rep_3 |  | Biol.Rep_1 | Biol.Rep_2 | Biol.Rep_3 |
| Event2.4 | tech.Rep_1 | actin | 22.01 | 22.31 | 22.52 | actin | 22.01 | 22.31 | 22.52 |
|  | tech.Rep_2 | actin | 21.92 | 22.01 | 22.44 | actin | 21.92 | 22.01 | 22.44 |
|  | tech.Rep_3 | actin | 21.94 | 21.91 | 22.43 | actin | 21.94 | 21.91 | 22.43 |
| Event3.5 | tech.Rep_1 | actin | 22.27 | 21.94 | 22.80 | actin | 22.27 | 21.94 | 22.80 |
|  | tech.Rep_2 | actin | 21.96 | 21.91 | 22.74 | actin | 21.96 | 21.91 | 22.74 |
|  | tech.Rep_3 | actin | 21.91 | 22.74 | 22.53 | actin | 21.91 | 22.74 | 22.53 |
| ZD958 | tech.Rep_1 | actin | 22.31 | 22.01 | 22.97 | actin | 22.31 | 22.01 | 22.97 |
|  | tech.Rep_2 | actin | 21.91 | 21.94 | 23.75 | actin | 21.91 | 21.94 | 23.75 |
|  | tech.Rep_3 | actin | 22.57 | 22.27 | 22.57 | actin | 22.57 | 22.27 | 22.57 |
| Z58 | tech.Rep_1 | actin | 22.44 | 21.91 | 22.85 | actin | 22.44 | 21.91 | 22.85 |
|  | tech.Rep_2 | actin | 22.01 | 21.87 | 22.79 | actin | 22.01 | 21.87 | 22.79 |
|  | tech.Rep_3 | actin | 25.73 | 21.70 | 22.27 | actin | 25.73 | 21.70 | 22.27 |
| Chang7-2 | tech.Rep_1 | actin | 22.91 | 22.80 | 22.91 | actin | 22.91 | 22.80 | 22.91 |
|  | tech.Rep_2 | actin | 22.83 | 22.79 | 22.73 | actin | 22.83 | 22.79 | 22.73 |
|  | tech.Rep_3 | actin | 22.74 | 22.14 | 22.57 | actin | 22.74 | 22.14 | 22.57 |
| Event2.4 | tech.Rep_1 | Zm00001d047502 | 21.46 | 21.99 | 22.99 | Zm00001d053936 | 21.44 | 22.93 | 22.93 |
|  | tech.Rep_2 | Zm00001d047502 | 21.46 | 21.96 | 22.96 | Zm00001d053936 | 21.22 | 22.85 | 22.85 |
|  | tech.Rep_3 | Zm00001d047502 | 21.49 | 21.98 | 22.98 | Zm00001d053936 | 21.00 | 22.78 | 22.78 |
| Event3.5 | tech.Rep_1 | Zm00001d047502 | 24.01 | 21.77 | 22.77 | Zm00001d053936 | 21.40 | 23.10 | 23.10 |
|  | tech.Rep_2 | Zm00001d047502 | 23.95 | 21.68 | 22.68 | Zm00001d053936 | 21.32 | 23.05 | 23.05 |
|  | tech.Rep_3 | Zm00001d047502 | 23.92 | 21.77 | 22.77 | Zm00001d053936 | 21.12 | 22.91 | 22.91 |
| ZD958 | tech.Rep_1 | Zm00001d047502 | 34.92 | 30.41 | 23.46 | Zm00001d053936 | 23.08 | 23.46 | 23.46 |
|  | tech.Rep_2 | Zm00001d047502 | 33.95 | 30.69 | 23.46 | Zm00001d053936 | 23.01 | 23.32 | 23.32 |
|  | tech.Rep_3 | Zm00001d047502 | 34.28 | 30.65 | 23.49 | Zm00001d053936 | 22.97 | 23.32 | 23.32 |
| Z58 | tech.Rep_1 | Zm00001d047502 | 34.06 | 30.95 | 25.01 | Zm00001d053936 | 23.96 | 23.08 | 23.08 |
|  | tech.Rep_2 | Zm00001d047502 | 33.95 | 30.94 | 24.95 | Zm00001d053936 | 23.90 | 23.01 | 23.01 |
|  | tech.Rep_3 | Zm00001d047502 | 34.43 | 31.09 | 24.92 | Zm00001d053936 | 23.88 | 22.97 | 22.97 |
| Chang7-2 | tech.Rep_1 | Zm00001d047502 | 34.16 | 32.03 | 22.91 | Zm00001d053936 | 23.38 | 23.96 | 23.96 |
|  | tech.Rep_2 | Zm00001d047502 | 33.58 | 31.63 | 22.90 | Zm00001d053936 | 23.07 | 23.90 | 23.90 |
|  | tech.Rep_3 | Zm00001d047502 | 33.98 | 32.22 | 22.97 | Zm00001d053936 | 22.93 | 23.88 | 23.88 |
